# Positive and balancing selection on *SLC18A1* gene associated with psychiatric disorders and human‐unique personality traits

**DOI:** 10.1002/evl3.81

**Published:** 2018-08-21

**Authors:** Daiki X. Sato, Masakado Kawata

**Affiliations:** ^1^ Graduate School of Life Sciences, Tohoku University Sendai 980–8578 Japan

**Keywords:** Human evolution, psychiatric disorders, personality traits, VMAT1

## Abstract

Maintenance of genetic variants susceptible to psychiatric disorders is one of the intriguing evolutionary enigmas. The present study detects three psychiatric disorder‐relevant genes (*CLSTN2*, *FAT1*, and *SLC18A1*) that have been under positive selection during the human evolution. In particular, *SLC18A1* (vesicular monoamine transporter 1; *VMAT1*) gene has a human‐unique variant (rs1390938, Thr136Ile), which is associated with bipolar disorders and/or the anxiety‐related personality traits. 136Ile shows relatively high (20–61%) frequency in non‐African populations, and Tajima's *D* reports a significant peak around the Thr136Ile site, suggesting that this polymorphism has been positively maintained by balancing selection in non‐African populations. Moreover, Coalescent simulations predict that 136Ile originated around 100,000 years ago, the time being generally associated with the Out‐of‐Africa migration of modern humans. Our study sheds new light on a gene in monoamine pathway as a strong candidate contributing to human‐unique psychological traits.

**Impact Summary**

A question as to how human‐unique characteristics have been evolved is of broad interest to biologist and the general public. To cope with this question, we focused on genes relevant to psychiatric disorders, since it has been hypothesized that the emergence of psychiatric disorders is linked to the evolution of human brain. On the other hand, most genetic variants susceptible to psychiatric disorders have relatively moderate effects and serve as a foundation of personality traits. Although there are some previous studies that aimed to detect psychiatric disorder‐relevant genes under positive selection in the human lineage, human‐unique genetic variants maintained by selection have not been explored. Such genetic variants could associate with human‐unique mental variation, such as personality traits. Here, we found a gene, *SLC18A1* (*VMAT1*: Vesicular monoamine transporter 1), as a positively selected gene in the human lineage. This gene has a human‐unique variant (Thr136Ile; different from other mammals (136Asn)) whose association with several psychotic symptoms has been repeatedly indicated. Moreover, our analysis showed that this variant has been maintained in non‐African populations by balancing selection and had originated around 100,000 years ago, typically regarded as the timing of Out‐of‐Africa migration. 136Thr has been indicated to be associated with depression and anxiety compared to 136Ile, thus it could be possible that tendency to feel uneasy have been selected during human evolution and that environmental changes accompanied with Out‐of‐Africa migration resulted in the selective advantage of 136Ile against such anxious minds, sometimes leading to psychiatric disorders. This study is the first to provide evidence that a certain sort of psychological traits in humans has been adaptively selected and that diversity of personality traits, possibly leading to psychiatric disorders, are maintained by balancing selection in the current populations.

## Introduction

Characteristics unique to humans might have evolved through natural selection, although these often resulted in disorders and diseases as by‐products of the adaptive evolution (Crespi et al. 2007; Moalic et al. 2010; Fay 2013; Ogawa and Vallender 2014). Psychiatric disorders (PDs), such as schizophrenia, autism, and depression, are generally characterized by cognitive dysfunction, social impairment, or affective disturbance and are very common in our modern society. One in five people are known to suffer from some sort of psychiatric disorder during their lifespan (Kessler et al. 2009; Steel et al. 2014). Although PDs often reduce survival rates and fertility (Keller and Miller 2006; Uher 2009; Sullivan et al. 2012), it has been reported that some disorders, such as schizophrenia, are associated with human‐unique characteristics, including complex vocal communications, creativity, and divergent thinking (Tolosa et al. 2010; Keller and Visscher 2015; Power et al. 2015). Thus, it has been proposed that some PDs have evolved as maladaptive by‐products of adaptive human evolution (Horrobin 1998; Burns 2006; Crespi et al. 2007).

Several studies have attempted to detect the signature of positive selection in the human lineage for genes contributing to PDs. Crespi et al. (2007) reported the signature of positive selection (i.e., high *d*_N_/*d*_S_ ratio) in genes associated with schizophrenia. Ogawa and Vallender (2014) analyzed the genes associated with several neurological and neuropsychiatric diseases and revealed a general trend of an elevated *d*_N_/*d*_S_ ratio in the catarrhines and cetaceans, species that have larger brains than their sister groups. These studies revealed a tendency that genes relevant to PDs (PD genes) are likely to be positively selected and supported the hypothesis that PDs are the by‐products of adaptive evolution of the human brain.

Since PDs are associated with moderate to severe social impairment and lower fertility, genetic variants linked to PDs are considered to be deleterious. However, some of these variants might not necessarily reduce the fitness of the patients. Genetic variants related to PDs might rather be maintained by natural selection, although few studies have so far detected the signature of balancing selection in the alleles contributing to PDs. For instance, in human serotonin transporter (5‐HTT), two common variants are present in the number of tandem repeats in the promoter sequence (5‐HTTLPR): a long allele, which comprises 16 tandem repeats, and a short allele, which comprises 14 tandem repeats (Nakamura et al. 2000). The long and short alleles are, in particular, associated with high and low expression levels of the serotonin transporter gene (*SLC6A4*), respectively (Greenberg et al. 1999), and low‐expressing (LE) allele carriers reveal higher risks for mental health disorders, such as anxiety and depression, than the high‐expressing (HE) allele carriers (Lesch et al. 1996; Munafò et al. 2009). Uddin et al. (2010) reported that subjects with the heterozygous allele had a reduced depression score than those with both homozygous alleles, and that the score depended on the gender and environment, suggesting the presence of balancing selection on those alleles caused by overdominance and/or gene‐by‐environment (G × E) interaction. This indicates that certain aspects of emotional differences among individuals, often leading to psychiatric symptoms, are created by the genetic variants through natural selection, although in the case of 5‐HTTLPR, the association between the genetic variant and psychiatric phenotypes is still controversial.

In psychology, the five‐factor model (neuroticism, extraversion, openness to experience, agreeableness, and conscientiousness) is commonly used to characterize the broad human personality traits (Digman 1990). A recent study reported significant genetic correlations between these personality traits and PDs (Lo et al. 2017). In regard to nonhuman primates, reports on their personality traits have been published (Freeman and Gosling 2010). Weiss et al. (2012) reported that similarities exist in the personality traits among great apes and humans, and they suggested that this similarity across species could be maintained by balancing selection. The polymorphism in 5‐HTTLPR influences the individual personality traits, such as anxiety‐related traits (Lesch et al. 1996; Minelli et al. 2011) and hopelessness (Kangelaris et al. 2010), both composing elements of neuroticism (Gonda et al. 2009), and this polymorphism can be found in other primate species (Dobson and Brent 2013). Thus, the personality traits observed in both nonhuman primates and humans could be explained by certain similar genetic variants that are related to the PDs maintained by balancing selection. However, a recent review indicated that there is no clear evidence for balancing selection in human neurotransmitter genes including *SLC6A4* (Taub and Page 2016).

Herein, we attempt to detect the PD genes that evolved from other apes and mammals through positive natural selection. In addition, we attempt to determine the variants that have been maintained in the present population by balancing selection. Detecting these variants should clarify the evolutionary process of the brain function, PDs, and personality traits specific to humans. In the present study, we aimed to (1) detect the positively selected genes among the PD genes, (2) investigate whether the candidate genes carry polymorphic alleles linking to the risk of PDs, and eventually, and (3) estimate the signature of balancing selection as an explanation for maintaining these alleles.

## Materials and Methods

### SEQUENCE PREPARATION

Ortholog information and the coding sequences of 15 mammal species, human (*Homo sapiens*), chimpanzee (*Pan troglodytes*), gorilla (*Gorilla gorilla gorilla*), orangutan (*Pongo abelli*), gibbon (*Nomascus leucogeny*s), macaque (*Macaca mulatta*), marmoset (*Callithrix jacchus*), bushbaby (*Otolemur garnettii*), mouse (*Mus musculus*), guinea pig (*Cavia porcellus*), rabbit (*Oryctolagus cuniculus*), dog (*Canis lupus familiaris*), horse (*Equus caballus*), microbat (*Myotis lucifugus*), and cow (*Bos taurus*), were obtained from the Ensembl database, release 85 (Yates et al. 2016), and 7310 one‐to‐one orthologous genes were obtained as a reference gene set. For each gene, the longest transcript sequence was chosen as the representative sequence. BlastP was used to determine the best transcript sequence only when there were two or more transcript sequences with the exact same length for a given gene.

### GENOMIC INFORMATION FOR HUMANS AND ARCHAIC HOMININS

To improve the quality of human coding sequences (CDS) and to reduce false positives in evolutionary rate analysis, we used data from the 1000 Genomes Project phase 3 (1000G) (Auton et al. 2015) comprising 2504 individuals from 26 populations worldwide. Reference alleles in human CDS were replaced by derived alleles on some occasions such as when the frequency of the reference allele was below 1% in the whole human population (calculated using all of the 2504 individuals in 1000G dataset) and/or when the ancestral allele (written in the 1000G dataset) still exists in human population (registered as a derived allele) at a high frequency above 1%. The 1000G dataset was also used to determine allele frequencies of each population and to count synonymous and nonsynonymous single nucleotide polymorphisms (SNPs) in humans. Allelic information from two Neanderthals (Prüfer et al. 2014, 2017) and a Denisovan (Meyer et al. 2012) and the map of the genomic region putatively introgressed from Neanderthals (Sankararaman et al. 2014; Vernot and Akey 2014) were used to determine whether a variant of interest belongs to a region of ancestry in archaic hominins. Intronic variants of interest were examined for their functional effects on the expression of the gene (whether they are expression quantitative trait loci (eQTL) or not) using GTEx Portal (https://www.gtexportal.org/home/).

### PSYCHIATRIC DISORDERS‐RELEVANT GENES

From a variety of PDs, we selected five major disorders to target, based on their prevalence and centrality in the psychogenetic study: Attention deficit and hyperactivity disorder (ADHD), autism spectrum disorder, bipolar disorder, major depression (unipolar) disorder, and schizophrenia (Smoller 2013). DisGeNET v4.0, a comprehensive gene–disease association database that integrates expert‐curated databases with text‐mined data (Piñero et al. 2017) was used to obtain genes relevant to those five disorders. In order to avoid dealing with the genes falsely associated with the disorders, possibly caused by low powered genome‐wide association studies and/or error in text‐mining methods and the manual curation, we only focused on the genes whose association with any of the five PDs has been reported by at least two previous studies. Through the filtering process, 588 PD genes in the reference gene set were selected for further analysis. The analyzed genes are shown in Table S1.

### SEQUENCE ALIGNMENT AND EVOLUTIONARY RATE ANALYSIS

For each amino acid sequence of the 7310 genes, an alignment was first performed by MAFFT version 7 (Katoh and Standley 2013). Subsequently, we concatenated all the aligned protein‐coding sequences of those genes and constructed a reference species tree using RAxML 8. 2. 7 (Stamatakis 2014). In the next step, the evolutionary rate analysis was conducted on the 588 genes that are relevant to the aforementioned PDs. To calculate *d*_N_/*d*_S_, we performed the finer alignment using PRANK (Löytynoja and Goldman 2005, 2008) with the reference species tree. PRANK is characterized by a phylogeny‐aware alignment and has been recommended for evolutionary rate analysis (Fletcher and Yang 2010). To predict genes and sites under positive selection, we applied the branch‐site model implemented in PAML version 4.8 (Yang 2007), which has a higher detection capability than the branch model used in a previous study (Ogawa and Vallender 2014). The branch‐site model is aimed at detecting specific amino acid sites that have been positively selected in the specific lineage that the user is interested in. It is based on a likelihood estimation, in which all lineages are classified into two types of hypothetical lineages a priori: background lineages that are assumed to have evolved neutrally, and foreground lineages that are assumed to have been under positive selection. In this study, we set up a hypothetical model where positive selection occurred in the human lineage after divergence from the common ancestor of humans and chimpanzees, performed likelihood ratio tests between the null model and the hypothetical model using the χ^2^ test, and estimated genes and sites under positive selection. Here, amino acid sites with a probability score of positive selection by Bayes Empirical Bayes analysis (Yang et al. 2005) higher than 0.5 were interpreted to be positively selected sites. QVALUE package in R was used to conduct multiple correction for the number of genes analyzed (Bass et al. 2015).

### EVALUATION OF BIOLOGICAL IMPACT FOR DETECTED AMINO ACID SUBSTITUTIONS

For the genes detected to be under positive selection in the human lineage, we evaluated the impact of human‐specific amino acid substitutions. Provean (Choi et al. 2012; Choi and Chan 2015) and SIFT (Kumar et al. 2009) both calculate the impact of amino acid substitutions based on an amino acid substitution matrix and their degree of conservation at the given sites among species. An amino acid substitution is predicted as deleterious (i.e., likely to affect its protein function) if the Provean score is less than −2.5 or the SIFT score is less than 0.05.

### McDONALD–KREITMAN TEST AND DoS STATISTIC

The McDonald–Kreitman (MK) test is one of the most powerful and popular tests used to detect a signature of positive selection on protein‐coding regions (McDonald and Kreitman 1991). It assumes synonymous substitutions as neutral and detects positive selection if the ratio of the number of nonsynonymous substitutions (*D*_N_) to synonymous ones (*D*_S_) in interspecific (human and chimpanzee or macaque) comparisons exceeds the intraspecific (human) ratio (the number of nonsynonymous polymorphisms (*P*_N_) to synonymous polymorphisms (*P*_S_) in a species). When intraspecific variation exceeds the interspecific level, diversifying selection is implied. We annotated each SNP belonging to CDS in the 1000G dataset by referencing genomic information in Ensembl and calculated *P*_N_ and *P*_S_ for each gene. *D*_N_ and *D*_S_ were calculated from the alignment of each gene set (see Sequence alignment and evolutionary rate analysis section, above). We also added the Direction of Selection (DoS) statistic (Stoletzki and Eyre‐walker 2011) into the analysis. DoS, which is obtained by subtracting *P*_N_/(*P*_S_ + *P*_N_) from *D*_N_/(*D*_S_ + *D*_N_), is a very simple but robust statistic for inferring the mode of selection and whose positive value indicates adaptive evolution, zero indicates neutral evolution and negative value indicates accumulation of slightly deleterious mutations (Stoletzki and Eyre‐walker 2011). We compared the results among the category of genes, each belonging to any one of four categories, after estimating positively selected genes from all of the 7310 genes using PAML software as mentioned above (multiple correction was not applied here). These categories were: positively selected genes related to psychiatric disorders (PD‐PSGs); positively selected genes unrelated to psychiatric disorders (NPD‐PSGs); not positively selected genes related to psychiatric disorders (PD‐NPSGs); and not positively selected genes unrelated to psychiatric disorders (NPD‐NPSGs).

### DETECTION OF BALANCING SELECTION USING TAJIMA's *D*

For the corresponding genomic regions to the PD‐PSGs, we calculated Tajima's *D* (Tajima 1989), which is often used as an index of balancing selection, using an in‐house perl script (10 kbp window with 2 kbp shifting width). Tajima's *D* is a statistic based on the difference between *θ*_W_ and *θ*_T_, where *θ*_W_ represents the number of segregating sites of a given locus, and *θ*_T_ represents the number of pairwise differences in the locus among individuals. For *SLC18A1*, two methods were used to obtain a null distribution of Tajima's *D* to determine the specific regions under balancing selection for each population: one based on the empirical distribution of Tajima's *D* across the whole genome; and one based on polymorphic data generated by simulation under the assumption of selective neutrality with a well‐documented population demography (Gravel et al. 2011) using ms (Hudson 2002). The simulations were conducted for a million times. A recombination rate of 10 kbp, with a central focus on Thr136Ile, was obtained from a HapMap Phase II genetic map (Frazer et al. 2007). Detailed information on the ms simulation is presented in Table S2. When Tajima's *D* of a given locus exceeded the 95^th^ percentile of the null distribution, we concluded that balancing selection was active.

### LINKAGE DISEQUILIBRIUM AROUND THE CANDIDATE GENES

It is considered that young balancing selection leaves a genomic signature represented by strong linkage disequilibrium (LD) with adjacent regions as well as selective sweeps (DeGiorgio et al. 2014; Fijarczyk and Babik 2015). Haploview (Barrett et al. 2005) was used to visualize LD in the candidate genomic regions in *CLSTN2* and *SLC18A1* where the peaks of Tajima's *D* were observed. Moreover, for the region around Thr136Ile polymorphism in *SLC18A1*, we calculated the nSL statistic to quantify the strength of LD around the site. nSL (Ferrer‐Admetlla et al. 2014) is a robust statistic calculated from the difference of extended haplotype homozygosity (EHH) between the alleles of interest. Taking into account that nSL is highly correlated with allele frequency (Ferrer‐Admetlla et al. 2014), we compared the nSL of Thr136Ile to that of 10,000 randomly chosen SNPs with almost the same (±1%) allele frequency in each population. Selscan version 1.2.0a (Szpiech and Hernandez 2014) was used to calculate unstandardized nSL.

### EVOLUTIONARY SIMULATIONS UNDER CONSTANT SELECTIVE PRESSURE FOR 136Ile

To confirm further that the unique genomic signature around the Thr136Ile site, as represented by high Tajima's *D* and low nSL (strong selection for 136Ile) values, is not due to a simple selective (partial) sweep without balancing selection, we conducted evolutionary simulations under the assumption of constant selective pressure for 136Ile using the SLiM simulator (Haller and Messer 2017). The same values as those used in the ms simulations were used for the basic parameter values of the simulations (see *Detection of balancing selection using Tajima's D*, above). We assumed that 136Ile had been derived from 136Thr at some point in time and had been under constant selective pressure. Simulations with different selection coefficients (ranging from 0 to 0.0039 in 0.0001 increments) and mutation ages (ranging from 2100 to 900 generations ago in 50 generation increments, which correspond to the time spanning from Out‐of‐Africa migration to the divergence between Asian and European populations in a previous model (Gravel et al. 2011)), were conducted with 1,000 replicates for each (1,000,000 simulations in total). Consequently, we collected simulations that generated 136Ile allele frequencies that approximated (±5%) those observed in all the three populations; African, European, and Asian. For the polymorphic data generated by accepted simulations, we calculated Tajima's *D* and unstandardized nSL using vcftools version 0.1.13 (Danecek et al. 2011) and selscan version 1.2.0a (Szpiech and Hernandez 2014), respectively.

### HAPLOTYPE NETWORK AND ESTIMATION OF TMRCA Of HUMAN *SLC18A1*

The global populations (YRI, CEU, CHB, and JPT) in the 1000G dataset were integrated and used as a population to estimate haploblocks using Haploview, where each SNP was in LD. We obtained 1793 bp, comprising 29 SNPs that include Thr136Ile (rs1390938) and 30 haplotypes made from these SNPs. The median‐joining network to infer the haplotype genealogy was constructed using PopART 1.7 (Leigh and Bryant 2015). Coalescent simulations were conducted using GENETREE9.0 (Griffiths and Tavare 1994a, b) to estimate the scaled population mutation rate (*θ*_ML_), growth parameter (*β*_ML_), time to the most common recent ancestor (TMRCA), and mutation age for each haplotype. We used an often quoted value of 10,000 as the effective population size of the integrated human population to translate the coalescent time into real time.

## Results

### POSITIVELY SELECTED GENES (PSGs) IN THE HUMAN LINEAGE

The results of *d*_N_/*d*_S_ analysis reported that three PD genes, *CLSTN2*, *FAT1*, and *SLC18A1*, are positively selected in the human lineage, although no genes were available after correction for multiple comparisons (Table 1). Among the PD‐PSGs, *FAT1* and *SLC18A1* have multiple sites that reveal evidence of positive selection in the human lineage (i.e., BEB score > 0.5) and had a significant impact on the protein function estimated by Provean and SIFT (Table S3). Contrary to our expectation, the MK test for the PD‐PSGs showed that the ratio of nonsynonymous to synonymous polymorphisms within humans was generally higher than the ratio of nonsynonymous to synonymous substitutions between species (human vs. chimpanzee and human vs. macaque), indicating diversifying selection acting on those genes (Table S4). DoS also exhibited lower values for the three genes, attributed to the relatively high *P*_N_/(*P*_S_ + *P*_N_) to *D*_N_/(*D*_S_ + *D*_N_), although statistical significance was not observed, possibly due to the low sample size (Fig. S1). Considering the fact that the genes related to PDs (PD‐NPSGs) were generally conserved (low values for both *P*_N_/(*P*_S_ + *P*_N_) and *D*_N_/(*D*_S_ + *D*_N_), see Fig. S1), as shown in previous study (Ogawa and Vallender 2014), the high *P*_N_/(*P*_S_ + *P*_N_) values are a unique characteristic for the PD‐PSGs. These results suggest that the PD‐PSGs could have experienced not only positive selection but also diversifying and/or balancing selection to generate and maintain genetic diversity in the corresponding genomic regions, and that this pattern is quite unique to PD‐PSGs in comparison with both the PD‐NPSGs and the NPD‐PSGs. Thus, we conducted further analyses from the population genetic perspective especially focusing on these three PD‐PSGs.

**Table 1:** Positively selected genes relevant to psychiatric disorders (PD‐PSGs) estimated by PAML

Genes	Site d*N*/d*S*	Likelihood of Null model	Likelihood of Alternative model	*χ* ^2^	*P*‐value	*q*‐value
*CLSTN2*	228.43	−9148.16	−9143.78	8.76	0.0030	1.00
*FAT1*	999.00	−62480.72	−62474.75	11.95	0.0005	0.43
*SLC18A1*	271.87	−7085.21	−7083.08	4.26	0.0390	1.00

### TAJIMA's *D* OF THE CANDIDATE GENES

**Figure 1:**
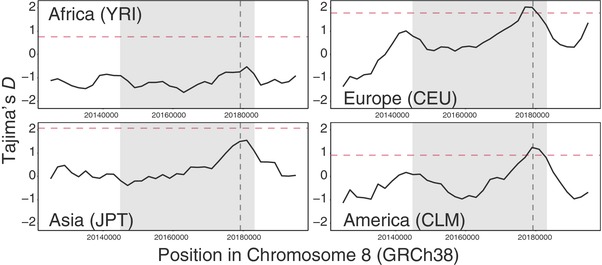
The distribution of Tajima's *D* around *SLC18A1* for each population.

We calculated Tajima's *D* for the genomic regions corresponding to the three PD‐PSGs. No significant Tajima's *D* peaks were observed in *FAT1* (Fig. S2a), while multiple significant peaks were observed in *CLSTN2*, with some belonging to exon regions, especially around exon 5 and 6 in most non‐African populations (Fig. S2b). A peak was also observed in *SLC18A1* at around exon 3 in most non‐African populations too (Fig. 1; Fig. S2c). The values in several populations, including all the European ones, significantly deviated from the genome average, which was calculated from an empirical distribution of Tajima's *D* across the whole genome for each population. When coalescent simulations were conducted to determine the upper thresholds of Tajima's *D*, the values for the Asian populations were also significant (Fig. S2c).

### LD IN THE CANDIDATE REGIONS OF *CLSTN2* AND *SLC18A1*

**Figure 2:**
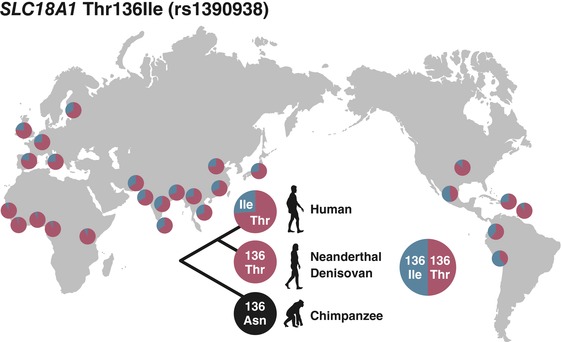
Geographic variation in the allele frequency of *SLC18A*1 Thr136Ile (rs1390938) and the genotype of Neanderthal, Denisovan, and Chimpanzee.

Subsequently, LD was examined in the specific regions of the two candidate genes, *CLSTN2* and *SLC18A1*, on which balancing selection has possibly acted. Strong LD was observed in non‐African populations (CEU, JPT, and CLM) compared to the African population (YRI; Fig. S3a and b) for all the investigated regions, which was consistent with the results for Tajima's *D*. No linked exon variants or eQTL were found in the investigated regions of *CLSTN2* and the strong LD in *CLSTN2* was possibly derived from functionally unknown intronic variants. For *SLC18A1*, on the other hand, Thr136Ile (rs1390938), a nonsynonymous polymorphism on which positive selection had just occurred (Table S3), belonged to one of the core SNPs at the central location of the peak of Tajima's *D* and LD (Fig. 1; Fig. S2). The frequencies of 136Ile in non‐African populations were generally higher (about 20–61%) than those of African populations (about 4–10%; Fig. 2), which was consistent with the observed pattern of Tajima's *D* and LD. Both alleles of this SNP were different from those of other mammalian species (i.e., a human‐unique polymorphic site, Fig. S4), and the functional and psychological effects of this SNP have been already shown (see below). Based on these important and unique features, we focused further on this SNP and quantified the strength of LD by applying the nSL statistic. Compared to randomly chosen SNPs with the same allele frequency, the Thr136Ile exhibited a significantly lower nSL score in all the global populations (Fig. S5), indicating that 136Ile has been under strong selective pressure not only in non‐African but also in African populations. From a‐million‐time evolutionary simulations, 1445 of them were accepted by their closeness to the observed 136Ile allele frequencies. The accepted simulations showed that a partial sweep for 136Ile could generate a signal with a lower nSL score (Fig. S6); however, partial sweep alone could not generate the higher Tajima's *D* score observed in non‐African populations, and especially in European populations (Fig. S7), suggesting balancing selection as a likely explanation of genomic signature around this SNP in non‐African populations.

### ESTIMATION OF HAPLOTYPE NETWORK AND MUTATION AGE OF 136Ile IN *SLC18A1*

A median‐joining haplotype network revealed a star‐like structure, indicating that H13 (containing 136Thr) was the ancestral haplotype (Fig. S8). 136Ile was found in H5, H12, H18, and H24, and the mutation from 136Thr to 136Ile is likely to have occurred in H24 (Fig. S8). This result was consistent with the observation that all the archaic hominins carried 136Thr and that the genomic regions around this SNP did not exhibited any introgression signature from Neanderthals. We conducted coalescent simulations using GENETREE and estimated the 136Ile mutation age as having originated 105,500 (SE: ±30,800) years ago (Fig. S9).

### DISCUSSION AND CONCLUSIONS

Our fine‐tuned evolutionary rate analysis revealed that few PD genes were positively selected in the human lineage, which differs from the results of a previous study (Crespi et al. 2007), and that PD genes are generally conserved as previous studies show (McLysaght et al. 2014; Ogawa and Vallender 2014). However, the three detected PD‐PSGs (*CLSTN2, FAT1*, and *SLC18A1*), exhibited the unique signature of diversifying selection, estimated from the MK test and the DoS statistic. This suggests that dysfunctional mutations that cause PDs could be positively maintained. In fact, peaks of Tajima's *D* and LD were observed in two of the three PD‐PSGs (*CLSTN2* and *SLC18A1*), allowing for the possibility that balancing selection has been acting on them. A nonsynonymous SNP (rs1390938, Thr136Ile) in *SLC18A1*, which had been subject to positive selection in the human lineage, is located at the center of the Tajima's *D* and LD peaks, suggesting that this SNP is a core driver of natural selection through the long history of human evolution. Thus, taking into account the uniqueness and putative importance of this SNP, we focus mainly on *SLC18A1* in the following discussion.

*SLC18A1* encodes the vesicular monoamine transporter 1 (VMAT1), which is involved in the uptake of monoamines, such as serotonin, dopamine, and norepinephrine into synaptic vesicles (Varoqui and Erickson 1997; Wimalasena 2010). This gene is located on chromosome 8p21.3, a strong candidate region associated with various PDs (Tabarés‐Seisdedos and Rubenstein 2009), including schizophrenia (Bly 2005; Richards et al. 2006; Lohoff et al. 2008b), bipolar disorder (Lohoff et al. 2006), and anxiety (Lohoff et al. 2008a). The first luminal loop domain (Fig. S10) of VMAT1 represents a putative receptor‐like structure and is crucial for transport of monoamine, which is mediated by G‐protein (Brunk et al. 2006). Since both substituted sites in the human lineage (Glu130Gly and Asn136Thr, see Table S3) are located in this domain, these substitutions are quite likely to affect the activity of the transporter. In fact, Thr136Ile polymorphism (rs1390938) has been previously discussed for its effects in PDs (Lohoff et al. 2006, 2008a, 2014; Khalifa et al. 2012). Lohoff et al. (2006, 2008) observed that 136Thr is associated with the bipolar disorder, and subjects with heterozygous alleles exhibited higher anxiety scores (Lohoff et al. 2006, 2008a). A recent study has also reported that 136Ile promotes monoamine transport into the synaptic vesicles, and that 136Ile increases the threat‐related amygdala reactivity (Lohoff et al. 2014).

VMAT1 has a higher affinity for serotonin than the other monoamines (Brunk et al. 2006). Serotonin is a phylogenetically ancient molecule and its circuits are involved in several central brain functions, such as mood, social or aggressive behavior, sleep, and memory. In addition, dysfunctions in its transport are major causes of a variety of PDs. Thus, its role in neurodevelopment and the effects of genotype on the resulting physiological or behavioral phenotype have been widely discussed; however, few studies have addressed its importance from the perspective of human evolution. Stimpson et al. (2016) has identified an evolutionary change in the serotonergic innervation of the amygdala between chimpanzees and bonobos, and suggested that it has led to differences in the aggressive behaviors, cautious temperaments, risk preferences, and performances on “theory of mind” tasks even between these closely related species. Raghanti et al. (2018) demonstrated that the human striatum, a region of the brain modulating social behavior, exhibits unique neurochemical profile compared to other primates. Sousa et al. (2017) have reported that a type of dopaminergic interneuron exclusively exists in the human neocortex. These studies could be evidence supporting the possibility that changes in monoaminergic circuits have driven the evolution of both the brain and human behavior. Inoue‐Murayama et al. (2001) reported that LE alleles in 5‐HTTLPR, which are associated with anxiety or depressive behavior, are found at higher frequencies in humans than in the other apes, and speculated that anxiety has been favored throughout human evolution. Since Thr136Ile affects the anxiety‐related personality traits (Lohoff et al. 2008a) and is linked to the anxiety or depressive phenotypes (Vaht et al. 2016), the evolution from Asn to Thr at the 136^th^ site of *SLC18A1* might have led to more anxious human minds through positive selection. Alvergne et al. (2010) reported that women's neuroticism was associated with a tradeoff between offspring quality and quantity in a rural Senegalese population that has characteristics of preindustrial societies. Thus, it is possible that the anxiety‐related traits in women could have been under selective pressure through the process of the human evolution.

The 14 mammals examined (excluding humans) carried Asn at the 136^th^ site of *SLC18A1*, while the archaic and modern humans carried only 136Thr or both 136Thr and 136Ile, respectively, although the genotype information was limited to a few individuals for the archaic hominins (Fig. 2). Coalescent simulations indicated that 136Ile emerged approximately 100,000 years ago, which coincides with the Out‐of‐Africa migration by modern humans (Mallick et al. 2016; Pagani et al. 2016). These results suggest that 136Thr evolved in the archaic human lineage by positive natural selection from great apes, and 136Ile increased in frequency after the modern humans migrated into Europe where it has been maintained in non‐African populations. Interestingly, African populations also exhibited the significant nSL values at Thr136Ile, although we could not detect the signature of balancing selection in these populations. In addition, evolutionary simulations assuming a partial sweep for 136Ile also showed that the simulated values of nSL and Tajima's *D* were close to the observed values in African populations (Fig. S7 and S8), indicating that a selective sweep without balancing selection may be a predominant mode of selection in African populations in contrast to non‐African populations. Taking into account these results, the form of natural selection acting on Thr136Ile may be different between African and non‐African populations. The relationship between the prevalence of the disease‐risk alleles and the Out‐of‐Africa event has been discussed widely, and it is generally accepted that a significant bottleneck at that time acted to enhance the genetic drift and to increase the frequencies of risk alleles in populations (Tishkoff and Williams 2002; Comas et al. 2013). Regarding the PDs, it is known that the prevalence of mental disorders in African populations is lower than in the other regions in the world (Gureje et al. 2006; Steel et al. 2014). Our results, combined with those of the previous studies, suggest that the variants linked to PDs not only exist due to the historical genetic drift but also could likely be maintained by balancing selection in non‐African populations.

The present study demonstrated that the polymorphic state of the 136th amino acid site (Thr136Ile) in *SLC18A1* has been maintained by balancing selection in some populations. Overdominance and negative frequency‐dependent selection are major mechanisms of balancing selection; however, G × E interaction effects of spatially or temporally varying environments could also be important mechanisms (Levene 1953; Turelli and Barton 2004; Hedrick 2006; Penke et al. 2007). Lohoff et al. (2008a) reported that German female subjects with the heterozygous (136Thr/Ile) alleles were more prone to anxiety than those with either homozygous alleles. Thus, if the anxiety increases the fitness of women (Alvergne et al. 2010), the polymorphism of Thr136Ile could be maintained by overdominance; however, it is unlikely that the heterozygous advantage occurred in every environment, since Chinese (CDX, CHS, and CHB) and Vietnamese (KHV) populations did not reveal the signature of balancing selection compared to the other non‐African populations (Fig. S2c). This indicates that the genotype effects on the fitness are not spatially or temporally stable and could vary depending on the environment, such as social and/or climatic conditions. When fitness varies spatially and/or temporally and the average heterozygous fitness is larger than those of homozygotes (i.e., emergent overdominance (Delph and Kelly 2014)), variants could be maintained by balancing selection (Levene 1953; Hedrick 2006). The fitness differences among Thr136Ile genotypes might be affected by the seasonal changes due to the latitudinal and/or climatic differences. For example, it is likely that the seasonal depression or seasonal affective disorder, are more prevalent at higher latitudes (Mersch et al. 1999; Yang et al. 2010). Seasonal variation in the binding potential of brain serotonin transporter has been considered to be a key factor contributing to these disorders (Luykx et al. 2013; McMahon et al. 2016). Thus, it is possible that the environmental changes coinciding with the Out‐of‐Africa migration changed the neurological effects of Thr136Ile on one's mind. The influence of various environments on Thr136Ile variants should be examined using larger samples from various populations in future studies.

The Thr136Ile variant in *SLC18A1* affects anxiety, generally associated with personality traits, such as neuroticism. The variant could be found only in humans and has been positively selected during human evolution. Weiss et al. (2012) suggested that balancing selection involved in the generation of human personality traits also has maintained variation in the personality traits of chimpanzees and orangutans. While we share the common genetic substrates underlying personality dimensions with nonhuman apes, quantitative differences might keep us apart. Inoue‐Murayama et al. (2001) reported that the repeat number in the promoter region of 5‐HTT tended to decrease in humans compared to chimpanzees and gorillas, so that greater anxiety was favored in the human ancestors. Therefore, the increased anxiety can be found only in humans, and the Thr136Ile variant in *SLC18A1* could produce human‐unique personality differences in anxiety‐related traits; however, the functions of the Thr136Ile variants of *SLC18A1* remain unclear, so there is a possibility that the variant might affect personality traits other than anxiety.

Although there have been no reports on the effects of the human‐specific amino acid substitutions in the other two genes, *CLSTN2*, which encodes calsyntenin 2, and *FAT1*, which encodes FAT atypical cadherin 1, both belong to the cadherin super family (Gul et al. 2017) and have been surveyed for their roles in neural function. *CLSTN2* is predominantly expressed in the brain (Hintsch 2002) and is involved in learning and memory (Jacobsen et al. 2009; Preuschhof et al. 2010; Lipina et al. 2016), and its function is evolutionarily conserved even in *Caenorhabditis elegans* (Ikeda et al. 2008; Hoerndli et al. 2009). *FAT1* is involved in cell–cell contacts and lamellipodial dynamics (Ciani et al. 2003), and *Fat1*‐deficient mice exhibits defects in forebrain and eye development (Tanoue and Takeichi 2004). Schraut et al. (2014) identified *Fat1* as a gene whose methylation level is affected by interaction with the *5‐Htt* genotype (wild‐type: +/+ and heterozygous: +/−) and prenatal stress exposure in mice. They have also found that prenatal stress exposure increases the methylation level of *Clstn2* and decreases its expression level. The relatively rapid pace of reproduction and improved survival rates resulting from the cooperative breeding are considered to have led to the demographic success of humans (Kramer 2010). Thus, as cooperative breeding evolved, changes in the maternal stress environment and/or anxiety could have caused positive selection for these two genes. Although we also found a significant signature of balancing selection acting on intronic variants in *CLSTN2*, it is unclear how these variants could affect the human brain function.

The present study detected three PD genes that evolved during human evolution by positive natural selection. Among these genes, we have identified the polymorphism at the 136th amino acid site (Thr136Ile) of the *SLC18A1* gene as a human‐specific variant maintained by balancing selection. This gene could have altered the monoamine circuits in the human brain and might influence anxiety‐related personality traits, such as neuroticism. Thus, the Thr136Ile variant of the *SLC18A1* gene might contribute to the quantitative differences of anxiety as a human‐unique personality trait. The present study still has a limitation in describing how such genetic changes affected the evolution of the human brain. Thus, in vitro and/or in vivo experiments using genome editing technology in model animals are needed to further clarify the neurological function of these genes.

Associate Editor: L. Bromham

## Supporting information


**Figure S1**. Comparison of DoS statistic and its constituents, *P*_N_/(*P*_S_+*P*_N_) and *D*_N_/(*D*_S_+*D*_N_) among categories of genes.
**Figure S2a**. The distributions of Tajima's *D* around *FAT1* for each population.
**Figure S2b**. The distributions of Tajima's *D* around *CLSTN2* for each population.
**Figure S2c**. The distributions of Tajima's *D* around *SLC18A1* for each population.
**Figure S3a**. Linkage disequilibrium (LD) in human *CLSTN2* for four populations, YRI, CEU, JPT, and CLM.
**Figure S3b**. Linkage disequilibrium (LD) in human *SLC18A1* for four populations, YRI, CEU, JPT, and CLM.
**Figure S4**. Sequence alignment of *SLC18A1* among 15 mammal species.
**Figure S5**. The distribution of unstandardized nSLs of SNPs with the same allele frequencies as Thr136Ile.
**Figure S6**. The distribution of unstandardized nSL calculated from simulated polymorphic data in each population.
**Figure S7**. The distribution of Tajima's *D* calculated from simulated polymorphic data in each population.
**Figure S8**. Median‐joining haplotype network for *SLC18A1*.
**Figure S9**. Gene tree for *SLC18A1* and coalescent time estimation of 136Ile.
**Figure S10**. A brief description of human VMAT1 predicted by previous studies (Parsons 2000; Wimalasena 2010).

Supporting information

Supporting information

Supporting information

Supporting information

Supporting information

Supporting information

Supporting information

Supporting information

Supporting information

Supporting information

Supporting information

Supporting information

Supporting information

**Table S1**. Psychiatric disorders‐relevant (PD) genes used in the present study.
**Table S2**. Parameters used in coalescent simulations. Maximum likelihood values estimated from a previous study (Gravel et al., 2011) were applied to ms simulator.
**Table S3**. The estimated impact of amino acid substitutions occurring in the human lineage for positively selected genes related to psychiatric disorders (PD‐PSGs).
**Table S4**. The results of the McDonald–Kreitman test for the three positively selected genes related to psychiatric disorders (PD‐PSGs). *P*‐values are calculated by Fisher's exact test.
