# Supplementary material for: Positive and balancing selection on SLC18A1 gene associated with psychiatric disorders and human‐unique personality traits
Source: Evol Lett. 2018 Aug 21;2(5):499–510. doi: 10.1002/evl3.81 (PMC6145502; doi:10.1002/evl3.81)
Supplement: Supplementary file 1 — Figure S1. Comparison of DoS statistic and its constituents, P N/(P S+P N) and D N/(D S+D N) among categories of genes. Figure S2a. The distributions of Tajima's D around FAT1 for each population. Figure S2b. The distributions of Tajima's D around CLSTN2 for each population. Figure S2c. The distributions of Tajima's D around SLC18A1 for each population. Figure S3a. Linkage disequilibrium (LD) in human CLSTN2 for four populations, YRI, CEU, JPT, and CLM. Figure S3b. Linkage disequilibrium (LD) in human SLC18A1 for four populations, YRI, CEU, JPT, and CLM. Figure S4. Sequence alignment of SLC18A1 among 15 mammal species. Figure S5. The distribution of unstandardized nSLs of SNPs with the same allele frequencies as Thr136Ile. Figure S6. The distribution of unstandardized nSL calculated from simulated polymorphic data in each population. Figure S7. The distribution of Tajima's D calculated from simulated polymorphic data in each population. Figure S8. Median‐joining haplotype network for SLC18A1. Figure S9. Gene tree for SLC18A1 and coalescent time estimation of 136Ile. Figure S10. A brief description of human VMAT1 predicted by previous studies (Parsons 2000; Wimalasena 2010). [file EVL3-2-499-s001.docx]

**Figure S1**. **Comparison of DoS statistic and its constituents, *P*_N_/(*P*_S_+*P*_N_) and *D*_N_/(*D*_S_+*D*_N_) among categories of genes.** Plots and bars represent the mean and standard error, respectively. Statistical testing was performed using a generalized linear model (a binomial distribution for *P*_N_ / (*P*_S_ + *P*_N_) and *D*_N_ / (*D*_S_ + *D*_N_) and a gaussian distribution for DoS). Genes were excluded when the denominator equaled 0 (no SNP information in the 1000G dataset for *P*_N_ / (*P*_S_ + *P*_N_) or no interspecific substitutions between humans vs. chimpanzee for *D*_N_ / (*D*_S_ + *D*_N_).

**Figure S2a**. **The distributions of Tajima’s *D* around *FAT1* for each population.** The region of the gene is shown as a gray shadow. The bars above the graph represent exons. The red (horizontal) dotted line indicates the upper thresholds that were determined by the empirical distribution of Tajima’s *D* across the whole genome.

**Figure S2b**. **The distributions of Tajima’s *D* around *CLSTN2* for each population.** The region of the gene is shown as a gray shadow. The bars above the graph represent exons. The red (horizontal) dotted line indicates the upper thresholds that were determined by the empirical distribution of Tajima’s *D* across the whole genome. The regions corresponding to the peaks of Tajima’s *D* observed in non-African populations are highlighted by red shadows.

**Figure S2c**. **The distributions of Tajima’s *D* around *SLC18A1* for each population.** The region of the gene is shown as a gray shadow. The bars above the graph represent exons. The red (horizontal) dotted line indicates the upper thresholds that were determined by (1) the empirical distribution of Tajima’s *D* across the whole genome and (2) the distribution of Tajima’s *D* calculated from the simulated polymorphic data. The region corresponding to the peak of Tajima’s *D* observed in non-African populations is highlighted by a red shadow.

**Figure S3a. Linkage disequilibrium (LD) in human *CLSTN2* for four populations, YRI, CEU, JPT, and CLM.** The number and rectangle above the LD plot represents the position of the corresponding exons. The color of the plot corresponds to the strength of LD between sites, with the red color representing strong LD and the light purple or white colors representing weak or not LD, respectively. The triangle with a black bold line denotes LD blocks.

**Figure S3b. Linkage disequilibrium (LD) in human *SLC18A1* for four populations, YRI, CEU, JPT, and CLM.** The number and rectangle above the LD plot represents the position of the corresponding exons. The color of the plot corresponds to the strength of LD between sites, with the red color representing strong LD and the light purple or white colors representing weak or not LD, respectively. The triangle with a black bold line denotes LD blocks. The Thr136Ile site residing in exon 3 is in a central location of the extended LD in non-African populations

**Figure S4**. **Sequence alignment of *SLC18A1* among 15 mammal species.** Arrow heads represent the two human-specific substitutions at the 130^th^ (Glu > Gly) and 136^th^ (Asn > Thr/Ile) sites. The 136Ile is not shown in this figure. The visualization of the alignment was performed by Aliview (Larsson et al. 2014).

**Figure S5**. **The distribution of unstandardized nSLs of SNPs with the same allele frequencies as Thr136Ile.** The vertical bold black and dotted (red) lines indicate the observed nSL values of Thr136Ile and the values of the 5^th^ percentile of the nSL distribution of 10,000 SNPs with the same allele frequencies as Thr136Ile, respectively.

**Figure S6**. **The distribution of unstandardized nSL calculated from simulated polymorphic data in each population.** nSL was calculated from the polymorphic data of the accepted simulations, which generated the 136Ile allele frequencies that approximated (±5%) those observed in all the three populations. The black bold lines indicate the nSL values calculated from the observed polymorphic data around Thr136Ile polymorphism.

**Figure S7**. **The distribution of Tajima’s *D* calculated from simulated polymorphic data in each population.** Tajima’s *D* was calculated from the polymorphic data of the accepted simulations, which generated the 136Ile allele frequencies that approximated (±5%) those observed in all the three populations. The black bold and red dotted lines indicate the observed Tajima’s *D* values of Thr136Ile and those of the 95^th^ percentile of the distribution, respectively.

**Figure S8. Median-joining haplotype network for *SLC18A1*.** Allelic information for 29 SNPs including Thr136Ile in a LD block in four populations (YRI: gray, CEU: red, CHB: green and JPT: blue) was used for estimation. Each pie chart represents a haplotype, and the size corresponds to the frequency of the haplotype.

**Figure S9. Gene tree for *SLC18A1* and coalescent time estimation of 136Ile.** The information for 30 haplotypes comprising the aforementioned 29 SNPs in global populations (YRI, CEU, CHB and JPT) were used to estimate the gene genealogy. Black circles refer to each haplotype and numbers on the black circles correspond to the haplotypes described in Fig. S8. Numbers below the gene tree represent the frequency of each haplotype in each population.

**Figure S10. A brief description of human VMAT1 predicted by previous studies** (Parsons 2000; Wimalasena 2010). The first luminal loop domain has been shown to regulate monoamine transport (Sievert & Ruoho 1997; Brunk *et al.* 2006). Human-specific substitutions occurred in this domain on the 130^th^ and 136^th^ amino acid sites, Glu130Gly and Asn136Thr/Ile, respectively.

**References**

Brunk, I., Blex, C., Rachakonda, S., Höltje, M., Winter, S., Pahner, I., *et al.* (2006). The first luminal domain of vesicular monoamine transporters mediates G-protein-dependent regulation of transmitter uptake. *J. Biol. Chem.*, 281, 33373–33385.

Parsons, S.M. (2000). Transport mechanisms in acetylcholine and monoamine storage. *FASEB J.*, 14, 2423–34.

Sievert, M.K. & Ruoho, A.E. (1997). Peptide Mapping of the [125I]Iodoazidoketanserin and [125I]2-N-[(3’-Iodo-4’-azidophenyl)propionyl]tetrabenazine Binding Sites for the Synaptic Vesicle Monoamine Transporter. *J. Biol. Chem.*, 272, 26049–26055.

Wimalasena, K. (2010). Vesicular Monoamine Transporters: Structure-Function, Pharmacology, and Medicinal Chemistry. *Med. Res. Rev.*, 31, 483–519.
