# Supplementary material for: Positive and balancing selection on SLC18A1 gene associated with psychiatric disorders and human‐unique personality traits
Source: Evol Lett. 2018 Aug 21;2(5):499–510. doi: 10.1002/evl3.81 (PMC6145502; doi:10.1002/evl3.81)
Supplement: Supplementary file 15 — Table S1. Psychiatric disorders‐relevant (PD) genes used in the present study. Table S2. Parameters used in coalescent simulations. Maximum likelihood values estimated from a previous study (Gravel et al., 2011) were applied to ms simulator. Table S3. The estimated impact of amino acid substitutions occurring in the human lineage for positively selected genes related to psychiatric disorders (PD‐PSGs). Table S4. The results of the McDonald–Kreitman test for the three positively selected genes related to psychiatric disorders (PD‐PSGs). P‐values are calculated by Fisher's exact test. [file EVL3-2-499-s015.docx]

**Table S1. Psychiatric disorders-relevant (PD) genes used in the present study.**

| **Ensembl ID** | **Gene name** | **# of publication** | **Diseases (PubMeb ID)** |
| --- | --- | --- | --- |
| ENSG00000148584 | *A1CF* | 2 | Schizophrenia (12497613), Schizophrenia (10924404) |
| ENSG00000165029 | *ABCA1* | 9 | Schizophrenia (21820670), Schizophrenia (15645182), Schizophrenia (16984965), Schizophrenia (25749018), Schizophrenia (23124177), Schizophrenia (21839797), Bipolar Disorder (25116795), Schizophrenia (19721717), Depressive disorder (23351565) |
| ENSG00000144476 | *ACKR3* | 7 | Schizophrenia (21784156), Schizophrenia (25464914), Major Depressive Disorder (20493668), Bipolar Disorder (16894394), Schizophrenia (20158934), Attention deficit hyperactivity disorder (22897819), Attention deficit hyperactivity disorder (21499268) |
| ENSG00000068366 | *ACSL4* | 2 | Bipolar Disorder (21184843), Depressive disorder (15108178) |
| ENSG00000164398 | *ACSL6* | 5 | Schizophrenia (17030554), Schizophrenia (16827919), Schizophrenia (18718982), Schizophrenia (18804346), Schizophrenia (22205969) |
| ENSG00000087116 | *ADAMTS2* | 3 | Depressive disorder (15627763), Depressive disorder (21905099), Attention deficit hyperactivity disorder (8755795) |
| ENSG00000197381 | *ADARB1* | 5 | Schizophrenia (24443933), Bipolar Disorder (16733555), Schizophrenia (21984433), Bipolar Disorder (15183604), Major Depressive Disorder (22404657) |
| ENSG00000173175 | *ADCY5* | 2 | Unipolar Depression (19846118), Depressive disorder (23278386) |
| ENSG00000121281 | *ADCY7* | 3 | Schizophrenia (21822266), Depressive disorder (22264442), Depressive disorder (17135423) |
| ENSG00000155897 | *ADCY8* | 3 | Depressive disorder (23278386), Bipolar Disorder (18163389), Bipolar Disorder (19691954) |
| ENSG00000078549 | *ADCYAP1R1* | 5 | Bipolar Disorder (19958095), Depressive disorder (23280952), Schizophrenia (27383213), Major Depressive Disorder (26334183), Schizophrenia (17387318) |
| ENSG00000075340 | *ADD2* | 4 | Attention Deficit Disorder (3479015), Attention Deficit Disorder (2890294), Attention Deficit Disorder (3479017), Attention Deficit Disorder (3479016) |
| ENSG00000150471 | *ADGRL3* | 16 | Attention deficit hyperactivity disorder (22832519), Attention deficit hyperactivity disorder (22486528), Attention deficit hyperactivity disorder (20157310), Attention deficit hyperactivity disorder (25989180), Attention deficit hyperactivity disorder (25871512), Attention deficit hyperactivity disorder (21040458), Attention deficit hyperactivity disorder (21184580), Attention deficit hyperactivity disorder (21432600), Attention deficit hyperactivity disorder (25229170), Attention deficit hyperactivity disorder (23190410), Attention deficit hyperactivity disorder (22939004), Attention deficit hyperactivity disorder (23245769), Attention deficit hyperactivity disorder (21606926), Attention deficit hyperactivity disorder (22851411), Attention deficit hyperactivity disorder (22405201), Attention deficit hyperactivity disorder (26746237) |
| ENSG00000181092 | *ADIPOQ* | 3 | Depressive disorder (25597795), Schizophrenia (19193342), Schizophrenia (25284335) |
| ENSG00000148926 | *ADM* | 4 | Bipolar Disorder (23064081), Bipolar Disorder (20713499), Schizophrenia (15194870), Schizophrenia (18081029) |
| ENSG00000101126 | *ADNP* | 4 | Autism Spectrum Disorders (25646590), Schizophrenia (24365867), Autism Spectrum Disorders (24531329), Schizophrenia (20598862) |
| ENSG00000101544 | *ADNP2* | 2 | Schizophrenia (24365867), Schizophrenia (20598862) |
| ENSG00000163485 | *ADORA1* | 2 | Schizophrenia (19820430), Bipolar Disorder (9514582) |
| ENSG00000120907 | *ADRA1A* | 16 | Attention deficit hyperactivity disorder (11326305), Schizophrenia (10940760), Schizophrenia (19918262), Schizophrenia (16043131), Schizophrenia (21784156), Attention deficit hyperactivity disorder (19352218), Schizophrenia (21302347), Schizophrenia (17408692), Bipolar Disorder (16894394), Schizophrenia (22037178), Major Depressive Disorder (20493668), Attention deficit hyperactivity disorder (18821565), Major Depressive Disorder (19095219), Attention deficit hyperactivity disorder (21499268), Schizophrenia (20158934), Attention deficit hyperactivity disorder (22897819) |
| ENSG00000035687 | *ADSS* | 2 | Schizophrenia (18721483), Schizophrenia (19115993) |
| ENSG00000135744 | *AGT* | 8 | Depressive disorder (18628677), Bipolar Disorder (11027844), Depressive disorder (17499413), Depressive disorder (24016839), Depressive disorder (15949885), Attention deficit hyperactivity disorder (18580852), Bipolar Disorder (18363708), Depressive disorder (25131447) |
| ENSG00000135541 | *AHI1* | 11 | Schizophrenia (16773125), Schizophrenia (20371615), Schizophrenia (17473831), Schizophrenia (18785627), Autism Spectrum Disorders (18782849), Depressive disorder (24691070), Bipolar Disorder (20452750), Schizophrenia (20956301), Schizophrenia (20071346), Schizophrenia (20805890), Schizophrenia (25622261) |
| ENSG00000109107 | *ALDOC* | 2 | Schizophrenia (19110265), Schizophrenia (19034380) |
| ENSG00000171094 | *ALK* | 2 | Schizophrenia (16604305), Depressive disorder (17487225) |
| ENSG00000120868 | *APAF1* | 2 | Major Depressive Disorder (19455599), Major Depressive Disorder (16231040) |
| ENSG00000034053 | *APBA2* | 4 | Schizophrenia (17989066), Schizophrenia (19736351), Schizophrenia (19197363), Autism Spectrum Disorders (20029827) |
| ENSG00000110244 | *APOA4* | 3 | Depressive disorder (26491253), Major Depressive Disorder (20580919), Depressive disorder (15806598) |
| ENSG00000084674 | *APOB* | 2 | Bipolar Disorder (24882193), Schizophrenia (20691427) |
| ENSG00000189058 | *APOD* | 5 | Schizophrenia (12942994), Schizophrenia (16966838), Schizophrenia (14623496), Schizophrenia (16402085), Schizophrenia (12363390) |
| ENSG00000198931 | *APRT* | 9 | Schizophrenia (2170638), Schizophrenia (21490598), Major Depressive Disorder (11099740), Depressive disorder (19917299), Depressive disorder (23531112), Depressive disorder (21924326), Depressive disorder (2414305), Schizophrenia (21187413), Depressive disorder (17700644) |
| ENSG00000171885 | *AQP4* | 3 | Schizophrenia (22544010), Schizophrenia (16194264), Depressive disorder (24422972) |
| ENSG00000132694 | *ARHGEF11* | 3 | Schizophrenia (18678470), Schizophrenia (14684465), Schizophrenia (25319871) |
| ENSG00000102606 | *ARHGEF7* | 40 | Schizophrenia (20102668), Schizophrenia (15465979), Schizophrenia (12685995), Schizophrenia (18545269), Schizophrenia (16469942), Schizophrenia (10618013), Schizophrenia (12399955), Schizophrenia (16876141), Schizophrenia (14569275), Schizophrenia (16843094), Schizophrenia (15038993), Schizophrenia (17192894), Schizophrenia (17475740), Schizophrenia (23318559), Schizophrenia (12450949), Schizophrenia (19961902), Schizophrenia (17014995), Schizophrenia (18762587), Schizophrenia (8094267), Schizophrenia (17012698), Schizophrenia (9012828), Schizophrenia (8399828), Bipolar Disorder (16823804), Schizophrenia (20382002), Schizophrenia (15100704), Schizophrenia (18056246), Schizophrenia (17349863), Schizophrenia (8873294), Bipolar Disorder (18189279), Schizophrenia (17448448), Schizophrenia (15625200), Schizophrenia (23910792), Schizophrenia (17604606), Schizophrenia (25464917), Bipolar Disorder (17224092), Schizophrenia (1450287), Schizophrenia (20363872), Schizophrenia (22048129), Schizophrenia (10394470), Schizophrenia (26441157) |
| ENSG00000133794 | *ARNTL* | 15 | Attention deficit hyperactivity disorder (22105622), Bipolar Disorder (24673294), Bipolar Disorder (18228528), Bipolar Disorder (19328558), Bipolar Disorder (20072116), Depressive disorder (19708722), Bipolar Disorder (24636202), Bipolar Disorder (16528748), Bipolar Disorder (19839995), Depressive disorder (20471092), Bipolar Disorder (17239033), Depressive disorder (19693801), Bipolar Disorder (24679394), Depressive disorder (25799324), Bipolar Disorder (26283580) |
| ENSG00000137486 | *ARRB1* | 2 | Major Depressive Disorder (25294870), Unipolar Depression (23011268) |
| ENSG00000100299 | *ARSA* | 2 | Major Depressive Disorder (8897113), Schizophrenia (1975970) |
| ENSG00000099889 | *ARVCF* | 5 | Schizophrenia (20333729), Schizophrenia (22053977), Schizophrenia (15861775), Schizophrenia (19508883), Schizophrenia (24819575) |
| ENSG00000138303 | *ASCC1* | 40 | Schizophrenia (17349863), Bipolar Disorder (18189279), Bipolar Disorder (17224092), Schizophrenia (17192894), Schizophrenia (19961902), Schizophrenia (15465979), Schizophrenia (20382002), Schizophrenia (8873294), Schizophrenia (25464917), Schizophrenia (17604606), Schizophrenia (20363872), Bipolar Disorder (16823804), Schizophrenia (17012698), Schizophrenia (23318559), Schizophrenia (18545269), Schizophrenia (18056246), Schizophrenia (16469942), Schizophrenia (9012828), Schizophrenia (12399955), Schizophrenia (1450287), Schizophrenia (10394470), Schizophrenia (20102668), Schizophrenia (22048129), Schizophrenia (17475740), Schizophrenia (17014995), Schizophrenia (14569275), Schizophrenia (16843094), Schizophrenia (18762587), Bipolar Disorder (15625200), Schizophrenia (8094267), Schizophrenia (26441157), Schizophrenia (12685995), Schizophrenia (15100704), Schizophrenia (8399828), Schizophrenia (15038993), Schizophrenia (10618013), Schizophrenia (12450949), Schizophrenia (17448448), Schizophrenia (23910792), Schizophrenia (16876141) |
| ENSG00000152092 | *ASTN1* | 2 | Schizophrenia (22488871), Schizophrenia (18384059) |
| ENSG00000115966 | *ATF2* | 2 | Schizophrenia (18077426), Depressive disorder (14647483) |
| ENSG00000162772 | *ATF3* | 2 | Schizophrenia (21421043), Depressive disorder (18305237) |
| ENSG00000171681 | *ATF7IP* | 11 | Depressive disorder (24554194), Depressive disorder (17906375), Major Depressive Disorder (26010085), Depressive disorder (18081710), Depressive disorder (19844206), Depressive disorder (16343697), Depressive disorder (20014955), Depressive disorder (15365580), Depressive disorder (25220861), Depressive disorder (8835706), Depressive disorder (19629761) |
| ENSG00000057663 | *ATG5* | 2 | Schizophrenia (10924404), Schizophrenia (12497613) |
| ENSG00000163399 | *ATP1A1* | 2 | Bipolar Disorder (19058785), Bipolar Disorder (9807644) |
| ENSG00000105409 | *ATP1A3* | 4 | Depressive disorder (21418141), Bipolar Disorder (19058785), Bipolar Disorder (11353452), Bipolar Disorder (9646882) |
| ENSG00000174437 | *ATP2A2* | 7 | Depressive disorder (11377748), Schizophrenia (25213221), Schizophrenia (23055483), Bipolar Disorder (11690598), Schizophrenia (27106560), Bipolar Disorder (11244492), Depressive disorder (15169625) |
| ENSG00000157087 | *ATP2B2* | 2 | Schizophrenia (19850283), Autism Spectrum Disorders (21757185) |
| ENSG00000124788 | *ATXN1* | 6 | Schizophrenia (11121205), Bipolar Disorder (16380905), Schizophrenia (9184318), Schizophrenia (10335546), Attention deficit hyperactivity disorder (21302343), Attention deficit hyperactivity disorder (25162476) |
| ENSG00000166148 | *AVPR1A* | 7 | Autism Spectrum Disorders (20546835), Autism Spectrum Disorders (16520824), Schizophrenia (17227684), Bipolar Disorder (16403186), Schizophrenia (25529259), Autism Spectrum Disorders (20452058), Autism Spectrum Disorders (19195791) |
| ENSG00000198049 | *AVPR1B* | 7 | Recurrent major depressive episodes (15094789), Bipolar Disorder (22341483), Bipolar Disorder (26354157), Bipolar Disorder (23962971), Bipolar Disorder (24012103), Depressive disorder (23422793), Attention deficit hyperactivity disorder (19668115) |
| ENSG00000109956 | *B3GAT1* | 3 | Schizophrenia (16815618), Schizophrenia (12874601), Schizophrenia (20950796) |
| ENSG00000186318 | *BACE1* | 3 | Schizophrenia (20926259), Schizophrenia (19536656), Schizophrenia (19828790) |
| ENSG00000114200 | *BCHE* | 3 | Schizophrenia (15585443), Attention deficit hyperactivity disorder (24041656), Bipolar Disorder (4053343) |
| ENSG00000116128 | *BCL9* | 3 | Schizophrenia (21383261), Schizophrenia (23382809), Schizophrenia (26494551) |
| ENSG00000176697 | *BDNF* | 531 | Depressive disorder (15384083), Major Depressive Disorder (18852698), Depressive disorder (25572252), Attention deficit hyperactivity disorder (20943059), Schizophrenia (22845879), Depressive disorder (23570887), Attention deficit hyperactivity disorder (22825876), Bipolar Disorder (16472361), Major Depressive Disorder (25044977), Major Depressive Disorder (23165919), Major Depressive Disorder (26462036), Depressive disorder (15918078), Major Depressive Disorder (26239616), Depressive disorder (24683520), Unipolar Depression (20207421), Bipolar Disorder (17895927), Major Depressive Disorder (19414708), Schizophrenia (17442489), Autism Spectrum Disorders (19582565), Major Depressive Disorder (19931400), Depressive disorder (24035489), Bipolar Disorder (18826446), Depressive disorder (22378223), Schizophrenia (19054571), Major Depressive Disorder (21938001), Bipolar Disorder (24862657), Schizophrenia (18205169), Major Depressive Disorder (25106037), Depressive disorder (20621121), Schizophrenia (18628679), Bipolar Disorder (17417060), Unipolar Depression (19668114), Major Depressive Disorder (18721847), Bipolar Disorder (19328558), Depressive disorder (17680923), Depressive disorder (26118823), Unipolar Depression (22681170), Schizophrenia (16005437), Schizophrenia (22213405), Major Depressive Disorder (17401528), Depressive disorder (19885623), Depressive disorder (22682508), Schizophrenia (15543516), Bipolar Disorder (18242852), Major Depressive Disorder (24213247), Attention deficit hyperactivity disorder (15940292), Major Depressive Disorder (22801293), Schizophrenia (16603474), Unipolar Depression (26921055), Attention deficit hyperactivity disorder (17427194), Major Depressive Disorder (25387785), Major Depressive Disorder (26117067), Depressive disorder (16043130), Major Depressive Disorder (21338649), Major Depressive Disorder (26717541), Schizophrenia (20667458), Unipolar Depression (25510949), Attention deficit hyperactivity disorder (25354496), Unipolar Depression (23250003), Schizophrenia (16897602), Depressive disorder (14625141), Schizophrenia (19336781), Schizophrenia (18583979), Major Depressive Disorder (25012449), Major Depressive Disorder (24972302), Schizophrenia (18472202), Bipolar Disorder (23182421), Schizophrenia (19376528), Schizophrenia (20087404), Schizophrenia (9858028), Depressive disorder (20345896), Depressive disorder (17850220), Bipolar Disorder (12140781), Bipolar Disorder (16025419), Bipolar Disorder (17239400), Depressive disorder (14699963), Depressive disorder (25277075), Depressive disorder (16631126), Bipolar Disorder (21931317), Major Depressive Disorder (19589373), Depressive disorder (22610920), Schizophrenia (17894414), Unipolar Depression (24093582), Unipolar Depression (25745134), Depressive disorder (18602452), Unipolar Depression (23128924), Bipolar Disorder (19953912), Schizophrenia (15630410), Depressive disorder (17920693), Bipolar Disorder (18657242), Major Depressive Disorder (20682308), Major Depressive Disorder (27787964), Major Depressive Disorder (22247094), Schizophrenia (26706021), Schizophrenia (21366724), Schizophrenia (19018714), Major Depressive Disorder (16300747), Schizophrenia (25125238), Unipolar Depression (22094229), Bipolar Disorder (19295510), Depressive disorder (19621014), Unipolar Depression (21912609), Bipolar Disorder (20627389), Bipolar Disorder (16568151), Major Depressive Disorder (23603442), Bipolar Disorder (18606495), Schizophrenia (22576830), Bipolar Disorder (26432032), Bipolar Disorder (19473771), Depressive disorder (17151862), Major Depressive Disorder (22965830), Schizophrenia (25246365), Major Depressive Disorder (20033742), Schizophrenia (19046988), Schizophrenia (17366345), Schizophrenia (21788944), Schizophrenia (20456319), Bipolar Disorder (26528762), Depressive disorder (18450378), Major Depressive Disorder (16979146), Attention deficit hyperactivity disorder (21063237), Bipolar Disorder (15744031), Unipolar Depression (18752720), Depressive disorder (23532066), Depressive disorder (20163778), Unipolar Depression (18179845), Bipolar Disorder (18497095), Depressive disorder (26285129), Bipolar Disorder (25498133), Major Depressive Disorder (20921514), Major Depressive Disorder (18982004), Bipolar Disorder (24468644), Major Depressive Disorder (26010085), Schizophrenia (23973796), Bipolar Disorder (15274036), Schizophrenia (20061032), Depressive disorder (14730194), Depressive disorder (26935651), Bipolar Disorder (20142305), Schizophrenia (16854566), Depressive disorder (26721949), Depressive disorder (18497103), Attention deficit hyperactivity disorder (17918236), Major Depressive Disorder (25658497), Depressive disorder (26892396), Depressive disorder (23481907), Schizophrenia (15949651), Depressive disorder (21464126), Major Depressive Disorder (21129438), Schizophrenia (15768049), Bipolar Disorder (21223646), Schizophrenia (22777684), Depressive disorder (21531985), Schizophrenia (18062171), Depressive disorder (15892658), Schizophrenia (25529856), Major Depressive Disorder (20167454), Bipolar Disorder (22877924), Unipolar Depression (22844024), Schizophrenia (18056245), Depressive disorder (21103954), Depressive disorder (23619509), Schizophrenia (19457211), Depressive disorder (19846118), Depressive disorder (19236730), Major Depressive Disorder (17404118), Attention deficit hyperactivity disorder (19721846), Schizophrenia (17628439), Unipolar Depression (19699537), Depressive disorder (20034565), Attention deficit hyperactivity disorder (18580852), Bipolar Disorder (19578686), Unipolar Depression (24577123), Schizophrenia (23832605), Depressive disorder (18603005), Schizophrenia (18486103), Bipolar Disorder (25539739), Depressive disorder (19215635), Unipolar Depression (20674983), Schizophrenia (17987059), Depressive disorder (17222482), Depressive disorder (17006024), Schizophrenia (25612896), Schizophrenia (15666411), Attention deficit hyperactivity disorder (23972692), Schizophrenia (26215504), Schizophrenia (26851233), Schizophrenia (18856059), Attention deficit hyperactivity disorder (19603419), Schizophrenia (23923080), Attention Deficit Disorder (21466746), Major Depressive Disorder (22194899), Bipolar Disorder (14668941), Schizophrenia (24399714), Depressive disorder (26641254), Bipolar Disorder (23567599), Bipolar Disorder (17284422), Schizophrenia (17217930), Depressive disorder (17959306), Unipolar Depression (25451398), Schizophrenia (18408624), Depressive disorder (21188787), Bipolar Disorder (14708030), Depressive disorder (23733090), Schizophrenia (20100784), Autism Spectrum Disorders (26485544), Major Depressive Disorder (12842310), Schizophrenia (24702539), Schizophrenia (20420877), Major Depressive Disorder (21692988), Schizophrenia (24289908), Schizophrenia (22030467), Depressive disorder (26393465), Bipolar Disorder (21167606), Major Depressive Disorder (22033217), Bipolar Disorder (19167700), Attention deficit hyperactivity disorder (18821565), Bipolar Disorder (16538178), Schizophrenia (16389585), Bipolar Disorder (12161822), Bipolar Disorder (22548711), Depressive disorder (21577214), Major Depressive Disorder (20230085), Depressive disorder (26801497), Depressive disorder (22265241), Depressive disorder (20227453), Schizophrenia (24993285), Depressive disorder (18832862), Schizophrenia (25681004), Depressive disorder (24586790), Bipolar Disorder (16124838), Bipolar Disorder (25364290), Schizophrenia (19714565), Bipolar Disorder (16741941), Schizophrenia (14623369), Depressive disorder (16343697), Depressive disorder (26070769), Schizophrenia (16513879), Major Depressive Disorder (24927694), Depressive disorder (23880378), Attention deficit hyperactivity disorder (19270759), Depressive disorder (20478625), Schizophrenia (26134309), Major Depressive Disorder (14582140), Bipolar Disorder (17143075), Major Depressive Disorder (21912391), Schizophrenia (16406671), Schizophrenia (20430595), Depressive disorder (21945287), Depressive disorder (22503724), Attention deficit hyperactivity disorder (25061595), Unipolar Depression (21218562), Depressive disorder (23704927), Schizophrenia (18454098), Schizophrenia (23319002), Bipolar Disorder (14673216), Schizophrenia (24935406), Bipolar Disorder (17012654), Schizophrenia (26889735), Schizophrenia (19207030), Depressive disorder (21143020), Depressive disorder (20017110), Schizophrenia (19359449), Major Depressive Disorder (23570888), Schizophrenia (18596619), Bipolar Disorder (14636373), Major Depressive Disorder (18081157), Depressive disorder (22014446), Depressive disorder (22883353), Unipolar Depression (23255668), Major Depressive Disorder (26876488), Depressive disorder (23992681), Schizophrenia (25874530), Schizophrenia (25025909), Depressive disorder (21898033), Schizophrenia (11032392), Unipolar Depression (21960735), Depressive disorder (20226536), Schizophrenia (20638435), Depressive disorder (21997575), Major Depressive Disorder (22504456), Schizophrenia (18325670), Bipolar Disorder (19193231), Schizophrenia (20951727), Schizophrenia (21688113), Schizophrenia (16581172), Major Depressive Disorder (20653908), Major Depressive Disorder (25796564), Schizophrenia (17720314), Bipolar Disorder (22517381), Depressive disorder (22210241), Attention deficit hyperactivity disorder (21733227), Unipolar Depression (22585743), Schizophrenia (23487199), Major Depressive Disorder (23341118), Schizophrenia (21712773), Attention deficit hyperactivity disorder (15457498), Attention deficit hyperactivity disorder (18286632), Schizophrenia (22477643), Schizophrenia (15526143), Schizophrenia (26700405), Depressive disorder (23063133), Bipolar Disorder (19018715), Depressive disorder (23684538), Bipolar Disorder (16388065), Depressive disorder (19335934), Schizophrenia (16533563), Bipolar Disorder (18313696), Depressive disorder (23303061), Attention deficit hyperactivity disorder (16996699), Depressive disorder (26313133), Depressive disorder (24655651), Depressive disorder (18548532), Schizophrenia (23438165), Depressive disorder (16330021), Bipolar Disorder (18843195), Bipolar Disorder (19330778), Bipolar Disorder (14735135), Schizophrenia (18602732), Schizophrenia (19406621), Schizophrenia (16741916), Schizophrenia (21044653), Schizophrenia (24069289), Depressive disorder (21880305), Schizophrenia (23157625), Bipolar Disorder (18664999), Bipolar Disorder (25476119), Depressive disorder (23904143), Depressive disorder (20921572), Attention deficit hyperactivity disorder (19352218), Schizophrenia (25264289), Unipolar Depression (23968401), Depressive disorder (17911362), Major Depressive Disorder (23184535), Depressive disorder (26756166), Schizophrenia (17012697), Schizophrenia (19530966), Schizophrenia (16056149), Unipolar Depression (26556688), Schizophrenia (16818862), Major Depressive Disorder (20703451), Schizophrenia (24595507), Depressive disorder (17579366), Schizophrenia (23532065), Major Depressive Disorder (21510922), Schizophrenia (15647480), Bipolar Disorder (15337662), Major Depressive Disorder (20195291), Bipolar Disorder (19018231), Depressive disorder (19423950), Schizophrenia (12851636), Bipolar Disorder (15336520), Depressive disorder (25422957), Depressive disorder (17409734), Schizophrenia (21710362), Schizophrenia (15940304), Schizophrenia (17869486), Depressive disorder (18990498), Schizophrenia (18497099), Major Depressive Disorder (18781861), Depressive disorder (22097208), Autism Spectrum Disorders (26728085), Autism Spectrum Disorders (25535174), Depressive disorder (23269207), Depressive disorder (22652301), Depressive disorder (22240101), Depressive disorder (21996278), Bipolar Disorder (16152572), Schizophrenia (10581496), Bipolar Disorder (22085476), Schizophrenia (20553817), Depressive disorder (20728877), Depressive disorder (20097294), Bipolar Disorder (25041243), Major Depressive Disorder (17036259), Bipolar Disorder (23315174), Depressive disorder (20016225), Depressive disorder (18842305), Attention deficit hyperactivity disorder (24713358), Schizophrenia (26503495), Depressive disorder (24338983), Bipolar Disorder (18704093), Depressive disorder (24801253), Schizophrenia (24556472), Schizophrenia (15626824), Depressive disorder (26795846), Depressive disorder (19179848), Bipolar Disorder (18335055), Depressive disorder (17472225), Schizophrenia (24551075), Schizophrenia (11343865), Major Depressive Disorder (25990886), Bipolar Disorder (22514151), Depressive disorder (26556286), Attention deficit hyperactivity disorder (18428117), Depressive disorder (24433458), Depressive disorder (19473709), Depressive disorder (18208542), Autism Spectrum Disorders (23517654), Schizophrenia (20708907), Major Depressive Disorder (25568448), Attention deficit hyperactivity disorder (17219016), Schizophrenia (23433505), Attention deficit hyperactivity disorder (17044097), Schizophrenia (19121517), Depressive disorder (23399480), Schizophrenia (18253057), Depressive disorder (16890377), Depressive disorder (19339052), Major Depressive Disorder (19095219), Depressive disorder (17726474), Bipolar Disorder (23253673), Depressive disorder (18347599), Depressive disorder (19661618), Major Depressive Disorder (22609366), Depressive disorder (21677379), Depressive disorder (21037213), Depressive disorder (18600033), Bipolar Disorder (20172611), Depressive disorder (22931410), Depressive disorder (25283187), Schizophrenia (17289348), Unipolar Depression (17632285), Bipolar Disorder (21098877), Depressive disorder (15940299), Depressive disorder (25648279), Depressive disorder (24070857), Schizophrenia (17413445), Depressive disorder (23848344), Unipolar Depression (26078197), Bipolar Disorder (20483468), Schizophrenia (25455509), Depressive disorder (21056046), Schizophrenia (12921913), Schizophrenia (12837526), Bipolar Disorder (19330793), Schizophrenia (18187310), Depressive disorder (24745471), Bipolar Disorder (16787706), Bipolar Disorder (24398043), Major Depressive Disorder (21889574), Attention deficit hyperactivity disorder (20691787), Unipolar Depression (19493959), Schizophrenia (15567073), Depressive disorder (18263664), Attention deficit hyperactivity disorder (25425456), Depressive disorder (25347540), Schizophrenia (19944766), Unipolar Depression (20014955), Schizophrenia (20957650), Depressive disorder (24128869), Depressive disorder (25324022), Depressive disorder (20220593), Major Depressive Disorder (23137999), Schizophrenia (19344762), Bipolar Disorder (18787660), Major Depressive Disorder (18329817), Depressive disorder (25618300), Depressive disorder (17700574), Autism Spectrum Disorders (25669997), Attention deficit hyperactivity disorder (17216343), Schizophrenia (16631352), Schizophrenia (25751398), Depressive disorder (25838322), Depressive disorder (16797081), Schizophrenia (17267117), Depressive disorder (22134958), Schizophrenia (17196936), Depressive disorder (19548263), Autism Spectrum Disorders (24781735), Depressive disorder (21215389), Depressive disorder (20923424), Schizophrenia (21795612), Depressive disorder (25998702), Depressive disorder (26000753), Schizophrenia (26603468), Depressive disorder (20553751), Bipolar Disorder (24021960), Attention deficit hyperactivity disorder (16919891), Depressive disorder (24608543), Schizophrenia (22954755), Depressive disorder (20667416), Schizophrenia (20691427), Bipolar Disorder (15948763), Schizophrenia (18535997), Depressive disorder (15913870), Schizophrenia (12951204), Schizophrenia (23065263), Bipolar Disorder (12524161), Depressive disorder (16458264), Schizophrenia (24713399), Schizophrenia (23739121), Depressive disorder (20346518), Major Depressive Disorder (18923405), Bipolar Disorder (23957567), Bipolar Disorder (17885603), Schizophrenia (17604122), Schizophrenia (22521161), Depressive disorder (19111910), Schizophrenia (15952869), Depressive disorder (17392738), Depressive disorder (19153574), Depressive disorder (21420735), Bipolar Disorder (23026378), Attention deficit hyperactivity disorder (25950609), Bipolar Disorder (26621529), Schizophrenia (22362486), Depressive disorder (20515664), Depressive disorder (17482146), Schizophrenia (25562189), Depressive disorder (24722042), Depressive disorder (16606648), Schizophrenia (23938174), Major Depressive Disorder (20124106), Schizophrenia (16386272), Depressive disorder (17941097), Bipolar Disorder (22484201), Depressive disorder (24589356), Schizophrenia (21641949), Bipolar Disorder (22353757), Depressive disorder (22044630), Bipolar Disorder (14609502), Depressive disorder (20515362), Major Depressive Disorder (17510948), Schizophrenia (21248641), Bipolar Disorder (24308275), Schizophrenia (25219617), Attention deficit hyperactivity disorder (23109089), Bipolar Disorder (27221213), Schizophrenia (21917241), Schizophrenia (15719396) |
| ENSG00000126581 | *BECN1* | 2 | Schizophrenia (24365867), Depressive disorder (25386878) |
| ENSG00000122870 | *BICC1* | 4 | Depressive disorder (22910460), Depressive disorder (20516156), Major Depressive Disorder (26391493), Major Depressive Disorder (25178406) |
| ENSG00000101144 | *BMP7* | 2 | Major Depressive Disorder (21896235), Major Depressive Disorder (19846067) |
| ENSG00000089234 | *BRAP* | 2 | Schizophrenia (24454952), Bipolar Disorder (9322233) |
| ENSG00000102239 | *BRS3* | 6 | Attention deficit hyperactivity disorder (22897819), Bipolar Disorder (16894394), Schizophrenia (20158934), Attention deficit hyperactivity disorder (21499268), Major Depressive Disorder (20493668), Schizophrenia (21784156) |
| ENSG00000164061 | *BSN* | 3 | Schizophrenia (19367581), Schizophrenia (19165527), Bipolar Disorder (19567891) |
| ENSG00000147894 | *C9orf72* | 8 | Schizophrenia (26723138), Schizophrenia (24387986), Schizophrenia (25115936), Schizophrenia (23036583), Schizophrenia (25433797), Bipolar Disorder (23551834), Schizophrenia (26691640), Schizophrenia (26862832) |
| ENSG00000104267 | *CA2* | 3 | Schizophrenia (18163426), Bipolar Disorder (23140658), Schizophrenia (19034380) |
| ENSG00000141837 | *CACNA1A* | 3 | Schizophrenia (10412193), Autism Spectrum Disorders (26566276), Bipolar Disorder (19819557) |
| ENSG00000151067 | *CACNA1C* | 77 | Bipolar Disorder (26401721), Bipolar Disorder (24581832), Schizophrenia (20819988), Schizophrenia (24461634), Schizophrenia (19065143), Schizophrenia (26475575), Unipolar Depression (21078228), Schizophrenia (26276307), Unipolar Depression (24612926), Bipolar Disorder (22488967), Schizophrenia (24262814), Bipolar Disorder (21767209), Depressive disorder (24643163), Schizophrenia (24642287), Bipolar Disorder (26227746), Bipolar Disorder (23406546), Bipolar Disorder (25290268), Bipolar Disorder (25976633), Schizophrenia (23900723), Bipolar Disorder (21837483), Bipolar Disorder (22012475), Bipolar Disorder (21057379), Bipolar Disorder (20679588), Schizophrenia (23404764), Bipolar Disorder (20636642), Bipolar Disorder (21395576), Depressive disorder (22705413), Bipolar Disorder (24108394), Bipolar Disorder (23880959), Bipolar Disorder (21676128), Bipolar Disorder (23568192), Schizophrenia (21037240), Bipolar Disorder (24411473), Bipolar Disorder (18711365), Major Depressive Disorder (19781653), Bipolar Disorder (19621016), Bipolar Disorder (20638048), Bipolar Disorder (23070075), Bipolar Disorder (21926972), Depressive disorder (20226536), Schizophrenia (23437284), Major Depressive Disorder (21042317), Bipolar Disorder (19388002), Bipolar Disorder (25304227), Schizophrenia (26048451), Bipolar Disorder (22464935), Schizophrenia (19521722), Autism Spectrum Disorders (21624971), Bipolar Disorder (26476274), Bipolar Disorder (23437964), Bipolar Disorder (26541689), Autism Spectrum Disorders (25633834), Schizophrenia (25588813), Schizophrenia (23860750), Bipolar Disorder (20723887), Schizophrenia (26049408), Bipolar Disorder (27221213), Bipolar Disorder (24716743), Bipolar Disorder (21292451), Bipolar Disorder (21926974), Schizophrenia (25470093), Schizophrenia (20600464), Schizophrenia (26255836), Major Depressive Disorder (23680436), Bipolar Disorder (19329560), Schizophrenia (22665259), Unipolar Depression (22647524), Bipolar Disorder (20886543), Bipolar Disorder (19819557), Autism Spectrum Disorders (24752249), Schizophrenia (24275578), Bipolar Disorder (22628130), Schizophrenia (22614287), Bipolar Disorder (23979604), Schizophrenia (26204268), Schizophrenia (24355530), Schizophrenia (22957138) |
| ENSG00000157388 | *CACNA1D* | 3 | Schizophrenia (26255836), Autism Spectrum Disorders (25620733), Bipolar Disorder (26476274) |
| ENSG00000165995 | *CACNB2* | 3 | Schizophrenia (24901509), Bipolar Disorder (24581832), Autism Spectrum Disorders (24752249) |
| ENSG00000166862 | *CACNG2* | 4 | Schizophrenia (18571626), Schizophrenia (17826036), Schizophrenia (18408563), Attention Deficit Disorder (23555897) |
| ENSG00000084774 | *CAD* | 2 | Depressive disorder (16554382), Schizophrenia (20659789) |
| ENSG00000182985 | *CADM1* | 3 | Depressive disorder (22113448), Autism Spectrum Disorders (22272290), Autism Spectrum Disorders (21364653) |
| ENSG00000081803 | *CADPS2* | 2 | Schizophrenia (21601610), Autism Spectrum Disorders (24737869) |
| ENSG00000104327 | *CALB1* | 2 | Schizophrenia (24674775), Schizophrenia (18585682) |
| ENSG00000172137 | *CALB2* | 3 | Major Depressive Disorder (21226980), Schizophrenia (26337038), Schizophrenia (12867516) |
| ENSG00000130643 | *CALY* | 5 | Attention deficit hyperactivity disorder (19352218), Attention deficit hyperactivity disorder (21403673), Schizophrenia (15364041), Schizophrenia (14755439), Attention deficit hyperactivity disorder (16172615) |
| ENSG00000070808 | *CAMK2A* | 4 | Schizophrenia (17603807), Unipolar Depression (18068248), Depressive disorder (21611732), Bipolar Disorder (19328558) |
| ENSG00000110931 | *CAMKK2* | 4 | Schizophrenia (23958956), Schizophrenia (26354101), Schizophrenia (26395653), Bipolar Disorder (16673375) |
| ENSG00000164326 | *CARTPT* | 6 | Depressive disorder (23697793), Depressive disorder (19254763), Schizophrenia (20362026), Depressive disorder (18096245), Depressive disorder (16400624), Bipolar Disorder (15234472) |
| ENSG00000147044 | *CASK* | 3 | Schizophrenia (19736351), Autism Spectrum Disorders (26575289), Autism Spectrum Disorders (24505460) |
| ENSG00000163394 | *CCKAR* | 12 | Schizophrenia (17413452), Schizophrenia (22825913), Schizophrenia (19753663), Major Depressive Disorder (15927089), Schizophrenia (15363473), Schizophrenia (10572328), Bipolar Disorder (21978736), Schizophrenia (17413443), Schizophrenia (11549403), Schizophrenia (10893485), Schizophrenia (18583979), Schizophrenia (10889525) |
| ENSG00000004468 | *CD38* | 7 | Autism Spectrum Disorders (22366648), Autism Spectrum Disorders (27015428), Autism Spectrum Disorders (22227279), Autism Spectrum Disorders (21557943), Autism Spectrum Disorders (21182206), Autism Spectrum Disorders (21528155), Autism Spectrum Disorders (24634087) |
| ENSG00000117091 | *CD48* | 2 | Schizophrenia (22495247), Schizophrenia (18617371) |
| ENSG00000158985 | *CDC42SE2* | 3 | Schizophrenia (17030554), Schizophrenia (22205969), Schizophrenia (18804346) |
| ENSG00000126759 | *CFP* | 3 | Schizophrenia (21955727), Major Depressive Disorder (25734512), Schizophrenia (19566714) |
| ENSG00000070748 | *CHAT* | 5 | Depressive disorder (18603262), Depressive disorder (21507424), Schizophrenia (17503482), Schizophrenia (16023618), Schizophrenia (10594838) |
| ENSG00000100888 | *CHD8* | 11 | Autism Spectrum Disorders (23285124), Autism Spectrum Disorders (23160955), Autism Spectrum Disorders (25752243), Autism Spectrum Disorders (26668231), Autism Spectrum Disorders (26834018), Autism Spectrum Disorders (26789910), Autism Spectrum Disorders (25257502), Autism Spectrum Disorders (24998929), Autism Spectrum Disorders (25989142), Autism Spectrum Disorders (26921529), Autism Spectrum Disorders (25294932) |
| ENSG00000089199 | *CHGB* | 3 | Schizophrenia (11959426), Schizophrenia (15219467), Schizophrenia (17143778) |
| ENSG00000134121 | *CHL1* | 9 | Schizophrenia (15653271), Schizophrenia (21332311), Schizophrenia (11033343), Depressive disorder (25943212), Schizophrenia (11986985), Schizophrenia (23857787), Schizophrenia (11681838), Schizophrenia (9098580), Schizophrenia (10696830) |
| ENSG00000187446 | *CHP1* | 2 | Schizophrenia (19165527), Major Depressive Disorder (10321984) |
| ENSG00000111666 | *CHPT1* | 8 | Attention deficit hyperactivity disorder (18083349), Schizophrenia (11295379), Attention deficit hyperactivity disorder (22564179), Schizophrenia (11871372), Schizophrenia (17631280), Schizophrenia (26494551), Schizophrenia (17630256), Schizophrenia (11087021) |
| ENSG00000133019 | *CHRM3* | 2 | Bipolar Disorder (23962466), Schizophrenia (26959877) |
| ENSG00000184984 | *CHRM5* | 2 | Schizophrenia (19944766), Schizophrenia (15292665) |
| ENSG00000120903 | *CHRNA2* | 2 | Bipolar Disorder (15996750), Depressive disorder (19204725) |
| ENSG00000147434 | *CHRNA6* | 3 | Bipolar Disorder (21191315), Depressive disorder (19204725), Attention deficit hyperactivity disorder (23899432) |
| ENSG00000147432 | *CHRNB3* | 4 | Bipolar Disorder (24675634), Bipolar Disorder (21191315), Attention deficit hyperactivity disorder (23899432), Bipolar Disorder (19204725) |
| ENSG00000122966 | *CIT* | 6 | Major Depressive Disorder (20859246), Depressive disorder (12478878), Bipolar Disorder (15983625), Schizophrenia (20084519), Bipolar Disorder (19328558), Major Depressive Disorder (18940259) |
| ENSG00000134852 | *CLOCK* | 51 | Major Depressive Disorder (19347611), Bipolar Disorder (14582141), Schizophrenia (21882241), Schizophrenia (20364331), Depressive disorder (22535308), Depressive disorder (22826347), Depressive disorder (20174623), Bipolar Disorder (24679394), Unipolar Depression (10700653), Bipolar Disorder (19909672), Attention deficit hyperactivity disorder (24068320), Major Depressive Disorder (24001941), Unipolar Depression (25349171), Bipolar Disorder (10904433), Schizophrenia (25135782), Depressive disorder (18487629), Major Depressive Disorder (24581835), Bipolar Disorder (24150227), Bipolar Disorder (21781277), Bipolar Disorder (24865659), Major Depressive Disorder (23273899), Unipolar Depression (26926884), Major Depressive Disorder (22080789), Depressive disorder (24673294), Bipolar Disorder (25724486), Depressive disorder (25522426), Bipolar Disorder (18228528), Bipolar Disorder (18247375), Schizophrenia (17116390), Depressive disorder (23516567), Bipolar Disorder (20856823), Depressive disorder (17917561), Unipolar Depression (10898925), Bipolar Disorder (18419323), Schizophrenia (19839995), Bipolar Disorder (12898572), Major Depressive Disorder (17969870), Schizophrenia (19224106), Schizophrenia (11511917), Bipolar Disorder (26531027), Bipolar Disorder (19909500), Bipolar Disorder (18789374), Bipolar Disorder (21316201), Bipolar Disorder (15722957), Depressive disorder (19926609), Bipolar Disorder (20072116), Depressive disorder (20663803), Major Depressive Disorder (15475734), Bipolar Disorder (22384149), Bipolar Disorder (19328558), Schizophrenia (21050724) |
| ENSG00000158258 | *CLSTN2* | 2 | Schizophrenia (20691427), Unipolar Depression (25080189) |
| ENSG00000164309 | *CMYA5* | 3 | Schizophrenia (23778016), Major Depressive Disorder (24988482), Schizophrenia (20838396) |
| ENSG00000173786 | *CNP* | 16 | Major Depressive Disorder (22832658), Schizophrenia (16891421), Schizophrenia (16389193), Schizophrenia (19034380), Major Depressive Disorder (25930075), Schizophrenia (17964117), Schizophrenia (17306456), Schizophrenia (17010574), Schizophrenia (24788877), Schizophrenia (23032943), Schizophrenia (19165527), Schizophrenia (19348671), Schizophrenia (18289148), Schizophrenia (17223013), Schizophrenia (18496213), Depressive disorder (22473874) |
| ENSG00000188822 | *CNR2* | 11 | Depressive disorder (17105950), Depressive disorder (18286196), Schizophrenia (25014618), Depressive disorder (24467609), Depressive disorder (18991891), Depressive disorder (20649579), Schizophrenia (21430651), Bipolar Disorder (21658778), Depressive disorder (22826533), Schizophrenia (23846977), Schizophrenia (19931854) |
| ENSG00000144619 | *CNTN4* | 6 | Schizophrenia (19736351), Autism Spectrum Disorders (19404257), Autism Spectrum Disorders (18179895), Bipolar Disorder (22205951), Autism Spectrum Disorders (18349135), Autism Spectrum Disorders (21308999) |
| ENSG00000145920 | *CPLX2* | 13 | Schizophrenia (20412316), Schizophrenia (15653259), Schizophrenia (20819981), Schizophrenia (16442780), Schizophrenia (25297695), Schizophrenia (11483314), Schizophrenia (25564026), Bipolar Disorder (14708030), Depressive disorder (20584925), Schizophrenia (16131404), Schizophrenia (9853440), Schizophrenia (21145444), Attention deficit hyperactivity disorder (21832240) |
| ENSG00000118260 | *CREB1* | 43 | Unipolar Depression (20643483), Recurrent major depressive episodes (14582139), Schizophrenia (22198373), Major Depressive Disorder (17300755), Unipolar Depression (17548750), Major Depressive Disorder (24006268), Schizophrenia (25043418), Bipolar Disorder (18189280), Major Depressive Disorder (19194961), Schizophrenia (10570922), Unipolar Depression (25059218), Major Depressive Disorder (21598377), Major Depressive Disorder (23568192), Major Depressive Disorder (23844928), Major Depressive Disorder (18213625), Depressive disorder (20943350), Major Depressive Disorder (18678793), Depressive disorder (12851637), Bipolar Disorder (22386572), Depressive disorder (12457397), Depressive disorder (23269207), Major Depressive Disorder (20957653), Depressive disorder (18221628), Major Depressive Disorder (24955721), Unipolar Depression (25755794), Unipolar Depression (24093582), Depressive disorder (21215389), Depressive disorder (23619509), Depressive disorder (19517574), Depressive disorder (19548263), Major Depressive Disorder (22480177), Schizophrenia (26899345), Depressive disorder (15999345), Depressive disorder (21937024), Major Depressive Disorder (23537502), Major Depressive Disorder (18317463), Unipolar Depression (17258370), Schizophrenia (20100784), Depressive disorder (22152193), Major Depressive Disorder (22293360), Major Depressive Disorder (11099740), Major Depressive Disorder (22180334), Single major depressive episode (20471034) |
| ENSG00000005339 | *CREBBP* | 3 | Schizophrenia (22198373), Bipolar Disorder (22386572), Attention deficit hyperactivity disorder (18937310) |
| ENSG00000095794 | *CREM* | 2 | Unipolar Depression (22386572), Schizophrenia (22198373) |
| ENSG00000145708 | *CRHBP* | 8 | Major Depressive Disorder (14573312), Major Depressive Disorder (17599466), Depressive disorder (20368512), Depressive disorder (17467808), Depressive disorder (9118350), Schizophrenia (18838498), Bipolar Disorder (17728670), Schizophrenia (21810631) |
| ENSG00000120088 | *CRHR1* | 45 | Depressive disorder (17376150), Unipolar Depression (16815632), Depressive disorder (25422957), Depressive disorder (24933348), Depressive disorder (19220485), Depressive disorder (20383137), Unipolar Depression (22467522), Major Depressive Disorder (23157339), Unipolar Depression (22249355), Major Depressive Disorder (22378896), Depressive disorder (26518448), Depressive disorder (19548263), Major Depressive Disorder (23529111), Depressive disorder (19596121), Depressive disorder (15310462), Depressive disorder (24741566), Depressive disorder (24166410), Depressive disorder (24931706), Depressive disorder (19736354), Depressive disorder (20957648), Unipolar Depression (19844206), Depressive disorder (22748421), Recurrent major depressive episodes (22194899), Depressive disorder (15192151), Depressive disorder (16122764), Bipolar Disorder (24012103), Bipolar Disorder (23068076), Depressive disorder (17585962), Depressive disorder (24867333), Single major depressive episode (21998007), Depressive disorder (21438878), Bipolar Disorder (23962971), Depressive disorder (18250257), Depressive disorder (22957021), Bipolar Disorder (22341483), Depressive disorder (26206446), Major Depressive Disorder (26271720), Depressive disorder (15365580), Major Depressive Disorder (17258395), Depressive disorder (23726670), Depressive disorder (25422958), Depressive disorder (20860876), Schizophrenia (18838498), Depressive disorder (22573456), Depressive disorder (25802844) |
| ENSG00000008405 | *CRY1* | 7 | Bipolar Disorder (15722957), Unipolar Depression (24581835), Depressive disorder (22538398), Depressive disorder (23138696), Schizophrenia (17376600), Bipolar Disorder (20072116), Bipolar Disorder (18228528) |
| ENSG00000121671 | *CRY2* | 10 | Depressive disorder (22538398), Schizophrenia (25799324), Bipolar Disorder (19839995), Depressive disorder (23138696), Depressive disorder (20195522), Bipolar Disorder (20072116), Bipolar Disorder (20856823), Depressive disorder (24099037), Bipolar Disorder (16528748), Bipolar Disorder (18228528) |
| ENSG00000103316 | *CRYM* | 2 | Schizophrenia (19165527), Schizophrenia (19110265) |
| ENSG00000164400 | *CSF2* | 11 | Depressive disorder (10534256), Schizophrenia (15613777), Schizophrenia (17522711), Schizophrenia (18547720), Schizophrenia (19281803), Attention deficit hyperactivity disorder (18577287), Bipolar Disorder (10696830), Depressive disorder (12692775), Schizophrenia (6158928), Schizophrenia (11681838), Autism Spectrum Disorders (9766735) |
| ENSG00000121904 | *CSMD2* | 2 | Depressive disorder (22064162), Schizophrenia (21439553) |
| ENSG00000141551 | *CSNK1D* | 3 | Bipolar Disorder (19839995), Major Depressive Disorder (22981886), Bipolar Disorder (18228528) |
| ENSG00000213923 | *CSNK1E* | 8 | Bipolar Disorder (22743604), Bipolar Disorder (22981886), Bipolar Disorder (12565145), Bipolar Disorder (18228528), Bipolar Disorder (20072116), Bipolar Disorder (26283580), Schizophrenia (22367616), Schizophrenia (19839995) |
| ENSG00000114646 | *CSPG5* | 2 | Schizophrenia (19367581), Schizophrenia (23791194) |
| ENSG00000163599 | *CTLA4* | 7 | Major Depressive Disorder (11555351), Bipolar Disorder (21040781), Schizophrenia (18848621), Schizophrenia (12007590), Bipolar Disorder (14678452), Schizophrenia (25998553), Schizophrenia (23666060) |
| ENSG00000168036 | *CTNNB1* | 3 | Schizophrenia (26027441), Schizophrenia (23598903), Schizophrenia (20615089) |
| ENSG00000169862 | *CTNND2* | 4 | Schizophrenia (24256404), Schizophrenia (19165527), Schizophrenia (18940311), Autism Spectrum Disorders (25106414) |
| ENSG00000205279 | *CTXN3* | 2 | Schizophrenia (25889058), Schizophrenia (22643131) |
| ENSG00000257923 | *CUX1* | 6 | Unipolar Depression (22584459), Major Depressive Disorder (18081157), Major Depressive Disorder (21938001), Unipolar Depression (19376528), Autism Spectrum Disorders (22180456), Major Depressive Disorder (26278479) |
| ENSG00000111249 | *CUX2* | 4 | Bipolar Disorder (17239033), Bipolar Disorder (11353453), Bipolar Disorder (19328558), Bipolar Disorder (15389760) |
| ENSG00000140505 | *CYP1A2* | 30 | Schizophrenia (11817502), Schizophrenia (15564895), Schizophrenia (10850389), Schizophrenia (14695651), Schizophrenia (19806921), Schizophrenia (20143052), Schizophrenia (16969362), Schizophrenia (15505641), Schizophrenia (21142916), Schizophrenia (15206669), Schizophrenia (17688403), Major Depressive Disorder (19095219), Schizophrenia (11179771), Schizophrenia (10889552), Schizophrenia (12618594), Schizophrenia (23601795), Schizophrenia (20691427), Schizophrenia (12790158), Schizophrenia (19000940), Schizophrenia (16775389), Schizophrenia (12943470), Schizophrenia (17611010), Schizophrenia (12630986), Schizophrenia (11186132), Schizophrenia (27021090), Depressive disorder (20595028), Schizophrenia (22901441), Schizophrenia (16490169), Schizophrenia (11496364), Major Depressive Disorder (21121774) |
| ENSG00000110887 | *DAO* | 44 | Schizophrenia (17890006), Bipolar Disorder (19586533), Schizophrenia (15464270), Schizophrenia (17250995), Bipolar Disorder (16380905), Schizophrenia (14966479), Schizophrenia (20483168), Schizophrenia (26440917), Schizophrenia (12625025), Schizophrenia (19439994), Schizophrenia (17408693), Schizophrenia (19591808), Schizophrenia (19729970), Schizophrenia (17492767), Schizophrenia (16203746), Schizophrenia (15953485), Bipolar Disorder (22239582), Schizophrenia (22837388), Schizophrenia (18615285), Schizophrenia (19367581), Schizophrenia (21421061), Schizophrenia (16314870), Schizophrenia (17336946), Schizophrenia (21471957), Schizophrenia (17982252), Schizophrenia (19077230), Schizophrenia (17179078), Schizophrenia (17627036), Schizophrenia (18583979), Schizophrenia (17055463), Schizophrenia (18715757), Bipolar Disorder (18165970), Schizophrenia (23219954), Schizophrenia (19786963), Schizophrenia (22892863), Schizophrenia (12364586), Schizophrenia (21981077), Schizophrenia (21700703), Schizophrenia (16828464), Schizophrenia (19223009), Schizophrenia (26986737), Schizophrenia (23555897), Schizophrenia (16842973), Schizophrenia (15744031) |
| ENSG00000113758 | *DBN1* | 4 | Schizophrenia (16402129), Schizophrenia (23566496), Schizophrenia (15140563), Bipolar Disorder (22760556) |
| ENSG00000146038 | *DCDC2* | 4 | Schizophrenia (21507613), Schizophrenia (20691427), Attention deficit hyperactivity disorder (19362708), Schizophrenia (19944766) |
| ENSG00000132437 | *DDC* | 16 | Major Depressive Disorder (20092830), Schizophrenia (15318031), Attention deficit hyperactivity disorder (17938636), Attention deficit hyperactivity disorder (11443526), Bipolar Disorder (10889538), Attention deficit hyperactivity disorder (12555230), Schizophrenia (11673800), Schizophrenia (20691427), Attention deficit hyperactivity disorder (18163388), Schizophrenia (9387886), Schizophrenia (17948905), Bipolar Disorder (8750358), Bipolar Disorder (10578236), Schizophrenia (25073638), Attention deficit hyperactivity disorder (18821565), Schizophrenia (19944766) |
| ENSG00000175197 | *DDIT3* | 2 | Autism Spectrum Disorders (21364653), Major Depressive Disorder (24703171) |
| ENSG00000204580 | *DDR1* | 2 | Schizophrenia (18836851), Schizophrenia (17440435) |
| ENSG00000100056 | *DGCR14* | 2 | Schizophrenia (16432632), Schizophrenia (12476324) |
| ENSG00000128191 | *DGCR8* | 4 | Schizophrenia (23015298), Schizophrenia (23719809), Depressive disorder (22694265), Schizophrenia (24904170) |
| ENSG00000102780 | *DGKH* | 12 | Bipolar Disorder (22892719), Bipolar Disorder (17486107), Bipolar Disorder (21654738), Bipolar Disorder (21507135), Schizophrenia (24958494), Bipolar Disorder (21771265), Bipolar Disorder (22048461), Bipolar Disorder (19818381), Bipolar Disorder (19478689), Bipolar Disorder (19308021), Bipolar Disorder (19328558), Bipolar Disorder (19358880) |
| ENSG00000117682 | *DHDDS* | 8 | Schizophrenia (17631280), Schizophrenia (18083349), Schizophrenia (26494551), Schizophrenia (17630256), Schizophrenia (11871372), Attention deficit hyperactivity disorder (22564179), Schizophrenia (11087021), Schizophrenia (11295379) |
| ENSG00000100697 | *DICER1* | 3 | Schizophrenia (23904455), Schizophrenia (23015298), Depressive disorder (26632874) |
| ENSG00000150764 | *DIXDC1* | 2 | Depressive disorder (27752079), Schizophrenia (22832659) |
| ENSG00000075711 | *DLG1* | 7 | Schizophrenia (20691406), Schizophrenia (26440542), Schizophrenia (15696539), Schizophrenia (18665322), Schizophrenia (22225629), Schizophrenia (12421351), Schizophrenia (21850710) |
| ENSG00000082458 | *DLG3* | 6 | Schizophrenia (12777268), Schizophrenia (17113057), Major Depressive Disorder (16023328), Schizophrenia (16762023), Major Depressive Disorder (15054476), Bipolar Disorder (18033238) |
| ENSG00000132535 | *DLG4* | 24 | Unipolar Depression (18033238), Bipolar Disorder (17113057), Bipolar Disorder (15054476), Autism Spectrum Disorders (20952458), Schizophrenia (17093888), Schizophrenia (21490598), Schizophrenia (21151988), Depressive disorder (19917299), Unipolar Depression (16023328), Autism Spectrum Disorders (27072977), Schizophrenia (18462708), Schizophrenia (26013316), Schizophrenia (23921260), Schizophrenia (12784099), Schizophrenia (16702973), Schizophrenia (12950712), Schizophrenia (19455133), Unipolar Depression (21635931), Schizophrenia (11043537), Schizophrenia (16762023), Schizophrenia (20385374), Schizophrenia (26555035), Schizophrenia (23936182), Schizophrenia (20921115) |
| ENSG00000170579 | *DLGAP1* | 3 | Schizophrenia (12950712), Schizophrenia (12784099), Schizophrenia (22940546) |
| ENSG00000185559 | *DLK1* | 2 | Schizophrenia (21727898), Schizophrenia (19346103) |
| ENSG00000144355 | *DLX1* | 3 | Schizophrenia (18384059), Autism Spectrum Disorders (21302352), Bipolar Disorder (12963668) |
| ENSG00000163687 | *DNASE1L3* | 4 | Schizophrenia (6870484), Schizophrenia (9387886), Schizophrenia (23176747), Autism Spectrum Disorders (19647026) |
| ENSG00000106976 | *DNM1* | 3 | Schizophrenia (26039597), Schizophrenia (23825400), Schizophrenia (19110265) |
| ENSG00000130816 | *DNMT1* | 13 | Schizophrenia (17270400), Schizophrenia (23938174), Bipolar Disorder (15684088), Bipolar Disorder (25476119), Schizophrenia (14684836), Schizophrenia (17965595), Schizophrenia (15671176), Schizophrenia (15581395), Schizophrenia (24859147), Schizophrenia (24702539), Major Depressive Disorder (21592522), Schizophrenia (19386473), Schizophrenia (17259861) |
| ENSG00000088305 | *DNMT3B* | 5 | Schizophrenia (24859147), Major Depressive Disorder (21592522), Autism Spectrum Disorders (25290267), Schizophrenia (19576953), Schizophrenia (21154337) |
| ENSG00000107447 | *DNTT* | 18 | Schizophrenia (16321170), Schizophrenia (12476324), Schizophrenia (15219469), Attention deficit hyperactivity disorder (18937300), Attention deficit hyperactivity disorder (12497620), Attention deficit hyperactivity disorder (16893529), Attention deficit hyperactivity disorder (12627475), Schizophrenia (18823757), Schizophrenia (16054338), Schizophrenia (18513383), Schizophrenia (20950212), Bipolar Disorder (16958037), Schizophrenia (14755442), Attention deficit hyperactivity disorder (9774775), Attention deficit hyperactivity disorder (11032386), Schizophrenia (11381258), Schizophrenia (9491812), Schizophrenia (19281803) |
| ENSG00000149927 | *DOC2A* | 2 | Schizophrenia (19736351), Schizophrenia (20489179) |
| ENSG00000088538 | *DOCK3* | 3 | Attention deficit hyperactivity disorder (14569117), Attention deficit hyperactivity disorder (19352218), Attention deficit hyperactivity disorder (20032819) |
| ENSG00000175497 | *DPP10* | 5 | Schizophrenia (19736351), Autism Spectrum Disorders (18252227), Bipolar Disorder (23958183), Bipolar Disorder (19308021), Bipolar Disorder (20414141) |
| ENSG00000184845 | *DRD1* | 57 | Unipolar Depression (24322206), Attention deficit hyperactivity disorder (19695183), Schizophrenia (26213377), Bipolar Disorder (20874815), Schizophrenia (10206227), Attention deficit hyperactivity disorder (22404661), Schizophrenia (23042374), Bipolar Disorder (19153942), Schizophrenia (26484506), Schizophrenia (20081237), Schizophrenia (16397404), Depressive disorder (17558292), Attention deficit hyperactivity disorder (18023044), Schizophrenia (20456319), Schizophrenia (17092969), Attention deficit hyperactivity disorder (18937294), Attention deficit hyperactivity disorder (14569274), Schizophrenia (18855532), Schizophrenia (18583979), Schizophrenia (22940547), Major Depressive Disorder (24927283), Depressive disorder (23668904), Schizophrenia (15785860), Attention deficit hyperactivity disorder (17679637), Attention deficit hyperactivity disorder (15717291), Schizophrenia (20127886), Schizophrenia (21181138), Schizophrenia (25179995), Schizophrenia (18451638), Bipolar Disorder (16984965), Schizophrenia (26957229), Schizophrenia (23036699), Schizophrenia (12497608), Bipolar Disorder (25233244), Autism Spectrum Disorders (18205172), Attention deficit hyperactivity disorder (24410775), Bipolar Disorder (12488059), Schizophrenia (17455212), Schizophrenia (7485254), Bipolar Disorder (20071033), Attention deficit hyperactivity disorder (12377397), Attention deficit hyperactivity disorder (17310237), Depressive disorder (17851196), Depressive disorder (20685009), Schizophrenia (8837716), Schizophrenia (15952869), Bipolar Disorder (17066478), Bipolar Disorder (19647327), Schizophrenia (19000940), Schizophrenia (19367581), Schizophrenia (20382433), Major Depressive Disorder (15927089), Bipolar Disorder (15704231), Schizophrenia (18382271), Schizophrenia (21178390), Bipolar Disorder (1385598), Schizophrenia (21955727) |
| ENSG00000149295 | *DRD2* | 247 | Schizophrenia (18175338), Attention deficit hyperactivity disorder (25704081), Attention deficit hyperactivity disorder (25641135), Attention deficit hyperactivity disorder (24780147), Schizophrenia (8886166), Schizophrenia (12210271), Bipolar Disorder (7551964), Depressive disorder (20526230), Schizophrenia (9259374), Bipolar Disorder (1385598), Schizophrenia (22569179), Attention deficit hyperactivity disorder (11840503), Schizophrenia (20179754), Bipolar Disorder (7760324), Bipolar Disorder (18922583), Schizophrenia (8908411), Schizophrenia (15694263), Major Depressive Disorder (25642918), Major Depressive Disorder (15927089), Schizophrenia (16959057), Schizophrenia (16402354), Schizophrenia (12399954), Schizophrenia (7907680), Depressive disorder (11692072), Schizophrenia (12497624), Major Depressive Disorder (22796099), Depressive disorder (23683269), Schizophrenia (18855532), Attention deficit hyperactivity disorder (10578241), Schizophrenia (20664489), Attention deficit hyperactivity disorder (17671965), Schizophrenia (1475034), Schizophrenia (16769201), Schizophrenia (18255274), Schizophrenia (9097961), Attention deficit hyperactivity disorder (11462745), Schizophrenia (18926547), Schizophrenia (12497614), Schizophrenia (9472122), Schizophrenia (17207969), Bipolar Disorder (23044341), Attention deficit hyperactivity disorder (25271118), Schizophrenia (10541004), Schizophrenia (14572625), Schizophrenia (12497608), Schizophrenia (11929577), Attention deficit hyperactivity disorder (22610946), Unipolar Depression (24322206), Autism Spectrum Disorders (20446882), Schizophrenia (24495967), Attention deficit hyperactivity disorder (20150882), Bipolar Disorder (20071033), Attention deficit hyperactivity disorder (23364393), Bipolar Disorder (1677600), Schizophrenia (15850500), Schizophrenia (14509080), Schizophrenia (20716857), Schizophrenia (18496209), Schizophrenia (18426314), Attention deficit hyperactivity disorder (19364291), Schizophrenia (19367581), Attention deficit hyperactivity disorder (10738544), Schizophrenia (9187010), Schizophrenia (17105675), Schizophrenia (15696493), Schizophrenia (26598739), Schizophrenia (8439241), Attention deficit hyperactivity disorder (17044099), Bipolar Disorder (8710185), Schizophrenia (16183199), Schizophrenia (20194480), Attention deficit hyperactivity disorder (18404133), Schizophrenia (12192613), Schizophrenia (26666695), Schizophrenia (10898926), Schizophrenia (8825888), Schizophrenia (20665240), Schizophrenia (14610521), Schizophrenia (11131175), Major Depressive Disorder (18929622), Schizophrenia (17113268), Bipolar Disorder (20874815), Schizophrenia (9514583), Schizophrenia (16973280), Schizophrenia (19770837), Schizophrenia (17092971), Schizophrenia (10719223), Schizophrenia (25829376), Schizophrenia (8837714), Schizophrenia (15048055), Schizophrenia (18451638), Schizophrenia (12617772), Schizophrenia (15211624), Attention deficit hyperactivity disorder (11409697), Schizophrenia (18332877), Schizophrenia (15952869), Attention deficit hyperactivity disorder (18821565), Attention deficit hyperactivity disorder (22531292), Schizophrenia (15140279), Schizophrenia (19913597), Schizophrenia (23066770), Schizophrenia (11245917), Schizophrenia (16513877), Bipolar Disorder (10402492), Schizophrenia (24675081), Bipolar Disorder (8950413), Attention deficit hyperactivity disorder (18580852), Bipolar Disorder (22326841), Major Depressive Disorder (9513185), Schizophrenia (14741327), Attention deficit hyperactivity disorder (16648784), Schizophrenia (10220012), Schizophrenia (18831910), Schizophrenia (17681085), Bipolar Disorder (9754623), Schizophrenia (8471125), Schizophrenia (10831489), Schizophrenia (11740982), Depressive disorder (21540761), Schizophrenia (19634929), Attention deficit hyperactivity disorder (21606926), Bipolar Disorder (21292452), Schizophrenia (19000940), Schizophrenia (22198450), Schizophrenia (18583979), Attention deficit hyperactivity disorder (18188752), Schizophrenia (25056061), Bipolar Disorder (10482338), Unipolar Depression (24555772), Schizophrenia (18418366), Schizophrenia (20375926), Major Depressive Disorder (23696934), Schizophrenia (17366345), Schizophrenia (15051179), Attention deficit hyperactivity disorder (15008324), Schizophrenia (18715757), Schizophrenia (17669630), Schizophrenia (17417059), Schizophrenia (17362435), Bipolar Disorder (25430946), Schizophrenia (8723039), Schizophrenia (19929252), Schizophrenia (2069495), Depressive disorder (16632165), Schizophrenia (8837713), Schizophrenia (19547807), Attention deficit hyperactivity disorder (21844870), Schizophrenia (9850987), Bipolar Disorder (12366879), Bipolar Disorder (12834818), Schizophrenia (7480434), Attention deficit hyperactivity disorder (17931438), Schizophrenia (24120301), Bipolar Disorder (9588764), Schizophrenia (9636132), Schizophrenia (19944766), Schizophrenia (17087792), Schizophrenia (11803529), Attention deficit hyperactivity disorder (24942140), Depressive disorder (23512949), Schizophrenia (11343865), Schizophrenia (19207030), Schizophrenia (7984046), Schizophrenia (21187413), Schizophrenia (12707934), Schizophrenia (11343878), Schizophrenia (21206399), Attention deficit hyperactivity disorder (22832519), Schizophrenia (16867246), Depressive disorder (17585060), Attention deficit hyperactivity disorder (24163823), Schizophrenia (17157291), Attention deficit hyperactivity disorder (15802092), Schizophrenia (18829695), Schizophrenia (19512960), Schizophrenia (22370928), Attention deficit hyperactivity disorder (18030083), Schizophrenia (21861710), Schizophrenia (17982252), Bipolar Disorder (22514151), Schizophrenia (19302829), Schizophrenia (26346037), Schizophrenia (26561806), Schizophrenia (27829443), Schizophrenia (25240594), Schizophrenia (15785860), Schizophrenia (20138949), Schizophrenia (11505224), Schizophrenia (11765615), Schizophrenia (9858029), Bipolar Disorder (24229495), Schizophrenia (19193342), Schizophrenia (20691427), Schizophrenia (8723049), Major Depressive Disorder (12422061), Schizophrenia (10402509), Schizophrenia (15286066), Schizophrenia (24086483), Autism Spectrum Disorders (23851570), Schizophrenia (18957051), Schizophrenia (11256581), Schizophrenia (7824680), Schizophrenia (15108180), Schizophrenia (9339651), Schizophrenia (17455212), Schizophrenia (19393294), Schizophrenia (18477981), Autism Spectrum Disorders (26872113), Schizophrenia (15809404), Schizophrenia (23816932), Schizophrenia (8277546), Bipolar Disorder (11024217), Schizophrenia (19508883), Attention deficit hyperactivity disorder (19352218), Schizophrenia (12708251), Schizophrenia (9650635), Bipolar Disorder (7919920), Schizophrenia (22525159), Schizophrenia (26320194), Schizophrenia (14593428), Bipolar Disorder (11857579), Attention deficit hyperactivity disorder (24410775), Schizophrenia (12762588), Bipolar Disorder (7903509), Schizophrenia (20579747), Schizophrenia (27283386), Schizophrenia (19766158), Schizophrenia (7712120), Schizophrenia (24704945), Bipolar Disorder (10490710), Depressive disorder (21596067), Schizophrenia (1837284), Schizophrenia (7914079), Depressive disorder (12957328), Schizophrenia (20672519), Attention deficit hyperactivity disorder (19914781), Bipolar Disorder (7914141), Schizophrenia (25504812), Depressive disorder (18501970), Schizophrenia (18579277), Schizophrenia (19158809), Depressive disorder (15203798), Schizophrenia (15567074), Schizophrenia (12942993), Single major depressive episode (11728608), Schizophrenia (9713903), Schizophrenia (11304833) |
| ENSG00000151577 | *DRD3* | 163 | Schizophrenia (10379516), Schizophrenia (19897343), Major Depressive Disorder (21630437), Schizophrenia (11149951), Schizophrenia (20456319), Schizophrenia (9514583), Schizophrenia (10889555), Schizophrenia (8135304), Unipolar Depression (8464957), Schizophrenia (16056149), Bipolar Disorder (11857579), Schizophrenia (9702743), Major Depressive Disorder (16583407), Schizophrenia (9577839), Schizophrenia (12497608), Schizophrenia (17429404), Schizophrenia (12497614), Schizophrenia (17171662), Schizophrenia (11400029), Schizophrenia (10395214), Major Depressive Disorder (21680027), Major Depressive Disorder (10697826), Schizophrenia (7909989), Schizophrenia (9674978), Schizophrenia (11121180), Schizophrenia (18987889), Schizophrenia (8678117), Schizophrenia (9034004), Schizophrenia (12960753), Schizophrenia (12632798), Schizophrenia (8225313), Schizophrenia (18043709), Schizophrenia (17698325), Bipolar Disorder (24021960), Schizophrenia (20667458), Schizophrenia (18551040), Schizophrenia (10670776), Attention deficit hyperactivity disorder (11864723), Schizophrenia (18583979), Schizophrenia (18703116), Schizophrenia (9491816), Major Depressive Disorder (22093107), Schizophrenia (18295456), Schizophrenia (9068771), Schizophrenia (26376054), Schizophrenia (8837704), Schizophrenia (7860086), Schizophrenia (18451638), Bipolar Disorder (7573178), Schizophrenia (18496209), Schizophrenia (10402502), Schizophrenia (23932573), Schizophrenia (19508883), Schizophrenia (8641685), Bipolar Disorder (10909128), Schizophrenia (22172931), Schizophrenia (11343865), Bipolar Disorder (19647327), Bipolar Disorder (20071033), Schizophrenia (19302829), Bipolar Disorder (11378841), Bipolar Disorder (8509522), Bipolar Disorder (15539862), Schizophrenia (19367581), Schizophrenia (11673801), Schizophrenia (8825896), Autism Spectrum Disorders (22397633), Autism Spectrum Disorders (25224105), Schizophrenia (14681904), Bipolar Disorder (8098068), Depressive disorder (20685009), Schizophrenia (10523822), Schizophrenia (11063791), Depressive disorder (19235789), Schizophrenia (10490702), Attention deficit hyperactivity disorder (19041097), Autism Spectrum Disorders (25792691), Schizophrenia (26561806), Attention deficit hyperactivity disorder (17671965), Schizophrenia (9017973), Schizophrenia (11179771), Schizophrenia (25158632), Schizophrenia (10402493), Major Depressive Disorder (19095219), Attention deficit hyperactivity disorder (19352218), Schizophrenia (22569179), Schizophrenia (17125970), Schizophrenia (9577838), Schizophrenia (12082567), Schizophrenia (18045777), Schizophrenia (21110120), Bipolar Disorder (17239488), Schizophrenia (15998189), Autism Spectrum Disorders (23032108), Major Depressive Disorder (24927283), Schizophrenia (8780735), Bipolar Disorder (16969276), Bipolar Disorder (11024217), Schizophrenia (9686422), Schizophrenia (18579277), Schizophrenia (15920292), Schizophrenia (10893495), Schizophrenia (9118322), Autism Spectrum Disorders (19058789), Schizophrenia (7914142), Schizophrenia (19691023), Schizophrenia (21963356), Schizophrenia (1362221), Schizophrenia (11104840), Schizophrenia (7903510), Schizophrenia (17924589), Schizophrenia (16893532), Schizophrenia (12605094), Schizophrenia (21948748), Schizophrenia (9106238), Schizophrenia (19766158), Schizophrenia (12207142), Bipolar Disorder (9149327), Schizophrenia (25181639), Schizophrenia (25264289), Schizophrenia (25262640), Bipolar Disorder (10482338), Bipolar Disorder (22877924), Schizophrenia (20100784), Bipolar Disorder (9613861), Attention deficit hyperactivity disorder (18404133), Schizophrenia (15567076), Schizophrenia (15342129), Bipolar Disorder (9129711), Schizophrenia (8775753), Autism Spectrum Disorders (21691864), Schizophrenia (8950407), Schizophrenia (15083167), Depressive disorder (23357086), Bipolar Disorder (12834818), Schizophrenia (8794508), Schizophrenia (7810586), Schizophrenia (21595009), Depressive disorder (17851196), Schizophrenia (15553379), Schizophrenia (15785860), Bipolar Disorder (8493294), Schizophrenia (8723055), Unipolar Depression (11287789), Bipolar Disorder (15917720), Schizophrenia (15695058), Schizophrenia (23571810), Schizophrenia (11490179), Schizophrenia (11478419), Schizophrenia (15643094), Schizophrenia (9800221), Schizophrenia (18472202), Schizophrenia (15051179), Schizophrenia (10869881), Schizophrenia (20672519), Schizophrenia (18320559), Schizophrenia (21737144), Schizophrenia (15626824), Schizophrenia (8517175), Schizophrenia (22940547), Schizophrenia (16788776), Schizophrenia (8994215), Schizophrenia (19000940) |
| ENSG00000102385 | *DRP2* | 4 | Schizophrenia (17105906), Schizophrenia (15858820), Schizophrenia (12679234), Schizophrenia (15027329) |
| ENSG00000138101 | *DTNB* | 2 | Schizophrenia (15124027), Schizophrenia (15184234) |
| ENSG00000120129 | *DUSP1* | 5 | Depressive disorder (20953200), Unipolar Depression (22237309), Depressive disorder (15496935), Depressive disorder (25410305), Depressive disorder (23668904) |
| ENSG00000120875 | *DUSP4* | 2 | Depressive disorder (15496935), Depressive disorder (11331420) |
| ENSG00000139318 | *DUSP6* | 6 | Bipolar Disorder (22155192), Bipolar Disorder (16491131), Bipolar Disorder (19328558), Bipolar Disorder (17239488), Bipolar Disorder (11032376), Depressive disorder (15496935) |
| ENSG00000157540 | *DYRK1A* | 2 | Autism Spectrum Disorders (23160955), Autism Spectrum Disorders (25707398) |
| ENSG00000078401 | *EDN1* | 2 | Major Depressive Disorder (19475602), Depressive disorder (26322793) |
| ENSG00000151617 | *EDNRA* | 6 | Major Depressive Disorder (20493668), Bipolar Disorder (16894394), Attention deficit hyperactivity disorder (21499268), Schizophrenia (21784156), Attention deficit hyperactivity disorder (22897819), Schizophrenia (20158934) |
| ENSG00000184349 | *EFNA5* | 2 | Schizophrenia (10408265), Schizophrenia (17826036) |
| ENSG00000125266 | *EFNB2* | 3 | Schizophrenia (20483485), Schizophrenia (27028544), Schizophrenia (27650867) |
| ENSG00000146648 | *EGFR* | 3 | Major Depressive Disorder (21807767), Major Depressive Disorder (17291372), Schizophrenia (24991953) |
| ENSG00000179388 | *EGR3* | 12 | Schizophrenia (20537399), Schizophrenia (20687139), Schizophrenia (22276163), Bipolar Disorder (19839995), Schizophrenia (26474411), Depressive disorder (19204725), Schizophrenia (20144677), Schizophrenia (21421043), Bipolar Disorder (20633309), Schizophrenia (17360599), Schizophrenia (23962955), Schizophrenia (22692564) |
| ENSG00000013016 | *EHD3* | 3 | Unipolar Depression (24997812), Unipolar Depression (24607927), Major Depressive Disorder (22337703) |
| ENSG00000107105 | *ELAVL2* | 3 | Schizophrenia (21674006), Schizophrenia (22777684), Schizophrenia (22761806) |
| ENSG00000012660 | *ELOVL5* | 2 | Schizophrenia (19195843), Major Depressive Disorder (20863572) |
| ENSG00000164778 | *EN2* | 7 | Autism Spectrum Disorders (16252243), Autism Spectrum Disorders (15024396), Schizophrenia (15123388), Autism Spectrum Disorders (20050924), Autism Spectrum Disorders (25290267), Autism Spectrum Disorders (19615670), Schizophrenia (24730055) |
| ENSG00000154928 | *EPHB1* | 9 | Unipolar Depression (19105200), Attention deficit hyperactivity disorder (19698724), Attention deficit hyperactivity disorder (17876324), Schizophrenia (25450228), Depressive disorder (18591486), Unipolar Depression (26960194), Attention deficit hyperactivity disorder (26678348), Depressive disorder (18081710), Schizophrenia (21041834) |
| ENSG00000091831 | *ESR1* | 24 | Schizophrenia (23497414), Depressive disorder (22051074), Schizophrenia (22001950), Schizophrenia (20801219), Bipolar Disorder (11121195), Major Depressive Disorder (26169989), Depressive disorder (26620113), Bipolar Disorder (12627470), Schizophrenia (11525424), Major Depressive Disorder (17365134), Bipolar Disorder (15576061), Depressive disorder (21804148), Depressive disorder (22901010), Depressive disorder (22094188), Bipolar Disorder (11807414), Schizophrenia (12566932), Bipolar Disorder (22389694), Schizophrenia (18424448), Depressive disorder (22205149), Schizophrenia (18164902), Schizophrenia (19386276), Schizophrenia (19439407), Schizophrenia (20347265), Depressive disorder (24133901) |
| ENSG00000140009 | *ESR2* | 9 | Major Depressive Disorder (17365134), Depressive disorder (22901010), Unipolar Depression (26169989), Schizophrenia (18164902), Schizophrenia (22001950), Depressive disorder (21804148), Depressive disorder (24128867), Depressive disorder (24031087), Schizophrenia (11807414) |
| ENSG00000164089 | *ETNPPL* | 2 | Schizophrenia (18191109), Bipolar Disorder (22241472) |
| ENSG00000181104 | *F2R* | 3 | Attention deficit hyperactivity disorder (12192616), Unipolar Depression (18387137), Schizophrenia (19352591) |
| ENSG00000101981 | *F9* | 4 | Bipolar Disorder (9132265), Bipolar Disorder (2884369), Bipolar Disorder (1980485), Bipolar Disorder (7760986) |
| ENSG00000117480 | *FAAH* | 7 | Bipolar Disorder (21658778), Major Depressive Disorder (20080186), Schizophrenia (15721218), Depressive disorder (24180398), Depressive disorder (22826533), Autism Spectrum Disorders (23799528), Schizophrenia (19193342) |
| ENSG00000134824 | *FADS2* | 5 | Attention deficit hyperactivity disorder (19352218), Bipolar Disorder (20615514), Attention deficit hyperactivity disorder (16893529), Schizophrenia (19195843), Major Depressive Disorder (20863572) |
| ENSG00000026103 | *FAS* | 3 | Schizophrenia (22545112), Depressive disorder (26186532), Attention deficit hyperactivity disorder (21651830) |
| ENSG00000083857 | *FAT1* | 4 | Bipolar Disorder (19328558), Bipolar Disorder (17938632), Depressive disorder (20174623), Bipolar Disorder (16402135) |
| ENSG00000149557 | *FEZ1* | 13 | Schizophrenia (17374448), Schizophrenia (24726361), Schizophrenia (19367581), Schizophrenia (15522253), Schizophrenia (16936715), Schizophrenia (19251251), Schizophrenia (16510495), Schizophrenia (18647754), Schizophrenia (18583979), Schizophrenia (22099459), Schizophrenia (17664024), Schizophrenia (17258902), Schizophrenia (19632097) |
| ENSG00000113578 | *FGF1* | 2 | Schizophrenia (17893707), Schizophrenia (18298822) |
| ENSG00000138685 | *FGF2* | 3 | Schizophrenia (11264663), Depressive disorder (21586822), Depressive disorder (16861106) |
| ENSG00000077782 | *FGFR1* | 10 | Depressive disorder (14695195), Schizophrenia (17893707), Unipolar Depression (18068248), Depressive disorder (19204725), Unipolar Depression (23261523), Unipolar Depression (22989054), Depressive disorder (22361116), Schizophrenia (16861106), Bipolar Disorder (20615089), Schizophrenia (23231877) |
| ENSG00000004478 | *FKBP4* | 7 | Depressive disorder (22581765), Unipolar Depression (19199039), Major Depressive Disorder (19545546), Unipolar Depression (20726698), Depressive disorder (25386878), Major Depressive Disorder (25436518), Depressive disorder (22548329) |
| ENSG00000096060 | *FKBP5* | 47 | Depressive disorder (25386878), Depressive disorder (24655651), Major Depressive Disorder (19095219), Bipolar Disorder (21935478), Unipolar Depression (23406438), Depressive disorder (19676097), Unipolar Depression (20047716), Unipolar Depression (20726698), Bipolar Disorder (25522387), Major Depressive Disorder (25436518), Major Depressive Disorder (25355489), Depressive disorder (23861224), Depressive disorder (20226536), Bipolar Disorder (18180755), Autism Spectrum Disorders (25912394), Schizophrenia (26424418), Unipolar Depression (25522420), Major Depressive Disorder (24856550), Unipolar Depression (19545546), Major Depressive Disorder (23324805), Depressive disorder (22134958), Unipolar Depression (21907973), Depressive disorder (26100613), Depressive disorder (26076833), Major Depressive Disorder (26645208), Unipolar Depression (19199039), Bipolar Disorder (24345775), Depressive disorder (24889341), Depressive disorder (25666308), Depressive disorder (22548329), Unipolar Depression (22237309), Depressive disorder (23429203), Bipolar Disorder (25517604), Depressive disorder (22459275), Schizophrenia (25592294), Depressive disorder (24411633), Depressive disorder (27264499), Depressive disorder (22581765), Depressive disorder (17467808), Depressive disorder (18191112), Depressive disorder (24756342), Schizophrenia (25751398), Depressive disorder (18702710), Depressive disorder (23219223), Depressive disorder (26521051), Single major depressive episode (21865530), Depressive disorder (21654733) |
| ENSG00000136068 | *FLNB* | 2 | Schizophrenia (15318034), Schizophrenia (19054571) |
| ENSG00000115414 | *FN1* | 2 | Schizophrenia (12497612), Attention deficit hyperactivity disorder (22939004) |
| ENSG00000086205 | *FOLH1* | 3 | Schizophrenia (14560319), Depressive disorder (17684227), Schizophrenia (19403271) |
| ENSG00000170345 | *FOS* | 2 | Major Depressive Disorder (23668904), Major Depressive Disorder (25734512) |
| ENSG00000114861 | *FOXP1* | 3 | Autism Spectrum Disorders (20950788), Autism Spectrum Disorders (21572417), Autism Spectrum Disorders (22736078) |
| ENSG00000128573 | *FOXP2* | 10 | Schizophrenia (24360035), Major Depressive Disorder (22404659), Schizophrenia (20649982), Schizophrenia (15653268), Schizophrenia (18248790), Schizophrenia (16538183), Schizophrenia (21334420), Schizophrenia (19018235), Autism Spectrum Disorders (20579107), Autism Spectrum Disorders (21832174) |
| ENSG00000033170 | *FUT8* | 2 | Schizophrenia (21471224), Schizophrenia (25979332) |
| ENSG00000010810 | *FYN* | 7 | Schizophrenia (15098360), Schizophrenia (19468241), Schizophrenia (19102774), Bipolar Disorder (19330793), Schizophrenia (11121167), Schizophrenia (19501919), Schizophrenia (17417065) |
| ENSG00000136928 | *GABBR2* | 2 | Unipolar Depression (24022508), Bipolar Disorder (19308021) |
| ENSG00000022355 | *GABRA1* | 7 | Schizophrenia (17167345), Schizophrenia (15993854), Bipolar Disorder (14706423), Schizophrenia (16172613), Schizophrenia (21677653), Bipolar Disorder (19078961), Schizophrenia (19944766) |
| ENSG00000151834 | *GABRA2* | 2 | Schizophrenia (19736351), Bipolar Disorder (19078961) |
| ENSG00000011677 | *GABRA3* | 7 | Bipolar Disorder (15048654), Bipolar Disorder (11602034), Bipolar Disorder (2574000), Bipolar Disorder (11840313), Bipolar Disorder (8546157), Depressive disorder (20512339), Bipolar Disorder (19078961) |
| ENSG00000109158 | *GABRA4* | 3 | Schizophrenia (20583128), Schizophrenia (19736351), Bipolar Disorder (19078961) |
| ENSG00000145864 | *GABRB2* | 21 | Schizophrenia (18715757), Schizophrenia (18583979), Schizophrenia (16172613), Schizophrenia (16950232), Schizophrenia (16472798), Schizophrenia (16023997), Schizophrenia (14699426), Schizophrenia (17412563), Schizophrenia (20166940), Schizophrenia (17520021), Schizophrenia (19367581), Bipolar Disorder (19909288), Schizophrenia (26561861), Schizophrenia (20221451), Schizophrenia (20404824), Schizophrenia (15993854), Schizophrenia (23638040), Schizophrenia (16983389), Bipolar Disorder (19078961), Schizophrenia (19763268), Schizophrenia (17167345) |
| ENSG00000166206 | *GABRB3* | 16 | Bipolar Disorder (11378843), Schizophrenia (19736351), Depressive disorder (22082659), Autism Spectrum Disorders (17230033), Schizophrenia (24865167), Depressive disorder (22414661), Autism Spectrum Disorders (15952184), Schizophrenia (16023997), Autism Spectrum Disorders (22037176), Schizophrenia (17471287), Bipolar Disorder (19078961), Bipolar Disorder (20583128), Depressive disorder (11711165), Depressive disorder (19268543), Autism Spectrum Disorders (19430570), Depressive disorder (15296817) |
| ENSG00000163285 | *GABRG1* | 4 | Schizophrenia (19909288), Bipolar Disorder (19078961), Autism Spectrum Disorders (21832240), Schizophrenia (19736351) |
| ENSG00000094755 | *GABRP* | 2 | Bipolar Disorder (19078961), Schizophrenia (16172613) |
| ENSG00000111886 | *GABRR2* | 2 | Bipolar Disorder (20889312), Bipolar Disorder (19078961) |
| ENSG00000136750 | *GAD2* | 11 | Schizophrenia (15114630), Unipolar Depression (26848839), Schizophrenia (24993056), Major Depressive Disorder (15560956), Unipolar Depression (19229853), Schizophrenia (20659789), Schizophrenia (17412563), Schizophrenia (18923069), Schizophrenia (21677653), Schizophrenia (19125103), Major Depressive Disorder (21226980) |
| ENSG00000182687 | *GALR2* | 3 | Depressive disorder (24706871), Unipolar Depression (21672612), Depressive disorder (18554714) |
| ENSG00000172020 | *GAP43* | 7 | Schizophrenia (21145444), Bipolar Disorder (11208668), Schizophrenia (9881859), Depressive disorder (9774774), Schizophrenia (22138049), Schizophrenia (17012697), Schizophrenia (18622782) |
| ENSG00000107485 | *GATA3* | 2 | Depressive disorder (24813065), Depressive disorder (21937687) |
| ENSG00000137880 | *GCHFR* | 2 | Major Depressive Disorder (20351752), Schizophrenia (21772061) |
| ENSG00000001084 | *GCLC* | 3 | Schizophrenia (17921251), Schizophrenia (20659789), Schizophrenia (21105962) |
| ENSG00000023909 | *GCLM* | 5 | Schizophrenia (18991850), Schizophrenia (20061124), Schizophrenia (19455074), Schizophrenia (18449862), Schizophrenia (16909399) |
| ENSG00000131095 | *GFAP* | 12 | Bipolar Disorder (15885920), Bipolar Disorder (19488045), Schizophrenia (22875919), Unipolar Depression (18585900), Depressive disorder (25098258), Major Depressive Disorder (15238995), Schizophrenia (19405953), Depressive disorder (11754076), Schizophrenia (19034380), Major Depressive Disorder (19000745), Schizophrenia (19110265), Autism Spectrum Disorders (25846779) |
| ENSG00000157017 | *GHRL* | 3 | Schizophrenia (19193342), Schizophrenia (22369141), Depressive disorder (19272368) |
| ENSG00000108262 | *GIT1* | 2 | Attention deficit hyperactivity disorder (21499268), Attention deficit hyperactivity disorder (22897819) |
| ENSG00000106571 | *GLI3* | 2 | Autism Spectrum Disorders (23285124), Autism Spectrum Disorders (22009741) |
| ENSG00000115419 | *GLS* | 3 | Schizophrenia (17669570), Schizophrenia (25315318), Schizophrenia (20659789) |
| ENSG00000141404 | *GNAL* | 5 | Schizophrenia (16044173), Schizophrenia (11317223), Bipolar Disorder (11032382), Bipolar Disorder (19328558), Bipolar Disorder (12782961) |
| ENSG00000087258 | *GNAO1* | 8 | Schizophrenia (8929760), Schizophrenia (9050131), Schizophrenia (14741323), Schizophrenia (21512575), Schizophrenia (9516671), Schizophrenia (8942447), Schizophrenia (8435383), Schizophrenia (11423178) |
| ENSG00000111664 | *GNB3* | 27 | Major Depressive Disorder (14647404), Unipolar Depression (25451402), Major Depressive Disorder (24882179), Bipolar Disorder (19328558), Depressive disorder (12075862), Bipolar Disorder (12366879), Major Depressive Disorder (17066254), Schizophrenia (17521439), Unipolar Depression (20826553), Unipolar Depression (17943025), Major Depressive Disorder (22222462), Unipolar Depression (18325652), Depressive disorder (16897596), Schizophrenia (17726725), Bipolar Disorder (11317211), Depressive disorder (15057736), Schizophrenia (19193342), Schizophrenia (16139171), Depressive disorder (21709600), Bipolar Disorder (20814328), Major Depressive Disorder (16302021), Depressive disorder (25037115), Depressive disorder (17621167), Schizophrenia (20691427), Major Depressive Disorder (17938638), Schizophrenia (16449941), Major Depressive Disorder (19560507) |
| ENSG00000163938 | *GNL3* | 2 | Schizophrenia (19367581), Bipolar Disorder (19416921) |
| ENSG00000164604 | *GPR85* | 2 | Schizophrenia (18413613), Schizophrenia (22968816) |
| ENSG00000111291 | *GPRC5D* | 4 | Major Depressive Disorder (18075476), Bipolar Disorder (15452587), Bipolar Disorder (20371266), Bipolar Disorder (16389273) |
| ENSG00000177885 | *GRB2* | 2 | Schizophrenia (21195589), Schizophrenia (18658164) |
| ENSG00000155511 | *GRIA1* | 21 | Bipolar Disorder (18484081), Schizophrenia (9099808), Bipolar Disorder (17942280), Depressive disorder (23990563), Schizophrenia (23053966), Depressive disorder (23262314), Unipolar Depression (22057216), Schizophrenia (16526023), Bipolar Disorder (22122651), Bipolar Disorder (18444252), Schizophrenia (22094384), Depressive disorder (24895223), Depressive disorder (22534499), Schizophrenia (7609609), Depressive disorder (24725413), Depressive disorder (24048851), Schizophrenia (15696539), Schizophrenia (17982252), Schizophrenia (21693126), Bipolar Disorder (17299517), Schizophrenia (25224260) |
| ENSG00000120251 | *GRIA2* | 11 | Major Depressive Disorder (22057216), Schizophrenia (18184693), Unipolar Depression (23613500), Bipolar Disorder (22122651), Bipolar Disorder (19448189), Schizophrenia (18163426), Bipolar Disorder (17299517), Schizophrenia (9099808), Schizophrenia (7609609), Schizophrenia (20347265), Schizophrenia (22094384) |
| ENSG00000125675 | *GRIA3* | 11 | Bipolar Disorder (20226637), Depressive disorder (22534499), Bipolar Disorder (17299517), Schizophrenia (18163426), Depressive disorder (19548263), Schizophrenia (23149219), Bipolar Disorder (10644433), Bipolar Disorder (15526294), Depressive disorder (21966062), Schizophrenia (19736351), Bipolar Disorder (15264227) |
| ENSG00000152578 | *GRIA4* | 10 | Schizophrenia (16983646), Schizophrenia (18163426), Schizophrenia (15450689), Major Depressive Disorder (23613500), Schizophrenia (22094384), Unipolar Depression (22057216), Schizophrenia (12497607), Schizophrenia (19573479), Bipolar Disorder (22122651), Schizophrenia (19125103) |
| ENSG00000182771 | *GRID1* | 8 | Schizophrenia (19264453), Depressive disorder (22412961), Bipolar Disorder (16380905), Schizophrenia (17490860), Schizophrenia (19346103), Schizophrenia (23017809), Depressive disorder (22064162), Depressive disorder (23560106) |
| ENSG00000163873 | *GRIK3* | 10 | Schizophrenia (11986986), Schizophrenia (15897672), Schizophrenia (16325263), Schizophrenia (19995671), Schizophrenia (19022628), Schizophrenia (19125103), Schizophrenia (15886719), Schizophrenia (19921975), Depressive disorder (20052609), Bipolar Disorder (16958029) |
| ENSG00000149403 | *GRIK4* | 11 | Bipolar Disorder (22052594), Bipolar Disorder (23357115), Bipolar Disorder (17449450), Schizophrenia (18289755), Schizophrenia (16819533), Bipolar Disorder (18824690), Unipolar Depression (22222462), Schizophrenia (16325263), Schizophrenia (19125103), Bipolar Disorder (19328558), Autism Spectrum Disorders (26446216) |
| ENSG00000105737 | *GRIK5* | 4 | Schizophrenia (16325263), Schizophrenia (19125103), Schizophrenia (18923069), Bipolar Disorder (19086053) |
| ENSG00000273079 | *GRIN2B* | 49 | Schizophrenia (18033238), Bipolar Disorder (16380905), Schizophrenia (26876050), Attention deficit hyperactivity disorder (17010153), Schizophrenia (20347576), Schizophrenia (16549338), Depressive disorder (23082852), Schizophrenia (12476325), Schizophrenia (12824739), Schizophrenia (11807413), Bipolar Disorder (15952869), Schizophrenia (21827795), Schizophrenia (16762023), Depressive disorder (19838302), Schizophrenia (18583979), Schizophrenia (21919190), Schizophrenia (15841096), Bipolar Disorder (19328558), Depressive disorder (24584520), Bipolar Disorder (18007143), Schizophrenia (16266783), Bipolar Disorder (24490167), Schizophrenia (15211626), Schizophrenia (22781170), Bipolar Disorder (19005876), Autism Spectrum Disorders (22326929), Schizophrenia (11317224), Autism Spectrum Disorders (23918416), Schizophrenia (23660601), Schizophrenia (26421900), Bipolar Disorder (20537720), Schizophrenia (23070074), Bipolar Disorder (20957330), Autism Spectrum Disorders (21572417), Schizophrenia (17669510), Schizophrenia (26257337), Schizophrenia (26020650), Autism Spectrum Disorders (24126926), Major Depressive Disorder (21635931), Autism Spectrum Disorders (22833210), Schizophrenia (17224684), Depressive disorder (20357110), Bipolar Disorder (17299517), Unipolar Depression (24114429), Bipolar Disorder (26563126), Schizophrenia (15054476), Schizophrenia (19367581), Depressive disorder (21814183), Schizophrenia (10910800) |
| ENSG00000161509 | *GRIN2C* | 7 | Schizophrenia (18033238), Bipolar Disorder (26013316), Schizophrenia (23070074), Bipolar Disorder (18444252), Schizophrenia (24556017), Schizophrenia (21919190), Schizophrenia (14684436) |
| ENSG00000198785 | *GRIN3A* | 4 | Bipolar Disorder (15474907), Schizophrenia (23237318), Schizophrenia (19665356), Schizophrenia (26257337) |
| ENSG00000155974 | *GRIP1* | 2 | Schizophrenia (17303296), Schizophrenia (19736351) |
| ENSG00000152822 | *GRM1* | 9 | Depressive disorder (21962378), Schizophrenia (22448230), Depressive disorder (17270300), Attention deficit hyperactivity disorder (22138692), Schizophrenia (25137254), Depressive disorder (23045678), Depressive disorder (11642544), Depressive disorder (24270186), Schizophrenia (15945063) |
| ENSG00000164082 | *GRM2* | 16 | Schizophrenia (17531207), Schizophrenia (11317221), Schizophrenia (18759551), Schizophrenia (7609609), Schizophrenia (19367581), Schizophrenia (26758213), Schizophrenia (18853337), Schizophrenia (20211215), Schizophrenia (9099808), Schizophrenia (19707855), Schizophrenia (18297054), Schizophrenia (21173788), Schizophrenia (18923069), Schizophrenia (17982252), Schizophrenia (23149219), Major Depressive Disorder (19386277) |
| ENSG00000198822 | *GRM3* | 46 | Schizophrenia (19439994), Major Depressive Disorder (19386277), Schizophrenia (24498053), Schizophrenia (16585454), Schizophrenia (18614340), Schizophrenia (15913960), Schizophrenia (17006672), Schizophrenia (17531207), Schizophrenia (15567072), Schizophrenia (25914064), Schizophrenia (17982252), Bipolar Disorder (20226637), Schizophrenia (25583490), Schizophrenia (16904291), Schizophrenia (19403271), Bipolar Disorder (20957330), Schizophrenia (16380905), Schizophrenia (18256595), Schizophrenia (16417579), Schizophrenia (11840505), Schizophrenia (15310849), Schizophrenia (19482054), Schizophrenia (17636131), Schizophrenia (19707855), Schizophrenia (16365481), Schizophrenia (12782962), Schizophrenia (20638435), Schizophrenia (21344500), Schizophrenia (25209194), Bipolar Disorder (23575746), Schizophrenia (15892884), Schizophrenia (22728822), Schizophrenia (18541626), Schizophrenia (27296644), Schizophrenia (26187343), Schizophrenia (17948896), Schizophrenia (18853337), Bipolar Disorder (19328558), Schizophrenia (18583979), Schizophrenia (18197082), Schizophrenia (17726000), Schizophrenia (24680030), Schizophrenia (21281445), Schizophrenia (19367581), Schizophrenia (25096017), Schizophrenia (19125103) |
| ENSG00000030582 | *GRN* | 6 | Schizophrenia (22505994), Bipolar Disorder (24499389), Schizophrenia (20087814), Depressive disorder (20667979), Depressive disorder (22895706), Depressive disorder (20400120) |
| ENSG00000126010 | *GRPR* | 2 | Autism Spectrum Disorders (18393381), Depressive disorder (21254899) |
| ENSG00000105723 | *GSK3A* | 5 | Bipolar Disorder (20357757), Schizophrenia (16223876), Schizophrenia (20113358), Schizophrenia (25433637), Schizophrenia (18500637) |
| ENSG00000082701 | *GSK3B* | 51 | Major Depressive Disorder (24927694), Major Depressive Disorder (20682308), Depressive disorder (21315744), Bipolar Disorder (24992082), Major Depressive Disorder (24677591), Major Depressive Disorder (23030648), Bipolar Disorder (28099358), Bipolar Disorder (16733521), Bipolar Disorder (22990942), Depressive disorder (19815943), Bipolar Disorder (20615089), Recurrent major depressive episodes (20219685), Bipolar Disorder (20357757), Bipolar Disorder (14729229), Depressive disorder (20534517), Bipolar Disorder (16289845), Depressive disorder (17628506), Bipolar Disorder (25345732), Bipolar Disorder (15351432), Major Depressive Disorder (20033742), Schizophrenia (25041379), Bipolar Disorder (17357145), Bipolar Disorder (24929537), Major Depressive Disorder (20618448), Bipolar Disorder (21549170), Schizophrenia (20113358), Schizophrenia (15719395), Major Depressive Disorder (18195729), Bipolar Disorder (17621172), Bipolar Disorder (16861141), Schizophrenia (15179015), Schizophrenia (18855532), Schizophrenia (18500637), Bipolar Disorder (16289783), Bipolar Disorder (15694273), Schizophrenia (16987250), Major Depressive Disorder (26186530), Schizophrenia (15254796), Depressive disorder (23188793), Schizophrenia (23440732), Bipolar Disorder (16787706), Schizophrenia (17368486), Schizophrenia (25380769), Schizophrenia (23160851), Schizophrenia (14745448), Schizophrenia (22832527), Bipolar Disorder (26267417), Attention deficit hyperactivity disorder (22658982), Schizophrenia (23598903), Major Depressive Disorder (24001941), Schizophrenia (14518171) |
| ENSG00000063854 | *HAGH* | 2 | Schizophrenia (25645869), Autism Spectrum Disorders (24671236) |
| ENSG00000180353 | *HCLS1* | 2 | Major Depressive Disorder (26278479), Major Depressive Disorder (18081157) |
| ENSG00000164588 | *HCN1* | 3 | Depressive disorder (23033536), Attention deficit hyperactivity disorder (18081024), Depressive disorder (22884333) |
| ENSG00000161610 | *HCRT* | 8 | Major Depressive Disorder (21211849), Depressive disorder (19654034), Depressive disorder (24485472), Depressive disorder (24874707), Depressive disorder (23510906), Depressive disorder (23398442), Depressive disorder (25190041), Schizophrenia (17999203) |
| ENSG00000121764 | *HCRTR1* | 4 | Schizophrenia (17999203), Schizophrenia (15978554), Depressive disorder (22617356), Depressive disorder (21377495) |
| ENSG00000108840 | *HDAC5* | 4 | Major Depressive Disorder (17258370), Depressive disorder (16501568), Depressive disorder (24495952), Attention deficit hyperactivity disorder (18937310) |
| ENSG00000048052 | *HDAC9* | 4 | Schizophrenia (25445625), Schizophrenia (20471694), Depressive disorder (19759294), Unipolar Depression (19767015) |
| ENSG00000010704 | *HFE* | 4 | Autism Spectrum Disorders (20808228), Autism Spectrum Disorders (19167444), Attention deficit hyperactivity disorder (26710823), Schizophrenia (21643746) |
| ENSG00000100644 | *HIF1A* | 2 | Major Depressive Disorder (23333658), Schizophrenia (16632332) |
| ENSG00000130787 | *HIP1R* | 2 | Bipolar Disorder (15588756), Bipolar Disorder (17239488) |
| ENSG00000256269 | *HMBS* | 6 | Schizophrenia (1358185), Schizophrenia (1358782), Schizophrenia (8439242), Schizophrenia (8094629), Schizophrenia (8475213), Schizophrenia (2069495) |
| ENSG00000152413 | *HOMER1* | 13 | Schizophrenia (26555035), Depressive disorder (20673876), Schizophrenia (16011574), Depressive disorder (22072696), Schizophrenia (22749857), Schizophrenia (12815733), Depressive disorder (22267161), Schizophrenia (16354916), Major Depressive Disorder (27964944), Schizophrenia (24472577), Schizophrenia (19165527), Schizophrenia (17317002), Depressive disorder (26641965) |
| ENSG00000105991 | *HOXA1* | 2 | Autism Spectrum Disorders (22359339), Autism Spectrum Disorders (21980499) |
| ENSG00000196639 | *HRH1* | 4 | Schizophrenia (12218662), Schizophrenia (19193342), Schizophrenia (12429384), Schizophrenia (1912125) |
| ENSG00000113749 | *HRH2* | 4 | Schizophrenia (10822343), Schizophrenia (19193342), Schizophrenia (12429384), Schizophrenia (9154248) |
| ENSG00000101180 | *HRH3* | 2 | Schizophrenia (21652606), Schizophrenia (19193342) |
| ENSG00000166598 | *HSP90B1* | 3 | Bipolar Disorder (17805476), Bipolar Disorder (18771604), Bipolar Disorder (19125190) |
| ENSG00000179546 | *HTR1D* | 6 | Depressive disorder (8018797), Schizophrenia (18855532), Schizophrenia (16059583), Depressive disorder (21937687), Schizophrenia (8104876), Attention deficit hyperactivity disorder (17099886) |
| ENSG00000149305 | *HTR3B* | 12 | Schizophrenia (15389765), Unipolar Depression (16487942), Bipolar Disorder (27706728), Depressive disorder (19741568), Major Depressive Disorder (18982004), Major Depressive Disorder (18184810), Schizophrenia (19794330), Schizophrenia (18807291), Schizophrenia (20356718), Bipolar Disorder (22832903), Schizophrenia (21184810), Bipolar Disorder (19328558) |
| ENSG00000164270 | *HTR4* | 13 | Schizophrenia (19892407), Depressive disorder (19476548), Attention deficit hyperactivity disorder (19352218), Depressive disorder (25522384), Schizophrenia (12399948), Schizophrenia (12898568), Attention deficit hyperactivity disorder (16563621), Depressive disorder (20060867), Schizophrenia (18359159), Schizophrenia (15205874), Depressive disorder (20962330), Schizophrenia (22842674), Bipolar Disorder (19328558) |
| ENSG00000158748 | *HTR6* | 18 | Schizophrenia (11524147), Schizophrenia (12165372), Major Depressive Disorder (19095219), Schizophrenia (10206228), Schizophrenia (15205874), Schizophrenia (11163544), Schizophrenia (10893499), Schizophrenia (22745941), Schizophrenia (12579508), Schizophrenia (18855532), Schizophrenia (14741325), Schizophrenia (19193342), Schizophrenia (16005519), Depressive disorder (26449188), Schizophrenia (15048641), Schizophrenia (18583979), Schizophrenia (12057822), Schizophrenia (10477121) |
| ENSG00000197386 | *HTT* | 6 | Depressive disorder (25583186), Schizophrenia (11121205), Depressive disorder (23678106), Depressive disorder (15548484), Unipolar Depression (20360314), Depressive disorder (23697793) |
| ENSG00000131203 | *IDO1* | 4 | Depressive disorder (22751107), Depressive disorder (23866724), Major Depressive Disorder (22683764), Depressive disorder (21693183) |
| ENSG00000111537 | *IFNG* | 14 | Depressive disorder (12084660), Schizophrenia (22623148), Unipolar Depression (23597432), Schizophrenia (21255481), Major Depressive Disorder (23608119), Autism Spectrum Disorders (18762240), Schizophrenia (23083632), Major Depressive Disorder (18077563), Bipolar Disorder (21728784), Depressive disorder (21161299), Depressive disorder (27049572), Autism Spectrum Disorders (25912394), Schizophrenia (20132993), Schizophrenia (9075278) |
| ENSG00000017427 | *IGF1* | 13 | Depressive disorder (24878678), Depressive disorder (22189158), Schizophrenia (17044098), Bipolar Disorder (21302346), Schizophrenia (18973876), Bipolar Disorder (25740013), Bipolar Disorder (23619527), Depressive disorder (8938649), Depressive disorder (21524689), Schizophrenia (19394958), Depressive disorder (19056428), Depressive disorder (25151093), Depressive disorder (25583186) |
| ENSG00000115457 | *IGFBP2* | 5 | Bipolar Disorder (17395163), Major Depressive Disorder (9006397), Major Depressive Disorder (24126930), Bipolar Disorder (15762851), Bipolar Disorder (17230047) |
| ENSG00000263528 | *IKBKE* | 2 | Major Depressive Disorder (19786281), Unipolar Depression (25798331) |
| ENSG00000136634 | *IL10* | 23 | Schizophrenia (11952921), Schizophrenia (14563376), Schizophrenia (11922883), Unipolar Depression (19786281), Schizophrenia (19153889), Schizophrenia (21216474), Schizophrenia (16378687), Schizophrenia (24603720), Depressive disorder (10229162), Schizophrenia (15374585), Schizophrenia (23951054), Unipolar Depression (23608119), Schizophrenia (18583979), Schizophrenia (27969076), Schizophrenia (21658228), Schizophrenia (26481614), Major Depressive Disorder (19087313), Schizophrenia (17066477), Schizophrenia (20393813), Schizophrenia (16905295), Depressive disorder (24143878), Depressive disorder (22921153), Schizophrenia (12667161) |
| ENSG00000150782 | *IL18* | 8 | Autism Spectrum Disorders (11263769), Depressive disorder (11226333), Schizophrenia (21510800), Depressive disorder (16289586), Schizophrenia (18092318), Schizophrenia (26736035), Depressive disorder (21962565), Autism Spectrum Disorders (26728085) |
| ENSG00000115008 | *IL1A* | 25 | Depressive disorder (22990943), Depressive disorder (26002078), Depressive disorder (21722710), Depressive disorder (10483047), Schizophrenia (17610660), Schizophrenia (25858413), Schizophrenia (20347268), Major Depressive Disorder (7506108), Schizophrenia (16856121), Bipolar Disorder (19125864), Depressive disorder (20574126), Autism Spectrum Disorders (26728085), Depressive disorder (19158434), Schizophrenia (15318032), Schizophrenia (17510951), Bipolar Disorder (19488045), Bipolar Disorder (25855618), Schizophrenia (14563376), Schizophrenia (14997019), Schizophrenia (11423178), Schizophrenia (10208450), Schizophrenia (12804791), Schizophrenia (18583979), Depressive disorder (24103372), Depressive disorder (26934083) |
| ENSG00000115594 | *IL1R1* | 2 | Schizophrenia (20347268), Schizophrenia (14563376) |
| ENSG00000136689 | *IL1RN* | 10 | Bipolar Disorder (15318032), Schizophrenia (20347268), Bipolar Disorder (23406623), Schizophrenia (25284335), Schizophrenia (16019233), Schizophrenia (16905295), Bipolar Disorder (19125864), Schizophrenia (18583979), Attention deficit hyperactivity disorder (11803448), Schizophrenia (14563376) |
| ENSG00000109471 | *IL2* | 11 | Schizophrenia (18574615), Major Depressive Disorder (2135065), Schizophrenia (16091861), Schizophrenia (24564241), Schizophrenia (20926141), Attention Deficit Disorder (10889591), Major Depressive Disorder (12635532), Schizophrenia (23487197), Schizophrenia (9075278), Schizophrenia (24065520), Schizophrenia (8546160) |
| ENSG00000100385 | *IL2RB* | 4 | Bipolar Disorder (16380905), Major Depressive Disorder (26278479), Unipolar Depression (18081157), Schizophrenia (8546160) |
| ENSG00000133731 | *IMPA1* | 3 | Bipolar Disorder (20153384), Bipolar Disorder (14699425), Bipolar Disorder (9322233) |
| ENSG00000141401 | *IMPA2* | 12 | Bipolar Disorder (17340635), Bipolar Disorder (20800640), Bipolar Disorder (14699425), Bipolar Disorder (20153384), Bipolar Disorder (17251911), Schizophrenia (11317223), Bipolar Disorder (10822344), Bipolar Disorder (10822345), Depressive disorder (16841073), Schizophrenia (14690303), Bipolar Disorder (15505643), Bipolar Disorder (11673796) |
| ENSG00000151689 | *INPP1* | 4 | Bipolar Disorder (11901356), Bipolar Disorder (16787706), Bipolar Disorder (9682271), Bipolar Disorder (10780272) |
| ENSG00000027644 | *INSRR* | 2 | Schizophrenia (12860771), Depressive disorder (25670333) |
| ENSG00000164941 | *INTS8* | 2 | Attention deficit hyperactivity disorder (24487615), Attention deficit hyperactivity disorder (20049490) |
| ENSG00000065150 | *IPO5* | 4 | Schizophrenia (16644122), Schizophrenia (17458142), Schizophrenia (20542336), Schizophrenia (15364420) |
| ENSG00000016082 | *ISL1* | 2 | Attention deficit hyperactivity disorder (18081024), Bipolar Disorder (18496208) |
| ENSG00000005961 | *ITGA2B* | 2 | Major Depressive Disorder (15473915), Schizophrenia (12399140) |
| ENSG00000055955 | *ITIH4* | 4 | Schizophrenia (19367581), Schizophrenia (21926972), Bipolar Disorder (25136889), Bipolar Disorder (21926974) |
| ENSG00000150995 | *ITPR1* | 3 | Bipolar Disorder (8115665), Bipolar Disorder (16691292), Autism Spectrum Disorders (26393489) |
| ENSG00000008083 | *JARID2* | 3 | Schizophrenia (19884986), Schizophrenia (16967465), Autism Spectrum Disorders (21308764) |
| ENSG00000172977 | *KAT5* | 3 | Bipolar Disorder (20038946), Schizophrenia (15999343), Schizophrenia (9857971) |
| ENSG00000055118 | *KCNH2* | 4 | Schizophrenia (21936766), Schizophrenia (19412172), Schizophrenia (20507645), Schizophrenia (22706279) |
| ENSG00000157542 | *KCNJ6* | 3 | Depressive disorder (22308328), Bipolar Disorder (19567891), Depressive disorder (20943350) |
| ENSG00000143603 | *KCNN3* | 23 | Bipolar Disorder (11526470), Schizophrenia (9866821), Schizophrenia (16393881), Bipolar Disorder (12960745), Schizophrenia (11395478), Schizophrenia (9858366), Schizophrenia (20933057), Schizophrenia (12808432), Schizophrenia (12497613), Schizophrenia (12007452), Schizophrenia (10395215), Bipolar Disorder (11311923), Bipolar Disorder (11121173), Bipolar Disorder (11532529), Schizophrenia (10395218), Bipolar Disorder (15389773), Schizophrenia (10402501), Schizophrenia (21433290), Bipolar Disorder (12898569), Schizophrenia (9672903), Schizophrenia (16086287), Schizophrenia (10196711), Bipolar Disorder (10395209) |
| ENSG00000184156 | *KCNQ3* | 3 | Schizophrenia (18545987), Bipolar Disorder (21176025), Bipolar Disorder (25041603) |
| ENSG00000170745 | *KCNS3* | 2 | Schizophrenia (22937123), Schizophrenia (24170294) |
| ENSG00000128052 | *KDR* | 3 | Major Depressive Disorder (18180758), Schizophrenia (21647420), Depressive disorder (23164459) |
| ENSG00000088247 | *KHSRP* | 2 | Unipolar Depression (18081157), Major Depressive Disorder (26278479) |
| ENSG00000134313 | *KIDINS220* | 2 | Depressive disorder (26118823), Schizophrenia (20570494) |
| ENSG00000117245 | *KIF17* | 3 | Schizophrenia (26421900), Schizophrenia (20646681), Schizophrenia (25354644) |
| ENSG00000067082 | *KLF6* | 2 | Schizophrenia (17387318), Bipolar Disorder (19958095) |
| ENSG00000117009 | *KMO* | 8 | Unipolar Depression (22683764), Schizophrenia (16716206), Schizophrenia (25464917), Schizophrenia (21693093), Schizophrenia (23459468), Unipolar Depression (21492941), Schizophrenia (26142836), Schizophrenia (21727251) |
| ENSG00000118058 | *KMT2A* | 2 | Schizophrenia (18814864), Schizophrenia (17942719) |
| ENSG00000001631 | *KRIT1* | 6 | Schizophrenia (10696830), Schizophrenia (20157312), Schizophrenia (9098580), Depressive disorder (15820228), Schizophrenia (23153507), Schizophrenia (11681838) |
| ENSG00000137944 | *KYAT3* | 3 | Major Depressive Disorder (21492941), Attention deficit hyperactivity disorder (25346392), Schizophrenia (19165527) |
| ENSG00000138136 | *LBX1* | 3 | Bipolar Disorder (12963668), Schizophrenia (17541950), Autism Spectrum Disorders (21302352) |
| ENSG00000174697 | *LEP* | 19 | Major Depressive Disorder (9754619), Schizophrenia (15864111), Bipolar Disorder (20814328), Depressive disorder (23026132), Depressive disorder (25629253), Schizophrenia (25284335), Major Depressive Disorder (16936761), Schizophrenia (25304226), Schizophrenia (20591628), Depressive disorder (17275915), Depressive disorder (19382181), Schizophrenia (14720418), Schizophrenia (17502770), Schizophrenia (17285096), Depressive disorder (22408745), Depressive disorder (23251447), Schizophrenia (18681781), Depressive disorder (22932068), Schizophrenia (18515891) |
| ENSG00000116678 | *LEPR* | 3 | Schizophrenia (20591628), Schizophrenia (17285096), Schizophrenia (21050724) |
| ENSG00000131981 | *LGALS3* | 2 | Attention deficit hyperactivity disorder (25724585), Attention deficit hyperactivity disorder (20557304) |
| ENSG00000107902 | *LHPP* | 2 | Unipolar Depression (26176920), Major Depressive Disorder (18268499) |
| ENSG00000106852 | *LHX6* | 3 | Schizophrenia (22983435), Schizophrenia (25464914), Schizophrenia (22937123) |
| ENSG00000128342 | *LIF* | 2 | Schizophrenia (19879916), Depressive disorder (14970834) |
| ENSG00000114988 | *LMAN2L* | 2 | Attention deficit hyperactivity disorder (23712748), Bipolar Disorder (24914473) |
| ENSG00000048540 | *LMO3* | 53 | Attention deficit hyperactivity disorder (17440935), Attention deficit hyperactivity disorder (20957668), Attention deficit hyperactivity disorder (16585476), Attention deficit hyperactivity disorder (20736997), Attention deficit hyperactivity disorder (16526026), Attention deficit hyperactivity disorder (16342279), Attention deficit hyperactivity disorder (19196467), Attention deficit hyperactivity disorder (15578613), Attention deficit hyperactivity disorder (12377397), Attention deficit hyperactivity disorder (17157268), Attention deficit hyperactivity disorder (17044101), Depressive disorder (18181793), Attention deficit hyperactivity disorder (22749356), Attention deficit hyperactivity disorder (22182296), Depressive disorder (26991370), Attention deficit hyperactivity disorder (20053379), Attention deficit hyperactivity disorder (24132904), Attention deficit hyperactivity disorder (19890261), Attention deficit hyperactivity disorder (19288168), Attention deficit hyperactivity disorder (15719398), Attention deficit hyperactivity disorder (19490304), Attention deficit hyperactivity disorder (18802919), Attention deficit hyperactivity disorder (7717410), Attention deficit hyperactivity disorder (16782077), Attention deficit hyperactivity disorder (18553640), Attention deficit hyperactivity disorder (20049490), Attention deficit hyperactivity disorder (24487615), Attention deficit hyperactivity disorder (17582621), Attention deficit hyperactivity disorder (19506906), Attention deficit hyperactivity disorder (24008096), Bipolar Disorder (17316573), Attention deficit hyperactivity disorder (19409950), Bipolar Disorder (19715710), Attention deficit hyperactivity disorder (19805704), Attention deficit hyperactivity disorder (16178931), Attention deficit hyperactivity disorder (25656223), Attention deficit hyperactivity disorder (26073896), Schizophrenia (18553389), Schizophrenia (11104840), Attention deficit hyperactivity disorder (15724142), Attention deficit hyperactivity disorder (11239904), Attention deficit hyperactivity disorder (17440978), Attention deficit hyperactivity disorder (12898575), Attention deficit hyperactivity disorder (11032386), Schizophrenia (19879111), Attention deficit hyperactivity disorder (16741944), Attention deficit hyperactivity disorder (18023044), Attention deficit hyperactivity disorder (19619618), Attention deficit hyperactivity disorder (21207241), Attention deficit hyperactivity disorder (16861140), Attention deficit hyperactivity disorder (16222334), Attention deficit hyperactivity disorder (20039948), Attention deficit hyperactivity disorder (21216270) |
| ENSG00000157193 | *LRP8* | 3 | Major Depressive Disorder (20493228), Schizophrenia (17936586), Schizophrenia (22419519) |
| ENSG00000214113 | *LYRM4* | 2 | Schizophrenia (21968932), Schizophrenia (23069351) |
| ENSG00000002822 | *MAD1L1* | 2 | Schizophrenia (24556472), Schizophrenia (26528791) |
| ENSG00000151276 | *MAGI1* | 2 | Schizophrenia (22381734), Bipolar Disorder (24215895) |
| ENSG00000166963 | *MAP1A* | 2 | Schizophrenia (19251251), Schizophrenia (19165527) |
| ENSG00000131711 | *MAP1B* | 2 | Schizophrenia (19054571), Attention deficit hyperactivity disorder (26233433) |
| ENSG00000078018 | *MAP2* | 3 | Major Depressive Disorder (15465982), Schizophrenia (11720692), Schizophrenia (19165527) |
| ENSG00000169032 | *MAP2K1* | 2 | Major Depressive Disorder (24709918), Schizophrenia (18319075) |
| ENSG00000171533 | *MAP6* | 7 | Schizophrenia (20417241), Schizophrenia (20856814), Schizophrenia (20336057), Schizophrenia (12231625), Schizophrenia (24704457), Schizophrenia (16624526), Schizophrenia (19165527) |
| ENSG00000102882 | *MAPK3* | 10 | Major Depressive Disorder (25075716), Schizophrenia (18406625), Schizophrenia (25048004), Depressive disorder (20657013), Bipolar Disorder (19407332), Depressive disorder (17712345), Depressive disorder (17889834), Depressive disorder (11331420), Depressive disorder (19424057), Depressive disorder (21368416) |
| ENSG00000116141 | *MARK1* | 7 | Attention deficit hyperactivity disorder (20166940), Autism Spectrum Disorders (18492799), Schizophrenia (22446567), Schizophrenia (14552505), Schizophrenia (20636287), Schizophrenia (12679242), Schizophrenia (12892855) |
| ENSG00000166986 | *MARS* | 2 | Depressive disorder (23377640), Major Depressive Disorder (22647524) |
| ENSG00000134046 | *MBD2* | 3 | Major Depressive Disorder (26047305), Schizophrenia (24849540), Schizophrenia (9154445) |
| ENSG00000204406 | *MBD5* | 6 | Autism Spectrum Disorders (21981781), Bipolar Disorder (23587880), Autism Spectrum Disorders (23632792), Autism Spectrum Disorders (25853262), Autism Spectrum Disorders (23055267), Autism Spectrum Disorders (25271084) |
| ENSG00000185231 | *MC2R* | 4 | Bipolar Disorder (9433545), Schizophrenia (18838498), Major Depressive Disorder (19095219), Bipolar Disorder (7485268) |
| ENSG00000166603 | *MC4R* | 4 | Depressive disorder (18502874), Attention deficit hyperactivity disorder (21211528), Schizophrenia (22310352), Attention deficit hyperactivity disorder (18777518) |
| ENSG00000147316 | *MCPH1* | 4 | Depressive disorder (24975899), Autism Spectrum Disorders (21837366), Bipolar Disorder (23958183), Autism Spectrum Disorders (19793310) |
| ENSG00000140563 | *MCTP2* | 2 | Major Depressive Disorder (18367154), Schizophrenia (19223264) |
| ENSG00000112139 | *MDGA1* | 2 | Schizophrenia (18384059), Bipolar Disorder (21146959) |
| ENSG00000169057 | *MECP2* | 31 | Autism Spectrum Disorders (25299635), Autism Spectrum Disorders (24150225), Autism Spectrum Disorders (19628041), Autism Spectrum Disorders (24448211), Schizophrenia (15211631), Autism Spectrum Disorders (18042715), Autism Spectrum Disorders (23392116), Schizophrenia (19736351), Autism Spectrum Disorders (18631120), Attention deficit hyperactivity disorder (17486179), Major Depressive Disorder (27213019), Autism Spectrum Disorders (20298210), Autism Spectrum Disorders (21372149), Autism Spectrum Disorders (22249109), Autism Spectrum Disorders (19189931), Autism Spectrum Disorders (12555243), Attention deficit hyperactivity disorder (19365833), Autism Spectrum Disorders (24615633), Autism Spectrum Disorders (23226951), Attention deficit hyperactivity disorder (23921973), Autism Spectrum Disorders (19125863), Autism Spectrum Disorders (12027529), Autism Spectrum Disorders (14734626), Attention deficit hyperactivity disorder (18937310), Autism Spectrum Disorders (23169761), Autism Spectrum Disorders (20956852), Depressive disorder (26239616), Autism Spectrum Disorders (23055267), Autism Spectrum Disorders (26808898), Autism Spectrum Disorders (22119903), Autism Spectrum Disorders (21733672) |
| ENSG00000184634 | *MED12* | 10 | Schizophrenia (12860370), Depressive disorder (12216017), Bipolar Disorder (12627458), Schizophrenia (15108174), Schizophrenia (17299734), Schizophrenia (20218787), Depressive disorder (10898921), Schizophrenia (11424983), Schizophrenia (14582143), Schizophrenia (16538184) |
| ENSG00000068305 | *MEF2A* | 2 | Schizophrenia (26421691), Autism Spectrum Disorders (19109909) |
| ENSG00000145794 | *MEGF10* | 2 | Schizophrenia (18179784), Schizophrenia (19721717) |
| ENSG00000105976 | *MET* | 17 | Autism Spectrum Disorders (22958829), Schizophrenia (20080979), Autism Spectrum Disorders (19255034), Autism Spectrum Disorders (21832174), Schizophrenia (15824744), Autism Spectrum Disorders (23097380), Autism Spectrum Disorders (24909855), Autism Spectrum Disorders (19360663), Autism Spectrum Disorders (19548256), Unipolar Depression (15520843), Autism Spectrum Disorders (22833194), Depressive disorder (18347599), Autism Spectrum Disorders (24150225), Autism Spectrum Disorders (17696172), Autism Spectrum Disorders (24240654), Autism Spectrum Disorders (21328570), Autism Spectrum Disorders (19732764) |
| ENSG00000100985 | *MMP9* | 8 | Bipolar Disorder (24402212), Bipolar Disorder (21437990), Bipolar Disorder (19536654), Schizophrenia (20510460), Schizophrenia (25933951), Schizophrenia (20197064), Schizophrenia (19264454), Schizophrenia (25837304) |
| ENSG00000005381 | *MPO* | 2 | Unipolar Depression (18514165), Depressive disorder (20471444) |
| ENSG00000154889 | *MPPE1* | 2 | Bipolar Disorder (19859903), Bipolar Disorder (19328558) |
| ENSG00000175806 | *MSRA* | 2 | Schizophrenia (18506707), Schizophrenia (21679298) |
| ENSG00000100714 | *MTHFD1* | 4 | Schizophrenia (17417062), Bipolar Disorder (19328558), Depressive disorder (26926881), Schizophrenia (21360829) |
| ENSG00000177000 | *MTHFR* | 107 | Schizophrenia (16641680), Major Depressive Disorder (24123968), Schizophrenia (16084002), Unipolar Depression (23900311), Bipolar Disorder (18513846), Schizophrenia (10424670), Schizophrenia (18583979), Schizophrenia (20044984), Bipolar Disorder (17188847), Schizophrenia (24535549), Depressive disorder (23116396), Schizophrenia (20694488), Schizophrenia (9342205), Autism Spectrum Disorders (21069446), Depressive disorder (18801628), Schizophrenia (19939410), Schizophrenia (10889537), Schizophrenia (16172608), Bipolar Disorder (16545905), Unipolar Depression (18165972), Depressive disorder (19650814), Depressive disorder (26177556), Unipolar Depression (26021967), Major Depressive Disorder (23255668), Major Depressive Disorder (16223965), Depressive disorder (24532086), Depressive disorder (26926881), Depressive disorder (12450963), Unipolar Depression (17074966), Bipolar Disorder (25101272), Schizophrenia (24938371), Schizophrenia (19564051), Bipolar Disorder (25744938), Schizophrenia (16076517), Schizophrenia (9451725), Depressive disorder (21635773), Bipolar Disorder (23969624), Depressive disorder (9774778), Schizophrenia (17543893), Schizophrenia (18988738), Schizophrenia (21190096), Bipolar Disorder (21955385), Schizophrenia (19746410), Depressive disorder (24373005), Schizophrenia (17716874), Bipolar Disorder (20552676), Major Depressive Disorder (24751310), Schizophrenia (15289817), Depressive disorder (23586533), Schizophrenia (14499487), Schizophrenia (21334854), Schizophrenia (18715757), Schizophrenia (18165967), Schizophrenia (23318463), Depressive disorder (21772318), Attention deficit hyperactivity disorder (20870238), Autism Spectrum Disorders (19267885), Depressive disorder (12796225), Depressive disorder (19235787), Schizophrenia (24522021), Schizophrenia (17344026), Depressive disorder (19251870), Attention deficit hyperactivity disorder (23227261), Schizophrenia (23353103), Autism Spectrum Disorders (22648721), Bipolar Disorder (18983889), Major Depressive Disorder (25012419), Depressive disorder (26620113), Schizophrenia (20547447), Autism Spectrum Disorders (23653228), Schizophrenia (17503473), Schizophrenia (14572619), Schizophrenia (23076983), Schizophrenia (15806605), Schizophrenia (22128864), Schizophrenia (21302350), Schizophrenia (15564899), Depressive disorder (20163778), Depressive disorder (18981340), Depressive disorder (22739363), Schizophrenia (18186041), Depressive disorder (23285094), Schizophrenia (21980405), Bipolar Disorder (24577139), Schizophrenia (15729744), Schizophrenia (22690662), Depressive disorder (26681493), Schizophrenia (20471108), Depressive disorder (21125200), Schizophrenia (17976958), Schizophrenia (10208443), Schizophrenia (20418067), Major Depressive Disorder (17938638), Bipolar Disorder (19328558), Depressive disorder (22668858), Schizophrenia (21093223), Schizophrenia (27025471), Schizophrenia (21185933), Schizophrenia (16969279), Schizophrenia (20692813), Schizophrenia (22813657), Depressive disorder (11722155), Schizophrenia (23341251), Schizophrenia (18677906), Schizophrenia (22022190), Schizophrenia (24229535), Schizophrenia (19367581) |
| ENSG00000116984 | *MTR* | 5 | Schizophrenia (17417062), Depressive disorder (18801628), Depressive disorder (27111719), Depressive disorder (3425320), Bipolar Disorder (19328558) |
| ENSG00000124275 | *MTRR* | 2 | Bipolar Disorder (17417062), Depressive disorder (23280573) |
| ENSG00000100345 | *MYH9* | 2 | Schizophrenia (20188514), Schizophrenia (18571626) |
| ENSG00000041515 | *MYO16* | 2 | Schizophrenia (24141571), Autism Spectrum Disorders (24132906) |
| ENSG00000167306 | *MYO5B* | 2 | Schizophrenia (23561489), Attention deficit hyperactivity disorder (21276201) |
| ENSG00000099331 | *MYO9B* | 3 | Schizophrenia (21688385), Schizophrenia (17948900), Schizophrenia (25710847) |
| ENSG00000186487 | *MYT1L* | 6 | Schizophrenia (21923761), Unipolar Depression (21048971), Schizophrenia (22547139), Schizophrenia (21990140), Schizophrenia (22157634), Schizophrenia (18940311) |
| ENSG00000102452 | *NALCN* | 2 | Schizophrenia (20674038), Bipolar Disorder (19308021) |
| ENSG00000134265 | *NAPG* | 4 | Bipolar Disorder (17239033), Bipolar Disorder (16395123), Bipolar Disorder (19429185), Bipolar Disorder (19328558) |
| ENSG00000149294 | *NCAM1* | 23 | Bipolar Disorder (15050861), Bipolar Disorder (24651862), Schizophrenia (17413444), Schizophrenia (2136394), Bipolar Disorder (21515372), Bipolar Disorder (17239033), Schizophrenia (19411161), Depressive disorder (18973581), Schizophrenia (17161382), Major Depressive Disorder (18068248), Schizophrenia (18601968), Schizophrenia (24057454), Schizophrenia (25445624), Schizophrenia (26460482), Depressive disorder (22361116), Bipolar Disorder (11353453), Bipolar Disorder (19328558), Schizophrenia (11681838), Depressive disorder (14985425), Schizophrenia (9154219), Attention deficit hyperactivity disorder (21606926), Attention deficit hyperactivity disorder (22832519), Depressive disorder (17409734) |
| ENSG00000130287 | *NCAN* | 10 | Bipolar Disorder (23712748), Bipolar Disorder (25220293), Bipolar Disorder (24955366), Bipolar Disorder (21353194), Bipolar Disorder (23198940), Major Depressive Disorder (25801500), Schizophrenia (22497794), Schizophrenia (23795679), Schizophrenia (20950796), Bipolar Disorder (22952076) |
| ENSG00000198646 | *NCOA6* | 2 | Autism Spectrum Disorders (17236127), Schizophrenia (6685980) |
| ENSG00000166579 | *NDEL1* | 11 | Schizophrenia (19632097), Schizophrenia (20084519), Schizophrenia (17258902), Schizophrenia (19251251), Schizophrenia (24940743), Schizophrenia (17482883), Schizophrenia (18469341), Schizophrenia (22099459), Schizophrenia (21195721), Schizophrenia (26851141), Schizophrenia (21998303) |
| ENSG00000023228 | *NDUFS1* | 5 | Schizophrenia (16788776), Bipolar Disorder (26093828), Schizophrenia (25354934), Autism Spectrum Disorders (19894076), Schizophrenia (24196945) |
| ENSG00000104722 | *NEFM* | 3 | Schizophrenia (19034380), Depressive disorder (19204725), Schizophrenia (24636402) |
| ENSG00000173848 | *NET1* | 5 | Attention deficit hyperactivity disorder (15717291), Attention deficit hyperactivity disorder (20039948), Attention deficit hyperactivity disorder (24942521), Attention deficit hyperactivity disorder (17994190), Attention deficit hyperactivity disorder (15719398) |
| ENSG00000186575 | *NF2* | 2 | Schizophrenia (17012685), Attention deficit hyperactivity disorder (20610137) |
| ENSG00000165030 | *NFIL3* | 2 | Attention deficit hyperactivity disorder (18937294), Schizophrenia (19839995) |
| ENSG00000109320 | *NFKB1* | 41 | Schizophrenia (19961902), Schizophrenia (12685995), Schizophrenia (16843094), Bipolar Disorder (17224092), Schizophrenia (8873294), Schizophrenia (9012828), Schizophrenia (18762587), Schizophrenia (20102668), Schizophrenia (17604606), Schizophrenia (14569275), Schizophrenia (20363872), Schizophrenia (1450287), Schizophrenia (17012698), Schizophrenia (22048129), Schizophrenia (15625200), Schizophrenia (10394470), Schizophrenia (17349863), Schizophrenia (16469942), Schizophrenia (15465979), Schizophrenia (12450949), Schizophrenia (16876141), Schizophrenia (15100704), Schizophrenia (25464917), Bipolar Disorder (18189279), Schizophrenia (8094267), Schizophrenia (17475740), Schizophrenia (8399828), Major Depressive Disorder (25734512), Schizophrenia (18056246), Schizophrenia (23318559), Schizophrenia (23910792), Schizophrenia (15038993), Schizophrenia (18545269), Schizophrenia (12399955), Schizophrenia (17192894), Schizophrenia (20382002), Bipolar Disorder (16823804), Schizophrenia (26441157), Schizophrenia (17014995), Schizophrenia (17448448), Schizophrenia (10618013) |
| ENSG00000134259 | *NGF* | 8 | Autism Spectrum Disorders (22105621), Attention deficit hyperactivity disorder (17192954), Depressive disorder (15940299), Unipolar Depression (26021968), Attention deficit hyperactivity disorder (18179783), Depressive disorder (23020178), Depressive disorder (14699963), Schizophrenia (7786881) |
| ENSG00000064300 | *NGFR* | 7 | Depressive disorder (15274039), Schizophrenia (18470533), Major Depressive Disorder (26278479), Autism Spectrum Disorders (24736721), Major Depressive Disorder (18081157), Depressive disorder (15883381), Attention deficit hyperactivity disorder (18428117) |
| ENSG00000169760 | *NLGN1* | 5 | Schizophrenia (26674772), Schizophrenia (21915259), Autism Spectrum Disorders (22952857), Schizophrenia (19736351), Autism Spectrum Disorders (16077734) |
| ENSG00000196338 | *NLGN3* | 12 | Autism Spectrum Disorders (24773431), Autism Spectrum Disorders (22892527), Autism Spectrum Disorders (24995974), Autism Spectrum Disorders (15152050), Autism Spectrum Disorders (21364653), Autism Spectrum Disorders (25299583), Autism Spectrum Disorders (21569590), Autism Spectrum Disorders (16077734), Autism Spectrum Disorders (12669065), Autism Spectrum Disorders (16508939), Autism Spectrum Disorders (18189281), Autism Spectrum Disorders (18555979) |
| ENSG00000089250 | *NOS1* | 39 | Major Depressive Disorder (26812915), Schizophrenia (12140778), Schizophrenia (18544180), Schizophrenia (19513863), Depressive disorder (22959237), Schizophrenia (20645313), Autism Spectrum Disorders (17893705), Attention deficit hyperactivity disorder (21724667), Depressive disorder (20052609), Attention deficit hyperactivity disorder (26233433), Schizophrenia (16380905), Depressive disorder (24917196), Schizophrenia (24220657), Bipolar Disorder (19285348), Autism Spectrum Disorders (18563708), Unipolar Depression (25612209), Schizophrenia (20921115), Schizophrenia (20605417), Attention deficit hyperactivity disorder (22939004), Depressive disorder (17854383), Bipolar Disorder (16389274), Schizophrenia (15094474), Attention deficit hyperactivity disorder (16082698), Schizophrenia (25490993), Schizophrenia (19805695), Bipolar Disorder (19328558), Bipolar Disorder (25939888), Schizophrenia (26440917), Schizophrenia (14623375), Schizophrenia (21281558), Depressive disorder (22989585), Autism Spectrum Disorders (22892527), Schizophrenia (7676834), Schizophrenia (18923069), Attention deficit hyperactivity disorder (26821215), Schizophrenia (21620982), Schizophrenia (22227051), Schizophrenia (21520349), Schizophrenia (19844207) |
| ENSG00000164867 | *NOS3* | 9 | Unipolar Depression (15967063), Schizophrenia (16495774), Depressive disorder (20163778), Depressive disorder (14613853), Schizophrenia (24346810), Depressive disorder (22031268), Depressive disorder (22989585), Schizophrenia (20691427), Bipolar Disorder (25939888) |
| ENSG00000187258 | *NPSR1* | 5 | Schizophrenia (17669576), Attention deficit hyperactivity disorder (23325374), Schizophrenia (22078257), Depressive disorder (20171785), Schizophrenia (23680103) |
| ENSG00000164128 | *NPY1R* | 5 | Depressive disorder (19623606), Depressive disorder (21803058), Depressive disorder (9729278), Schizophrenia (14741323), Schizophrenia (21512575) |
| ENSG00000181019 | *NQO1* | 6 | Schizophrenia (19778569), Schizophrenia (18977034), Schizophrenia (23360829), Schizophrenia (15151706), Schizophrenia (12834817), Major Depressive Disorder (17644186) |
| ENSG00000124588 | *NQO2* | 2 | Schizophrenia (14639047), Schizophrenia (20861374) |
| ENSG00000126368 | *NR1D1* | 12 | Bipolar Disorder (21835597), Bipolar Disorder (19839995), Depressive disorder (25359533), Bipolar Disorder (22538398), Bipolar Disorder (25789810), Bipolar Disorder (18228528), Bipolar Disorder (20072116), Bipolar Disorder (21781277), Bipolar Disorder (19818381), Bipolar Disorder (19267705), Bipolar Disorder (24389266), Bipolar Disorder (20348464) |
| ENSG00000151623 | *NR3C2* | 18 | Unipolar Depression (21036197), Major Depressive Disorder (21195417), Autism Spectrum Disorders (25912394), Major Depressive Disorder (25765757), Depressive disorder (24485490), Major Depressive Disorder (17360585), Major Depressive Disorder (19906241), Depressive disorder (25497375), Bipolar Disorder (21531081), Depressive disorder (22832354), Depressive disorder (21164267), Depressive disorder (20528958), Depressive disorder (26096463), Depressive disorder (11566214), Depressive disorder (10903817), Attention deficit hyperactivity disorder (22584804), Depressive disorder (24690014), Schizophrenia (19850283) |
| ENSG00000153234 | *NR4A2* | 16 | Major Depressive Disorder (16631355), Schizophrenia (25982322), Schizophrenia (22294735), Schizophrenia (12815740), Attention deficit hyperactivity disorder (15635701), Schizophrenia (18583979), Schizophrenia (11803525), Bipolar Disorder (11121187), Bipolar Disorder (20659174), Schizophrenia (21545404), Schizophrenia (12627459), Schizophrenia (15211629), Schizophrenia (10216261), Schizophrenia (18655117), Schizophrenia (17457314), Attention deficit hyperactivity disorder (19352218) |
| ENSG00000091129 | *NRCAM* | 2 | Schizophrenia (26674772), Schizophrenia (19154219) |
| ENSG00000157168 | *NRG1* | 228 | Schizophrenia (17609743), Schizophrenia (12808428), Schizophrenia (17336946), Schizophrenia (16249994), Schizophrenia (19782967), Schizophrenia (14729827), Schizophrenia (18466881), Schizophrenia (22019858), Schizophrenia (16219118), Recurrent major depressive episodes (19367584), Schizophrenia (19367581), Schizophrenia (21907759), Schizophrenia (16958035), Schizophrenia (19652122), Schizophrenia (18585932), Schizophrenia (20218788), Schizophrenia (16526041), Bipolar Disorder (19184335), Schizophrenia (12874607), Schizophrenia (20526724), Schizophrenia (25106628), Schizophrenia (12478479), Schizophrenia (19913623), Schizophrenia (20435087), Schizophrenia (15303101), Schizophrenia (20036336), Schizophrenia (24683514), Schizophrenia (21295966), Schizophrenia (17333138), Bipolar Disorder (18234478), Schizophrenia (18520162), Schizophrenia (20212127), Schizophrenia (18282690), Schizophrenia (20638435), Schizophrenia (22467496), Schizophrenia (20433909), Schizophrenia (26888291), Schizophrenia (15000348), Schizophrenia (18180429), Schizophrenia (21993442), Schizophrenia (18571900), Schizophrenia (15007393), Bipolar Disorder (23273899), Schizophrenia (19765633), Schizophrenia (17033632), Schizophrenia (18606232), Schizophrenia (19336245), Schizophrenia (20497232), Schizophrenia (19448847), Schizophrenia (16326006), Schizophrenia (15538186), Schizophrenia (18032396), Schizophrenia (16612196), Schizophrenia (15744031), Schizophrenia (19937977), Schizophrenia (12145742), Schizophrenia (17565985), Schizophrenia (23449491), Schizophrenia (17901998), Schizophrenia (16442083), Schizophrenia (17092693), Schizophrenia (18159252), Bipolar Disorder (19967439), Schizophrenia (16618933), Schizophrenia (15545978), Schizophrenia (16767099), Schizophrenia (20921115), Schizophrenia (18455303), Schizophrenia (16319375), Schizophrenia (15670788), Schizophrenia (22832403), Bipolar Disorder (20374152), Schizophrenia (17460065), Schizophrenia (17551090), Schizophrenia (23489597), Schizophrenia (25142529), Schizophrenia (18193072), Schizophrenia (18583979), Schizophrenia (21512575), Schizophrenia (27236031), Schizophrenia (17366345), Schizophrenia (22777684), Schizophrenia (17405926), Schizophrenia (18668031), Schizophrenia (16687441), Bipolar Disorder (15939841), Bipolar Disorder (18466879), Schizophrenia (18996920), Schizophrenia (20688137), Schizophrenia (16287046), Schizophrenia (25967537), Schizophrenia (15248869), Schizophrenia (17300918), Schizophrenia (16520822), Schizophrenia (16219117), Schizophrenia (16361955), Schizophrenia (18186075), Schizophrenia (16603473), Schizophrenia (19054571), Schizophrenia (19046988), Schizophrenia (16730337), Schizophrenia (24380930), Schizophrenia (16638076), Bipolar Disorder (18474212), Schizophrenia (15197397), Schizophrenia (24237343), Schizophrenia (12952866), Schizophrenia (17152404), Schizophrenia (17408693), Depressive disorder (19204725), Bipolar Disorder (16940976), Schizophrenia (21745728), Schizophrenia (26534905), Schizophrenia (21688113), Schizophrenia (21513767), Schizophrenia (19439994), Schizophrenia (19118813), Bipolar Disorder (24767010), Schizophrenia (17905522), Schizophrenia (19362450), Schizophrenia (23719163), Schizophrenia (24636039), Schizophrenia (21637803), Schizophrenia (17072305), Schizophrenia (19350564), Schizophrenia (23032943), Schizophrenia (17982252), Schizophrenia (18497102), Schizophrenia (23389757), Schizophrenia (19127563), Schizophrenia (19736351), Schizophrenia (21035784), Bipolar Disorder (14677079), Schizophrenia (20582876), Schizophrenia (21371516), Schizophrenia (20926259), Schizophrenia (16082692), Schizophrenia (15162166), Schizophrenia (19058791), Bipolar Disorder (24865593), Schizophrenia (20371257), Schizophrenia (18543275), Schizophrenia (20561508), Schizophrenia (19965935), Bipolar Disorder (19339916), Schizophrenia (17519028), Schizophrenia (23633123), Schizophrenia (18516516), Schizophrenia (20061032), Schizophrenia (21234898), Schizophrenia (16483744), Schizophrenia (17598910), Bipolar Disorder (19328558), Schizophrenia (19553869), Schizophrenia (20713722), Schizophrenia (19143653), Schizophrenia (18494263), Schizophrenia (18584117), Schizophrenia (19449332), Schizophrenia (17440436), Schizophrenia (21557953), Schizophrenia (19575259), Schizophrenia (17579610), Schizophrenia (20978455), Schizophrenia (25529856), Schizophrenia (22832904), Schizophrenia (16640117), Schizophrenia (18455369), Schizophrenia (19521112), Schizophrenia (17631867), Schizophrenia (23360725), Schizophrenia (17185511), Schizophrenia (15276238), Schizophrenia (24935406), Schizophrenia (17884806), Schizophrenia (16189508), Schizophrenia (18291420), Depressive disorder (17449450), Schizophrenia (19199244), Schizophrenia (14966480), Bipolar Disorder (23022220), Schizophrenia (25858800), Schizophrenia (26909665), Schizophrenia (16402353), Schizophrenia (15219675), Schizophrenia (22378022), Schizophrenia (21858616), Schizophrenia (12573388), Schizophrenia (17503451), Schizophrenia (22520967), Schizophrenia (24299109), Schizophrenia (20701826), Schizophrenia (22689948), Schizophrenia (18704261), Schizophrenia (19394386), Schizophrenia (16081509), Schizophrenia (18470533), Bipolar Disorder (21232925), Schizophrenia (17164265), Schizophrenia (25579050), Schizophrenia (18478032), Schizophrenia (22771711), Schizophrenia (17349866), Schizophrenia (14569272), Schizophrenia (25754081), Schizophrenia (24622944), Schizophrenia (18806920), Schizophrenia (23301017), Schizophrenia (16891421), Schizophrenia (17485733), Schizophrenia (22253750), Schizophrenia (21127983), Schizophrenia (25897834), Schizophrenia (18182443), Schizophrenia (22183611), Bipolar Disorder (14623361), Schizophrenia (20182055), Schizophrenia (18286587), Schizophrenia (17275115), Schizophrenia (17410640), Schizophrenia (21876540), Schizophrenia (18985292), Bipolar Disorder (24633675), Schizophrenia (15704228), Schizophrenia (19545856), Schizophrenia (18243664), Schizophrenia (22209534), Schizophrenia (25349163) |
| ENSG00000185737 | *NRG3* | 11 | Attention Deficit Disorder (22831755), Schizophrenia (20713722), Schizophrenia (19118813), Schizophrenia (18708184), Schizophrenia (21762460), Schizophrenia (20548296), Attention deficit hyperactivity disorder (18846501), Bipolar Disorder (19765633), Schizophrenia (17598910), Schizophrenia (22981155), Schizophrenia (24431462) |
| ENSG00000124785 | *NRN1* | 3 | Bipolar Disorder (26700405), Schizophrenia (24350851), Schizophrenia (19569075) |
| ENSG00000099250 | *NRP1* | 4 | Depressive disorder (23261523), Schizophrenia (15274031), Attention deficit hyperactivity disorder (25346392), Schizophrenia (19054571) |
| ENSG00000152954 | *NRSN1* | 2 | Major Depressive Disorder (10321984), Attention deficit hyperactivity disorder (19362708) |
| ENSG00000185652 | *NTF3* | 26 | Schizophrenia (9149322), Attention Deficit Disorder (20576502), Unipolar Depression (18313696), Attention deficit hyperactivity disorder (18428117), Schizophrenia (8837968), Schizophrenia (8925252), Schizophrenia (7733919), Schizophrenia (16979250), Depressive disorder (21218562), Schizophrenia (9197906), Major Depressive Disorder (25858873), Schizophrenia (11920853), Schizophrenia (15365216), Attention deficit hyperactivity disorder (18179783), Schizophrenia (9588763), Unipolar Depression (24577123), Attention deficit hyperactivity disorder (23471121), Schizophrenia (8085468), Schizophrenia (15003293), Schizophrenia (8837975), Schizophrenia (15474906), Attention deficit hyperactivity disorder (17192954), Bipolar Disorder (22484201), Schizophrenia (11343865), Schizophrenia (18572319), Depressive disorder (20726825) |
| ENSG00000162631 | *NTNG1* | 7 | Schizophrenia (15508520), Schizophrenia (25325217), Schizophrenia (15705354), Bipolar Disorder (17507910), Schizophrenia (18384956), Bipolar Disorder (20079890), Schizophrenia (21641949) |
| ENSG00000196358 | *NTNG2* | 6 | Bipolar Disorder (17507910), Schizophrenia (15508520), Schizophrenia (21641949), Schizophrenia (18384956), Schizophrenia (20079890), Schizophrenia (15705354) |
| ENSG00000198400 | *NTRK1* | 7 | Attention deficit hyperactivity disorder (25346392), Schizophrenia (19435634), Schizophrenia (9865928), Bipolar Disorder (14708030), Schizophrenia (21223646), Schizophrenia (21317683), Depressive disorder (20014955) |
| ENSG00000133636 | *NTS* | 12 | Depressive disorder (22754041), Schizophrenia (25449842), Schizophrenia (15585443), Bipolar Disorder (10909129), Schizophrenia (21124211), Schizophrenia (23483448), Schizophrenia (10822351), Schizophrenia (20193696), Schizophrenia (20659557), Schizophrenia (11032391), Schizophrenia (10435199), Schizophrenia (11743941) |
| ENSG00000114026 | *OGG1* | 2 | Depressive disorder (16949154), Schizophrenia (18384956) |
| ENSG00000082556 | *OPRK1* | 7 | Depressive disorder (25816912), Schizophrenia (11576758), Depressive disorder (19924112), Depressive disorder (17601558), Depressive disorder (25286107), Depressive disorder (23293139), Depressive disorder (23101464) |
| ENSG00000180914 | *OXTR* | 26 | Autism Spectrum Disorders (25092245), Attention deficit hyperactivity disorder (26174935), Schizophrenia (22651577), Schizophrenia (23284802), Unipolar Depression (21208749), Depressive disorder (19015103), Autism Spectrum Disorders (25687563), Autism Spectrum Disorders (23684879), Autism Spectrum Disorders (20094064), Schizophrenia (20196918), Autism Spectrum Disorders (20832055), Autism Spectrum Disorders (18207134), Autism Spectrum Disorders (24814480), Depressive disorder (19515497), Autism Spectrum Disorders (19777562), Depressive disorder (25622005), Depressive disorder (25262417), Depressive disorder (22510359), Schizophrenia (25244972), Depressive disorder (24703166), Autism Spectrum Disorders (27015428), Depressive disorder (20708845), Autism Spectrum Disorders (26365303), Autism Spectrum Disorders (17893705), Autism Spectrum Disorders (19376182), Autism Spectrum Disorders (24836510) |
| ENSG00000076641 | *PAG1* | 2 | Schizophrenia (25005592), Schizophrenia (21822266) |
| ENSG00000171759 | *PAH* | 8 | Schizophrenia (19268543), Schizophrenia (24606907), Schizophrenia (22414661), Schizophrenia (8357034), Bipolar Disorder (12782966), Schizophrenia (18937293), Autism Spectrum Disorders (23352163), Schizophrenia (12210276) |
| ENSG00000180370 | *PAK2* | 2 | Schizophrenia (20691406), Schizophrenia (21850710) |
| ENSG00000177425 | *PAWR* | 4 | Major Depressive Disorder (20735158), Schizophrenia (18281137), Bipolar Disorder (18085546), Depressive disorder (21596067) |
| ENSG00000163939 | *PBRM1* | 4 | Bipolar Disorder (21217634), Schizophrenia (23967141), Schizophrenia (21037240), Bipolar Disorder (22560537) |
| ENSG00000078674 | *PCM1* | 7 | Schizophrenia (19048012), Schizophrenia (16894060), Schizophrenia (20360304), Schizophrenia (21195721), Schizophrenia (18762586), Schizophrenia (20468070), Schizophrenia (21481569) |
| ENSG00000065989 | *PDE4A* | 11 | Depressive disorder (17700644), Major Depressive Disorder (23129425), Depressive disorder (17481393), Schizophrenia (18998737), Major Depressive Disorder (22677471), Depressive disorder (18060387), Depressive disorder (18090323), Depressive disorder (23003922), Bipolar Disorder (19018233), Depressive disorder (22487514), Schizophrenia (21898905) |
| ENSG00000184588 | *PDE4B* | 24 | Schizophrenia (20436352), Schizophrenia (16293762), Schizophrenia (26756575), Schizophrenia (25546171), Schizophrenia (17258902), Depressive disorder (17823207), Schizophrenia (18090323), Bipolar Disorder (18394866), Depressive disorder (21184794), Schizophrenia (19632097), Depressive disorder (17481393), Schizophrenia (17417055), Major Depressive Disorder (18785206), Schizophrenia (19350560), Schizophrenia (21876540), Schizophrenia (22160351), Schizophrenia (19251251), Depressive disorder (21376063), Schizophrenia (21195721), Schizophrenia (18329668), Depressive disorder (21458469), Schizophrenia (25926551), Schizophrenia (18829870), Schizophrenia (16814262) |
| ENSG00000113448 | *PDE4D* | 5 | Depressive disorder (12377395), Depressive disorder (22487514), Schizophrenia (19251251), Depressive disorder (23003922), Attention deficit hyperactivity disorder (20308990) |
| ENSG00000171408 | *PDE7B* | 4 | Schizophrenia (19850283), Schizophrenia (20371615), Schizophrenia (27092952), Schizophrenia (20071346) |
| ENSG00000160191 | *PDE9A* | 3 | Major Depressive Disorder (23319000), Major Depressive Disorder (17008408), Bipolar Disorder (10950928) |
| ENSG00000175087 | *PDIK1L* | 2 | Schizophrenia (22367616), Schizophrenia (23290496) |
| ENSG00000163110 | *PDLIM5* | 20 | Major Depressive Disorder (18197271), Bipolar Disorder (16044170), Bipolar Disorder (18496208), Schizophrenia (24064681), Major Depressive Disorder (16595163), Schizophrenia (17287082), Bipolar Disorder (19448850), Schizophrenia (18021463), Schizophrenia (16213469), Bipolar Disorder (14743183), Unipolar Depression (23593136), Bipolar Disorder (18456508), Bipolar Disorder (18496210), Schizophrenia (15362566), Bipolar Disorder (19328558), Bipolar Disorder (12782966), Bipolar Disorder (23031404), Bipolar Disorder (24554194), Single major depressive episode (24620999), Bipolar Disorder (17239033) |
| ENSG00000101327 | *PDYN* | 10 | Major Depressive Disorder (24231353), Unipolar Depression (19116947), Bipolar Disorder (11803449), Schizophrenia (12207142), Depressive disorder (23293139), Depressive disorder (24816773), Depressive disorder (23101464), Bipolar Disorder (11576758), Schizophrenia (15301734), Depressive disorder (23904614) |
| ENSG00000162734 | *PEA15* | 2 | Unipolar Depression (25075716), Schizophrenia (20537721) |
| ENSG00000133027 | *PEMT* | 2 | Schizophrenia (17720317), Schizophrenia (19647326) |
| ENSG00000181195 | *PENK* | 5 | Bipolar Disorder (10893493), Depressive disorder (24804898), Depressive disorder (16095981), Depressive disorder (16547969), Depressive disorder (17375141) |
| ENSG00000179094 | *PER1* | 6 | Bipolar Disorder (20072116), Unipolar Depression (26690871), Schizophrenia (19839995), Attention deficit hyperactivity disorder (18821565), Depressive disorder (25359533), Bipolar Disorder (18228528) |
| ENSG00000141959 | *PFKL* | 5 | Bipolar Disorder (7573177), Bipolar Disorder (11304836), Bipolar Disorder (9915960), Bipolar Disorder (9119364), Bipolar Disorder (8988454) |
| ENSG00000096088 | *PGC* | 9 | Schizophrenia (25963331), Schizophrenia (22344220), Schizophrenia (25579050), Schizophrenia (25869805), Schizophrenia (26523775), Depressive disorder (25259918), Schizophrenia (24284030), Bipolar Disorder (26178159), Schizophrenia (27023174) |
| ENSG00000109132 | *PHOX2B* | 3 | Schizophrenia (16021468), Schizophrenia (21881099), Schizophrenia (14709596) |
| ENSG00000078142 | *PIK3C3* | 5 | Schizophrenia (15121481), Schizophrenia (18420347), Schizophrenia (19451863), Schizophrenia (18077426), Schizophrenia (15814194) |
| ENSG00000121879 | *PIK3CA* | 10 | Autism Spectrum Disorders (19545994), Schizophrenia (26643470), Autism Spectrum Disorders (26770665), Schizophrenia (22209534), Autism Spectrum Disorders (21837366), Schizophrenia (22771711), Attention deficit hyperactivity disorder (19458356), Bipolar Disorder (19196980), Schizophrenia (22689948), Schizophrenia (20691427) |
| ENSG00000171608 | *PIK3CD* | 6 | Schizophrenia (22209534), Autism Spectrum Disorders (26770665), Autism Spectrum Disorders (19545994), Schizophrenia (22689948), Attention Deficit Disorder (19458356), Schizophrenia (22771711) |
| ENSG00000105851 | *PIK3CG* | 6 | Schizophrenia (22771711), Autism Spectrum Disorders (26770665), Attention Deficit Disorder (19458356), Schizophrenia (22209534), Schizophrenia (22689948), Autism Spectrum Disorders (19545994) |
| ENSG00000164093 | *PITX2* | 5 | Schizophrenia (18262772), Schizophrenia (26263491), Autism Spectrum Disorders (14985297), Autism Spectrum Disorders (22569110), Autism Spectrum Disorders (20881294) |
| ENSG00000107859 | *PITX3* | 3 | Schizophrenia (20570600), Depressive disorder (24480473), Autism Spectrum Disorders (24555714) |
| ENSG00000116711 | *PLA2G4A* | 22 | Schizophrenia (8888134), Schizophrenia (9800222), Schizophrenia (11353443), Bipolar Disorder (20038946), Schizophrenia (18583979), Schizophrenia (16181776), Schizophrenia (17417066), Schizophrenia (11807411), Schizophrenia (19560328), Bipolar Disorder (15118355), Schizophrenia (17629734), Schizophrenia (9323323), Schizophrenia (15041036), Schizophrenia (15276701), Schizophrenia (18562188), Schizophrenia (15999343), Schizophrenia (15519500), Schizophrenia (15318030), Bipolar Disorder (12895207), Schizophrenia (12759552), Schizophrenia (8873778), Schizophrenia (9689723) |
| ENSG00000184381 | *PLA2G6* | 9 | Schizophrenia (18562188), Depressive disorder (22580182), Schizophrenia (26160611), Bipolar Disorder (23277130), Schizophrenia (15318030), Schizophrenia (16115752), Schizophrenia (15999343), Schizophrenia (17629734), Schizophrenia (12759552) |
| ENSG00000146070 | *PLA2G7* | 4 | Schizophrenia (19892409), Schizophrenia (11850055), Schizophrenia (15318030), Schizophrenia (9434759) |
| ENSG00000163803 | *PLB1* | 3 | Schizophrenia (17629734), Depressive disorder (20034614), Schizophrenia (9689723) |
| ENSG00000124181 | *PLCG1* | 2 | Bipolar Disorder (11409699), Bipolar Disorder (11353454) |
| ENSG00000123560 | *PLP1* | 8 | Schizophrenia (15694262), Schizophrenia (18604471), Schizophrenia (19875103), Schizophrenia (15334603), Major Depressive Disorder (17291372), Schizophrenia (16641098), Major Depressive Disorder (25930075), Schizophrenia (19571127) |
| ENSG00000076356 | *PLXNA2* | 8 | Schizophrenia (19367581), Schizophrenia (18583979), Schizophrenia (16402134), Schizophrenia (17346868), Schizophrenia (18065206), Schizophrenia (20684831), Schizophrenia (18096369), Schizophrenia (17339520) |
| ENSG00000183395 | *PMCH* | 2 | Bipolar Disorder (7712114), Depressive disorder (16934771) |
| ENSG00000140521 | *POLG* | 2 | Bipolar Disorder (21866111), Bipolar Disorder (20392297) |
| ENSG00000115138 | *POMC* | 16 | Bipolar Disorder (2843795), Unipolar Depression (25448875), Attention deficit hyperactivity disorder (18713819), Schizophrenia (18291626), Depressive disorder (24138845), Bipolar Disorder (2984925), Depressive disorder (15239795), Schizophrenia (19394958), Depressive disorder (18201294), Bipolar Disorder (9664777), Depressive disorder (22475622), Schizophrenia (19428782), Schizophrenia (18155448), Schizophrenia (12643442), Depressive disorder (12818721), Major Depressive Disorder (15504420) |
| ENSG00000186951 | *PPARA* | 4 | Schizophrenia (25433960), Schizophrenia (25087592), Schizophrenia (11840500), Schizophrenia (22920733) |
| ENSG00000112033 | *PPARD* | 2 | Schizophrenia (18797401), Bipolar Disorder (18606951) |
| ENSG00000132170 | *PPARG* | 3 | Schizophrenia (19560328), Schizophrenia (19193342), Schizophrenia (19622037) |
| ENSG00000109819 | *PPARGC1A* | 3 | Schizophrenia (23348010), Depressive disorder (21630437), Schizophrenia (26663532) |
| ENSG00000138398 | *PPIG* | 2 | Depressive disorder (22832401), Schizophrenia (21142916) |
| ENSG00000131771 | *PPP1R1B* | 17 | Schizophrenia (21453742), Bipolar Disorder (17521792), Schizophrenia (17618027), Schizophrenia (20483474), Bipolar Disorder (20874815), Schizophrenia (18055181), Schizophrenia (19463699), Schizophrenia (24704945), Schizophrenia (22179181), Bipolar Disorder (23295814), Depressive disorder (19834457), Attention deficit hyperactivity disorder (17948899), Bipolar Disorder (26872373), Bipolar Disorder (18573638), Schizophrenia (16750903), Schizophrenia (17290303), Schizophrenia (22576830) |
| ENSG00000156475 | *PPP2R2B* | 3 | Schizophrenia (12497613), Schizophrenia (18484086), Depressive disorder (21827912) |
| ENSG00000074211 | *PPP2R2C* | 2 | Bipolar Disorder (20308991), Bipolar Disorder (24387768) |
| ENSG00000120910 | *PPP3CC* | 14 | Schizophrenia (19367581), Bipolar Disorder (19204725), Schizophrenia (18343007), Schizophrenia (15843870), Schizophrenia (20537399), Schizophrenia (18991849), Schizophrenia (17339875), Schizophrenia (23497497), Schizophrenia (17895921), Schizophrenia (17185513), Schizophrenia (12851458), Schizophrenia (15820226), Schizophrenia (18715757), Schizophrenia (24399042) |
| ENSG00000131238 | *PPT1* | 3 | Schizophrenia (19165527), Schizophrenia (22155432), Schizophrenia (19054571) |
| ENSG00000085377 | *PREP* | 2 | Depressive disorder (15911340), Bipolar Disorder (17324276) |
| ENSG00000175785 | *PRIMA1* | 2 | Unipolar Depression (22511943), Bipolar Disorder (24389572) |
| ENSG00000154229 | *PRKCA* | 3 | Schizophrenia (19786960), Schizophrenia (21281445), Schizophrenia (18281137) |
| ENSG00000166501 | *PRKCB* | 3 | Major Depressive Disorder (20092830), Major Depressive Disorder (26343587), Autism Spectrum Disorders (25313507) |
| ENSG00000163558 | *PRKCI* | 4 | Depressive disorder (22485133), Bipolar Disorder (19912621), Bipolar Disorder (21382426), Bipolar Disorder (26563126) |
| ENSG00000101292 | *PROKR2* | 2 | Bipolar Disorder (20576534), Unipolar Depression (19544013) |
| ENSG00000112812 | *PRSS16* | 2 | Schizophrenia (22488895), Schizophrenia (22384243) |
| ENSG00000059915 | *PSD* | 7 | Schizophrenia (16122832), Schizophrenia (18033238), Schizophrenia (16702973), Schizophrenia (16023328), Schizophrenia (23936182), Schizophrenia (16762023), Autism Spectrum Disorders (23650259) |
| ENSG00000080815 | *PSEN1* | 9 | Depressive disorder (14966176), Schizophrenia (19232479), Depressive disorder (10904126), Attention Deficit Disorder (15272895), Depressive disorder (11567612), Depressive disorder (20052609), Depressive disorder (21373759), Attention Deficit Disorder (12925374), Depressive disorder (22045496) |
| ENSG00000143801 | *PSEN2* | 5 | Schizophrenia (23335491), Schizophrenia (19232479), Bipolar Disorder (15306129), Attention Deficit Disorder (12925374), Bipolar Disorder (20347905) |
| ENSG00000183527 | *PSMG1* | 2 | Schizophrenia (19958095), Schizophrenia (17387318) |
| ENSG00000165186 | *PTCHD1* | 7 | Autism Spectrum Disorders (20844286), Autism Spectrum Disorders (18252227), Autism Spectrum Disorders (25782667), Attention deficit hyperactivity disorder (27007844), Autism Spectrum Disorders (25131214), Autism Spectrum Disorders (21439084), Autism Spectrum Disorders (22228009) |
| ENSG00000171862 | *PTEN* | 16 | Autism Spectrum Disorders (25916396), Attention deficit hyperactivity disorder (19458356), Autism Spectrum Disorders (25647146), Autism Spectrum Disorders (15805158), Autism Spectrum Disorders (19545994), Attention Deficit Disorder (23660947), Autism Spectrum Disorders (26827793), Autism Spectrum Disorders (17427195), Autism Spectrum Disorders (23695273), Autism Spectrum Disorders (25937288), Autism Spectrum Disorders (22664040), Autism Spectrum Disorders (26843422), Autism Spectrum Disorders (25288137), Autism Spectrum Disorders (20533527), Autism Spectrum Disorders (19265751), Autism Spectrum Disorders (21828076) |
| ENSG00000095303 | *PTGS1* | 3 | Schizophrenia (16181776), Depressive disorder (15776109), Schizophrenia (22397921) |
| ENSG00000073756 | *PTGS2* | 15 | Depressive disorder (21655952), Depressive disorder (20588070), Depressive disorder (19356723), Unipolar Depression (21168919), Depressive disorder (22331023), Major Depressive Disorder (20034614), Depressive disorder (12397273), Depressive disorder (24388097), Schizophrenia (15519500), Depressive disorder (22580182), Schizophrenia (19560328), Schizophrenia (15041036), Schizophrenia (15276698), Schizophrenia (22397921), Schizophrenia (16181776) |
| ENSG00000110786 | *PTPN5* | 5 | Schizophrenia (25956630), Bipolar Disorder (25129258), Bipolar Disorder (20872766), Schizophrenia (22555153), Schizophrenia (22781170) |
| ENSG00000132670 | *PTPRA* | 2 | Schizophrenia (25393624), Schizophrenia (21831360) |
| ENSG00000115828 | *QPCT* | 2 | Schizophrenia (26492838), Bipolar Disorder (17097106) |
| ENSG00000154710 | *RABGEF1* | 2 | Bipolar Disorder (20436929), Depressive disorder (16754837) |
| ENSG00000136238 | *RAC1* | 5 | Schizophrenia (25956630), Depressive disorder (23416703), Schizophrenia (16402129), Schizophrenia (23720743), Depressive disorder (17448908) |
| ENSG00000131759 | *RARA* | 4 | Schizophrenia (24564241), Schizophrenia (15698673), Schizophrenia (15635645), Depressive disorder (19596122) |
| ENSG00000077092 | *RARB* | 5 | Schizophrenia (19786960), Schizophrenia (18282690), Schizophrenia (16081509), Schizophrenia (11972448), Schizophrenia (16687441) |
| ENSG00000100302 | *RASD2* | 3 | Schizophrenia (23555897), Schizophrenia (18571626), Schizophrenia (26228524) |
| ENSG00000078328 | *RBFOX1* | 7 | Autism Spectrum Disorders (26687839), Schizophrenia (26092620), Bipolar Disorder (19567891), Bipolar Disorder (19308021), Autism Spectrum Disorders (24290388), Attention deficit hyperactivity disorder (19546859), Autism Spectrum Disorders (22952857) |
| ENSG00000088320 | *REM1* | 9 | Unipolar Depression (15213704), Unipolar Depression (2521654), Unipolar Depression (9464197), Unipolar Depression (2963052), Major Depressive Disorder (11172871), Depressive disorder (19654034), Unipolar Depression (1790261), Major Depressive Disorder (9268775), Depressive disorder (15925700) |
| ENSG00000143839 | *REN* | 7 | Depressive disorder (17506223), Bipolar Disorder (17290801), Major Depressive Disorder (27262302), Depressive disorder (19734157), Depressive disorder (17499413), Depressive disorder (22688325), Depressive disorder (15576437) |
| ENSG00000102032 | *RENBP* | 3 | Schizophrenia (23868656), Schizophrenia (25768029), Schizophrenia (24043878) |
| ENSG00000111783 | *RFX4* | 3 | Bipolar Disorder (15940297), Bipolar Disorder (17510980), Bipolar Disorder (19328558) |
| ENSG00000117152 | *RGS4* | 55 | Bipolar Disorder (15660667), Schizophrenia (17055463), Schizophrenia (18804346), Schizophrenia (16508931), Schizophrenia (12436019), Schizophrenia (16905560), Schizophrenia (26910404), Schizophrenia (16176390), Schizophrenia (17006672), Schizophrenia (16791139), Schizophrenia (17588543), Schizophrenia (15381923), Bipolar Disorder (16380905), Schizophrenia (14732600), Schizophrenia (18262772), Schizophrenia (19282471), Schizophrenia (15182322), Bipolar Disorder (20414142), Bipolar Disorder (17410640), Schizophrenia (15274033), Schizophrenia (16904822), Schizophrenia (16631129), Schizophrenia (18414510), Schizophrenia (19937977), Schizophrenia (18195713), Schizophrenia (18204343), Schizophrenia (17722013), Schizophrenia (16978587), Schizophrenia (18719894), Schizophrenia (16604300), Schizophrenia (23332465), Schizophrenia (12023979), Schizophrenia (16526029), Schizophrenia (19367581), Schizophrenia (16612196), Schizophrenia (17408693), Schizophrenia (16860780), Schizophrenia (18622782), Schizophrenia (16469939), Schizophrenia (11326297), Schizophrenia (18493969), Bipolar Disorder (17239488), Schizophrenia (21910931), Schizophrenia (16082709), Bipolar Disorder (18685145), Schizophrenia (15744031), Major Depressive Disorder (18434012), Schizophrenia (17515439), Schizophrenia (14755443), Schizophrenia (17092693), Schizophrenia (17301167), Schizophrenia (21712773), Schizophrenia (17071056), Bipolar Disorder (17106420), Schizophrenia (18583979) |
| ENSG00000143248 | *RGS5* | 2 | Schizophrenia (18262772), Bipolar Disorder (19488044) |
| ENSG00000108370 | *RGS9* | 3 | Schizophrenia (18548510), Schizophrenia (17318883), Schizophrenia (16786561) |
| ENSG00000145491 | *ROPN1L* | 2 | Schizophrenia (12497613), Schizophrenia (10924404) |
| ENSG00000185483 | *ROR1* | 2 | Bipolar Disorder (21494683), Bipolar Disorder (19488044) |
| ENSG00000069667 | *RORA* | 11 | Depressive disorder (20800221), Depressive disorder (19693801), Major Depressive Disorder (19846067), Bipolar Disorder (20072116), Bipolar Disorder (24150227), Depressive disorder (22538398), Bipolar Disorder (25789810), Autism Spectrum Disorders (26625251), Bipolar Disorder (19909500), Bipolar Disorder (21494683), Bipolar Disorder (24716566) |
| ENSG00000198963 | *RORB* | 3 | Schizophrenia (19839995), Bipolar Disorder (19909500), Bipolar Disorder (25789810) |
| ENSG00000103494 | *RPGRIP1L* | 2 | Bipolar Disorder (21254220), Schizophrenia (19844207) |
| ENSG00000124782 | *RREB1* | 2 | Autism Spectrum Disorders (23285124), Autism Spectrum Disorders (22009741) |
| ENSG00000115310 | *RTN4* | 14 | Bipolar Disorder (15820318), Schizophrenia (21563301), Schizophrenia (14741411), Schizophrenia (18583979), Schizophrenia (19054571), Schizophrenia (12425946), Schizophrenia (15661375), Schizophrenia (20071518), Schizophrenia (18948092), Schizophrenia (19165527), Schizophrenia (17022955), Schizophrenia (15234466), Schizophrenia (22320844), Schizophrenia (15953657) |
| ENSG00000159216 | *RUNX1* | 2 | Schizophrenia (19158815), Schizophrenia (18281018) |
| ENSG00000198626 | *RYR2* | 2 | Autism Spectrum Disorders (26742492), Autism Spectrum Disorders (21151189) |
| ENSG00000132330 | *SCLY* | 7 | Depressive disorder (2934456), Depressive disorder (20064128), Schizophrenia (14693349), Depressive disorder (17210818), Depressive disorder (15842033), Depressive disorder (20494447), Depressive disorder (21835094) |
| ENSG00000144285 | *SCN1A* | 2 | Autism Spectrum Disorders (22550089), Autism Spectrum Disorders (21572417) |
| ENSG00000136531 | *SCN2A* | 2 | Schizophrenia (24718902), Autism Spectrum Disorders (26637798) |
| ENSG00000196876 | *SCN8A* | 3 | Bipolar Disorder (20353942), Bipolar Disorder (18812204), Attention deficit hyperactivity disorder (19352218) |
| ENSG00000075213 | *SEMA3A* | 3 | Schizophrenia (16402134), Schizophrenia (17684500), Schizophrenia (12610647) |
| ENSG00000153993 | *SEMA3D* | 2 | Schizophrenia (20684831), Schizophrenia (19054571) |
| ENSG00000108387 | *SEPT4* | 2 | Schizophrenia (19165527), Schizophrenia (17644312) |
| ENSG00000106366 | *SERPINE1* | 3 | Major Depressive Disorder (18794724), Depressive disorder (23979607), Attention deficit hyperactivity disorder (21812103) |
| ENSG00000099381 | *SETD1A* | 3 | Schizophrenia (26974950), Autism Spectrum Disorders (26938441), Schizophrenia (24853937) |
| ENSG00000181555 | *SETD2* | 4 | Schizophrenia (16632332), Autism Spectrum Disorders (26084711), Autism Spectrum Disorders (26637798), Schizophrenia (19367581) |
| ENSG00000100095 | *SEZ6L* | 2 | Bipolar Disorder (22920719), Autism Spectrum Disorders (26204995) |
| ENSG00000061936 | *SFSWAP* | 2 | Autism Spectrum Disorders (24781735), Schizophrenia (24094118) |
| ENSG00000102683 | *SGCG* | 3 | Depressive disorder (20376317), Schizophrenia (23286280), Schizophrenia (23528911) |
| ENSG00000118515 | *SGK1* | 2 | Major Depressive Disorder (25705664), Unipolar Depression (22832853) |
| ENSG00000129214 | *SHBG* | 2 | Schizophrenia (15696539), Depressive disorder (23602133) |
| ENSG00000176974 | *SHMT1* | 2 | Schizophrenia (20977478), Schizophrenia (19223009) |
| ENSG00000120725 | *SIL1* | 2 | Autism Spectrum Disorders (22574686), Autism Spectrum Disorders (25432440) |
| ENSG00000096717 | *SIRT1* | 7 | Bipolar Disorder (20977650), Bipolar Disorder (21349544), Major Depressive Disorder (20451257), Schizophrenia (21882241), Major Depressive Disorder (26176920), Bipolar Disorder (27424200), Depressive disorder (26509718) |
| ENSG00000064651 | *SLC12A2* | 9 | Schizophrenia (22385968), Schizophrenia (24695712), Schizophrenia (23921125), Schizophrenia (20819979), Schizophrenia (22643131), Schizophrenia (21795557), Depressive disorder (25529063), Schizophrenia (19023125), Schizophrenia (21237234) |
| ENSG00000124140 | *SLC12A5* | 3 | Schizophrenia (20819979), Schizophrenia (22496567), Schizophrenia (21795557) |
| ENSG00000091664 | *SLC17A6* | 4 | Schizophrenia (19839996), Schizophrenia (19228977), Schizophrenia (18155679), Schizophrenia (20541370) |
| ENSG00000104888 | *SLC17A7* | 13 | Schizophrenia (15653259), Schizophrenia (18155679), Schizophrenia (19839996), Schizophrenia (21145444), Major Depressive Disorder (23706640), Major Depressive Disorder (24743714), Schizophrenia (15124027), Schizophrenia (19720501), Schizophrenia (21396926), Bipolar Disorder (20079890), Schizophrenia (23356950), Schizophrenia (22197296), Schizophrenia (19917116) |
| ENSG00000036565 | *SLC18A1* | 5 | Bipolar Disorder (20419435), Schizophrenia (18451639), Schizophrenia (18249496), Bipolar Disorder (16936705), Depressive disorder (19204725) |
| ENSG00000165646 | *SLC18A2* | 18 | Schizophrenia (8825897), Bipolar Disorder (18249496), Depressive disorder (18797399), Schizophrenia (17427184), Schizophrenia (18451639), Depressive disorder (17018806), Schizophrenia (11443533), Bipolar Disorder (16936705), Bipolar Disorder (19328558), Schizophrenia (16139173), Depressive disorder (19468717), Schizophrenia (23932573), Schizophrenia (20815037), Depressive disorder (23697793), Depressive disorder (16806099), Schizophrenia (18045777), Depressive disorder (18329002), Major Depressive Disorder (24525708) |
| ENSG00000110436 | *SLC1A2* | 21 | Schizophrenia (26057049), Bipolar Disorder (23023733), Bipolar Disorder (23706640), Schizophrenia (9602129), Schizophrenia (21281445), Unipolar Depression (16230605), Schizophrenia (24076156), Schizophrenia (11850151), Schizophrenia (15296513), Schizophrenia (24560881), Depressive disorder (21779782), Schizophrenia (19716271), Schizophrenia (22728822), Major Depressive Disorder (23415275), Major Depressive Disorder (24652383), Bipolar Disorder (24518437), Schizophrenia (25406999), Schizophrenia (23356950), Schizophrenia (26459047), Schizophrenia (19660525), Depressive disorder (21161710) |
| ENSG00000115902 | *SLC1A4* | 3 | Schizophrenia (18638388), Schizophrenia (10994645), Schizophrenia (17106422) |
| ENSG00000105281 | *SLC1A5* | 2 | Schizophrenia (18638388), Schizophrenia (9034006) |
| ENSG00000105143 | *SLC1A6* | 3 | Schizophrenia (11850151), Schizophrenia (17221839), Schizophrenia (17982252) |
| ENSG00000188467 | *SLC24A5* | 2 | Schizophrenia (20691427), Schizophrenia (19944766) |
| ENSG00000115840 | *SLC25A12* | 6 | Autism Spectrum Disorders (21691713), Autism Spectrum Disorders (17894412), Autism Spectrum Disorders (18607376), Schizophrenia (17693006), Autism Spectrum Disorders (25921325), Autism Spectrum Disorders (25663199) |
| ENSG00000178537 | *SLC25A20* | 2 | Bipolar Disorder (23140658), Schizophrenia (18163426) |
| ENSG00000153291 | *SLC25A27* | 2 | Schizophrenia (17066476), Schizophrenia (21332312) |
| ENSG00000101438 | *SLC32A1* | 3 | Schizophrenia (21145444), Schizophrenia (21396926), Schizophrenia (17471287) |
| ENSG00000157103 | *SLC6A1* | 9 | Attention deficit hyperactivity disorder (18821565), Schizophrenia (11156808), Attention deficit hyperactivity disorder (17440935), Depressive disorder (17164814), Schizophrenia (24361861), Schizophrenia (25312391), Schizophrenia (18923069), Depressive disorder (20016099), Schizophrenia (17471287) |
| ENSG00000103546 | *SLC6A2* | 70 | Attention deficit hyperactivity disorder (19698724), Major Depressive Disorder (26960194), Major Depressive Disorder (11927173), Attention deficit hyperactivity disorder (22297068), Unipolar Depression (24374057), Attention deficit hyperactivity disorder (23052569), Attention deficit hyperactivity disorder (18591486), Depressive disorder (25650523), Unipolar Depression (26061302), Attention deficit hyperactivity disorder (18163388), Attention deficit hyperactivity disorder (19387424), Bipolar Disorder (7615302), Depressive disorder (20070865), Depressive disorder (24655776), Attention deficit hyperactivity disorder (18821565), Attention deficit hyperactivity disorder (18937309), Attention deficit hyperactivity disorder (17994190), Major Depressive Disorder (15539861), Major Depressive Disorder (15118352), Attention deficit hyperactivity disorder (18821564), Attention deficit hyperactivity disorder (19721846), Schizophrenia (21396926), Unipolar Depression (24312678), Major Depressive Disorder (17353941), Unipolar Depression (10512149), Major Depressive Disorder (25512257), Major Depressive Disorder (23648227), Unipolar Depression (17917281), Depressive disorder (16722247), Major Depressive Disorder (20588071), Attention deficit hyperactivity disorder (19858760), Attention deficit hyperactivity disorder (25554436), Attention deficit hyperactivity disorder (23266789), Attention deficit hyperactivity disorder (23609393), Bipolar Disorder (17654409), Attention deficit hyperactivity disorder (19685476), Attention deficit hyperactivity disorder (17146058), Depressive disorder (23636256), Schizophrenia (20691427), Depressive disorder (23969988), Bipolar Disorder (12097806), Major Depressive Disorder (19105200), Major Depressive Disorder (19095219), Major Depressive Disorder (25739526), Attention deficit hyperactivity disorder (15322419), Depressive disorder (19468717), Attention deficit hyperactivity disorder (15950014), Schizophrenia (20543991), Depressive disorder (25990886), Major Depressive Disorder (25827644), Major Depressive Disorder (19564048), Attention deficit hyperactivity disorder (17876324), Attention deficit hyperactivity disorder (18214863), Attention deficit hyperactivity disorder (19352218), Depressive disorder (18973248), Unipolar Depression (22727709), Attention deficit hyperactivity disorder (21628343), Autism Spectrum Disorders (24381062), Attention deficit hyperactivity disorder (24942521), Attention deficit hyperactivity disorder (18937296), Unipolar Depression (18779921), Attention deficit hyperactivity disorder (26678348), Depressive disorder (18081710), Attention deficit hyperactivity disorder (14681910), Schizophrenia (19944766), Schizophrenia (25450228), Depressive disorder (18800064), Attention deficit hyperactivity disorder (15719398), Attention deficit hyperactivity disorder (15717291), Depressive disorder (25422957) |
| ENSG00000165970 | *SLC6A5* | 2 | Schizophrenia (22272310), Schizophrenia (18638388) |
| ENSG00000130876 | *SLC7A10* | 2 | Schizophrenia (21888942), Schizophrenia (18400471) |
| ENSG00000109062 | *SLC9A3R1* | 2 | Schizophrenia (19165527), Schizophrenia (19034380) |
| ENSG00000181804 | *SLC9A9* | 6 | Attention deficit hyperactivity disorder (18937294), Attention deficit hyperactivity disorder (18821565), Attention deficit hyperactivity disorder (20732626), Attention deficit hyperactivity disorder (21858920), Attention deficit hyperactivity disorder (25875332), Attention deficit hyperactivity disorder (20032819) |
| ENSG00000184347 | *SLIT3* | 2 | Major Depressive Disorder (21152026), Schizophrenia (15231749) |
| ENSG00000166311 | *SMPD1* | 2 | Schizophrenia (19054571), Depressive disorder (23770692) |
| ENSG00000132639 | *SNAP25* | 56 | Attention deficit hyperactivity disorder (25629685), Attention deficit hyperactivity disorder (17455213), Schizophrenia (18512733), Schizophrenia (22940547), Attention deficit hyperactivity disorder (25445064), Schizophrenia (19806613), Attention deficit hyperactivity disorder (20002519), Attention deficit hyperactivity disorder (16088329), Attention deficit hyperactivity disorder (17187001), Attention deficit hyperactivity disorder (17427194), Attention deficit hyperactivity disorder (16135997), Attention deficit hyperactivity disorder (20599404), Attention deficit hyperactivity disorder (11920846), Attention deficit hyperactivity disorder (19352218), Attention deficit hyperactivity disorder (27627841), Attention deficit hyperactivity disorder (19099826), Attention deficit hyperactivity disorder (21996783), Schizophrenia (19132710), Bipolar Disorder (19125158), Schizophrenia (19827316), Attention deficit hyperactivity disorder (17325713), Attention deficit hyperactivity disorder (24362847), Attention deficit hyperactivity disorder (17980763), Attention deficit hyperactivity disorder (19858760), Autism Spectrum Disorders (21497654), Attention deficit hyperactivity disorder (20679152), Attention deficit hyperactivity disorder (12232787), Schizophrenia (18347838), Bipolar Disorder (23064108), Attention deficit hyperactivity disorder (15717291), Depressive disorder (14708030), Major Depressive Disorder (25650683), Attention deficit hyperactivity disorder (23872233), Schizophrenia (15823421), Attention deficit hyperactivity disorder (26821215), Attention deficit hyperactivity disorder (21756448), Attention deficit hyperactivity disorder (19695183), Attention deficit hyperactivity disorder (12660803), Schizophrenia (22264613), Bipolar Disorder (18726138), Depressive disorder (19679075), Attention deficit hyperactivity disorder (10889551), Attention deficit hyperactivity disorder (17501935), Attention deficit hyperactivity disorder (17979583), Attention deficit hyperactivity disorder (18821565), Attention deficit hyperactivity disorder (15007392), Attention deficit hyperactivity disorder (22825876), Schizophrenia (20333500), Schizophrenia (10089007), Attention deficit hyperactivity disorder (15719398), Schizophrenia (19193342), Attention deficit hyperactivity disorder (15578613), Attention deficit hyperactivity disorder (19721846), Schizophrenia (12814864), Schizophrenia (11287790), Attention deficit hyperactivity disorder (22584804) |
| ENSG00000145335 | *SNCA* | 4 | Depressive disorder (21271299), Depressive disorder (18800064), Depressive disorder (25921825), Depressive disorder (26077166) |
| ENSG00000134243 | *SORT1* | 2 | Schizophrenia (26839058), Depressive disorder (26556286) |
| ENSG00000115904 | *SOS1* | 2 | Schizophrenia (23720743), Bipolar Disorder (20436929) |
| ENSG00000171243 | *SOSTDC1* | 24 | Major Depressive Disorder (10910786), Schizophrenia (20347268), Schizophrenia (11146756), Major Depressive Disorder (19386277), Attention deficit hyperactivity disorder (16950213), Single major depressive episode (21693183), Schizophrenia (19329282), Major Depressive Disorder (22078646), Bipolar Disorder (18085546), Schizophrenia (18562188), Schizophrenia (22723893), Schizophrenia (20664580), Attention deficit hyperactivity disorder (25201318), Major Depressive Disorder (19347611), Single major depressive episode (21849985), Bipolar Disorder (24589068), Attention deficit hyperactivity disorder (25284319), Autism Spectrum Disorders (18317467), Depressive disorder (24324616), Bipolar Disorder (23437284), Schizophrenia (20977650), Schizophrenia (20661937), Bipolar Disorder (18165970), Schizophrenia (24439302) |
| ENSG00000185591 | *SP1* | 4 | Schizophrenia (23540600), Bipolar Disorder (23941741), Schizophrenia (17192956), Schizophrenia (17786189) |
| ENSG00000105866 | *SP4* | 4 | Bipolar Disorder (22017217), Schizophrenia (20634195), Bipolar Disorder (19401786), Bipolar Disorder (26049820) |
| ENSG00000116096 | *SPR* | 2 | Bipolar Disorder (19415819), Schizophrenia (20846490) |
| ENSG00000187678 | *SPRY4* | 2 | Schizophrenia (18298822), Schizophrenia (23542694) |
| ENSG00000145545 | *SRD5A1* | 3 | Bipolar Disorder (21570127), Depressive disorder (15033426), Schizophrenia (20672519) |
| ENSG00000167978 | *SRRM2* | 4 | Depressive disorder (25178406), Schizophrenia (20927331), Autism Spectrum Disorders (26687839), Schizophrenia (24571439) |
| ENSG00000157005 | *SST* | 17 | Unipolar Depression (17130427), Schizophrenia (24674775), Schizophrenia (22937123), Schizophrenia (24361861), Bipolar Disorder (17239033), Bipolar Disorder (17239488), Major Depressive Disorder (21226980), Schizophrenia (17471287), Major Depressive Disorder (21912391), Schizophrenia (21745723), Unipolar Depression (24690741), Schizophrenia (25464914), Schizophrenia (19121517), Schizophrenia (24636039), Schizophrenia (19804960), Schizophrenia (18203698), Bipolar Disorder (19328558) |
| ENSG00000070526 | *ST6GALNAC1* | 2 | Schizophrenia (21908516), Bipolar Disorder (24389572) |
| ENSG00000140557 | *ST8SIA2* | 11 | Schizophrenia (24070986), Schizophrenia (17126533), Schizophrenia (16229822), Schizophrenia (24057454), Schizophrenia (22693595), Schizophrenia (26418860), Bipolar Disorder (24651862), Schizophrenia (16969366), Schizophrenia (23354723), Bipolar Disorder (25129258), Autism Spectrum Disorders (27105834) |
| ENSG00000113532 | *ST8SIA4* | 2 | Schizophrenia (23354723), Schizophrenia (16229822) |
| ENSG00000010327 | *STAB1* | 2 | Bipolar Disorder (25136889), Schizophrenia (19367581) |
| ENSG00000168610 | *STAT3* | 2 | Depressive disorder (20410598), Depressive disorder (25760924) |
| ENSG00000117632 | *STMN1* | 3 | Depressive disorder (21438138), Major Depressive Disorder (23185405), Depressive disorder (23668904) |
| ENSG00000140022 | *STON2* | 2 | Schizophrenia (21407139), Schizophrenia (23785397) |
| ENSG00000106089 | *STX1A* | 4 | Schizophrenia (15219469), Attention deficit hyperactivity disorder (25445064), Attention deficit hyperactivity disorder (22939005), Schizophrenia (18512733) |
| ENSG00000130540 | *SULT4A1* | 5 | Schizophrenia (19125109), Schizophrenia (18823757), Schizophrenia (16152568), Schizophrenia (25340730), Schizophrenia (17728668) |
| ENSG00000131018 | *SYNE1* | 5 | Recurrent major depressive episodes (22565781), Depressive disorder (24135662), Bipolar Disorder (24387768), Autism Spectrum Disorders (23352163), Bipolar Disorder (23820096) |
| ENSG00000171992 | *SYNPO* | 2 | Schizophrenia (26405221), Schizophrenia (20385374) |
| ENSG00000102003 | *SYP* | 12 | Schizophrenia (15694236), Depressive disorder (12391607), Attention deficit hyperactivity disorder (25487813), Bipolar Disorder (19945534), Schizophrenia (7477874), Schizophrenia (25315318), Attention deficit hyperactivity disorder (16894395), Schizophrenia (10755070), Schizophrenia (10089007), Schizophrenia (11483314), Schizophrenia (22348818), Attention deficit hyperactivity disorder (16082702) |
| ENSG00000006128 | *TAC1* | 13 | Schizophrenia (15845098), Depressive disorder (22155476), Depressive disorder (15157992), Attention deficit hyperactivity disorder (9369304), Depressive disorder (12907922), Depressive disorder (12692775), Schizophrenia (20347265), Depressive disorder (24705689), Depressive disorder (21912391), Depressive disorder (16594257), Depressive disorder (17477887), Major Depressive Disorder (12203048), Depressive disorder (10716242) |
| ENSG00000115353 | *TACR1* | 10 | Bipolar Disorder (19204064), Bipolar Disorder (24817687), Schizophrenia (17443717), Depressive disorder (12692775), Depressive disorder (24705689), Schizophrenia (16815618), Bipolar Disorder (19415819), Depressive disorder (20112009), Depressive disorder (16594257), Depressive disorder (17974009) |
| ENSG00000169836 | *TACR3* | 4 | Schizophrenia (18287949), Schizophrenia (20597024), Schizophrenia (19879867), Depressive disorder (23697793) |
| ENSG00000198650 | *TAT* | 2 | Depressive disorder (20957648), Depressive disorder (21998007) |
| ENSG00000109436 | *TBC1D9* | 7 | Schizophrenia (18543120), Major Depressive Disorder (18550244), Schizophrenia (16386826), Schizophrenia (17113599), Schizophrenia (20143052), Schizophrenia (20195292), Schizophrenia (20060871) |
| ENSG00000112592 | *TBP* | 3 | Schizophrenia (16054804), Schizophrenia (21601610), Schizophrenia (19566714) |
| ENSG00000196628 | *TCF4* | 36 | Bipolar Disorder (10712198), Schizophrenia (20434134), Bipolar Disorder (23786914), Schizophrenia (10813808), Schizophrenia (25217366), Bipolar Disorder (10395212), Schizophrenia (24058414), Schizophrenia (23129290), Autism Spectrum Disorders (23640545), Schizophrenia (24718684), Schizophrenia (24594265), Schizophrenia (25858580), Schizophrenia (24275585), Schizophrenia (26010163), Schizophrenia (20673877), Schizophrenia (21932083), Bipolar Disorder (11526470), Schizophrenia (19571808), Schizophrenia (21946175), Schizophrenia (27305091), Schizophrenia (24413739), Schizophrenia (21228604), Schizophrenia (26343600), Schizophrenia (23894747), Schizophrenia (22832956), Schizophrenia (21791550), Schizophrenia (23249814), Schizophrenia (21812098), Bipolar Disorder (11377748), Schizophrenia (20421335), Schizophrenia (20934321), Schizophrenia (25658856), Schizophrenia (22451930), Schizophrenia (21543597), Schizophrenia (22781169), Schizophrenia (24686180) |
| ENSG00000156414 | *TDRD9* | 2 | Bipolar Disorder (19308021), Bipolar Disorder (20414141) |
| ENSG00000167074 | *TEF* | 2 | Unipolar Depression (24581835), Major Depressive Disorder (27964944) |
| ENSG00000164362 | *TERT* | 3 | Major Depressive Disorder (23668904), Major Depressive Disorder (24967945), Schizophrenia (26799699) |
| ENSG00000091513 | *TF* | 4 | Schizophrenia (21643746), Schizophrenia (18045615), Schizophrenia (17496814), Schizophrenia (19110265) |
| ENSG00000008196 | *TFAP2B* | 2 | Bipolar Disorder (16787706), Depressive disorder (19184334) |
| ENSG00000198959 | *TGM2* | 5 | Schizophrenia (18561261), Bipolar Disorder (22389694), Schizophrenia (26307914), Bipolar Disorder (21320252), Schizophrenia (20926141) |
| ENSG00000137801 | *THBS1* | 2 | Schizophrenia (22311024), Schizophrenia (21822266) |
| ENSG00000111602 | *TIMELESS* | 9 | Depressive disorder (20174623), Bipolar Disorder (18228528), Bipolar Disorder (20072116), Schizophrenia (19839995), Bipolar Disorder (24636202), Bipolar Disorder (17239488), Bipolar Disorder (17239033), Bipolar Disorder (24716566), Depressive disorder (19708722) |
| ENSG00000137462 | *TLR2* | 5 | Bipolar Disorder (25790282), Bipolar Disorder (24882191), Bipolar Disorder (26795430), Schizophrenia (23644137), Schizophrenia (25687169) |
| ENSG00000164342 | *TLR3* | 2 | Schizophrenia (23587629), Depressive disorder (24391741) |
| ENSG00000067182 | *TNFRSF1A* | 7 | Schizophrenia (20659789), Depressive disorder (17094069), Depressive disorder (16458261), Schizophrenia (19193342), Depressive disorder (21194425), Depressive disorder (24094876), Depressive disorder (21097524) |
| ENSG00000028137 | *TNFRSF1B* | 9 | Depressive disorder (16458261), Schizophrenia (11126399), Schizophrenia (20842464), Depressive disorder (17094069), Schizophrenia (19193342), Major Depressive Disorder (26278479), Depressive disorder (24047966), Depressive disorder (24094876), Unipolar Depression (18081157) |
| ENSG00000116147 | *TNR* | 2 | Bipolar Disorder (9359972), Schizophrenia (19573479) |
| ENSG00000129167 | *TPH1* | 68 | Schizophrenia (17521439), Depressive disorder (15941494), Schizophrenia (17870198), Major Depressive Disorder (19590397), Bipolar Disorder (11472792), Unipolar Depression (24903772), Major Depressive Disorder (19032713), Unipolar Depression (22697203), Major Depressive Disorder (17066254), Major Depressive Disorder (18177948), Major Depressive Disorder (18332644), Schizophrenia (11324941), Major Depressive Disorder (23221997), Schizophrenia (16806098), Depressive disorder (19738481), Bipolar Disorder (10898906), Major Depressive Disorder (17692928), Major Depressive Disorder (21601290), Bipolar Disorder (15799788), Major Depressive Disorder (16979275), Major Depressive Disorder (12502014), Major Depressive Disorder (19500158), Major Depressive Disorder (16165107), Bipolar Disorder (15727488), Bipolar Disorder (11772685), Schizophrenia (18221792), Bipolar Disorder (17768266), Major Depressive Disorder (23157339), Schizophrenia (20144688), Major Depressive Disorder (19874868), Unipolar Depression (11426508), Single major depressive episode (11597824), Depressive disorder (23063133), Schizophrenia (16741719), Major Depressive Disorder (23597148), Bipolar Disorder (11578639), Unipolar Depression (15544576), Major Depressive Disorder (18982004), Depressive disorder (24495952), Bipolar Disorder (15627807), Major Depressive Disorder (15052272), Attention deficit hyperactivity disorder (21906006), Schizophrenia (18583979), Depressive disorder (14998306), Schizophrenia (19367581), Schizophrenia (19526457), Bipolar Disorder (12860364), Bipolar Disorder (16240163), Schizophrenia (12210276), Single major depressive episode (20471034), Single major depressive episode (18977032), Schizophrenia (11343864), Bipolar Disorder (9435758), Schizophrenia (10899755), Depressive disorder (23512949), Schizophrenia (22655589), Schizophrenia (15211625), Major Depressive Disorder (16314762), Major Depressive Disorder (15274037), Attention deficit hyperactivity disorder (16389593), Schizophrenia (22053918), Schizophrenia (20046510), Major Depressive Disorder (20945066), Depressive disorder (15729745), Bipolar Disorder (12366879), Major Depressive Disorder (16302021), Attention deficit hyperactivity disorder (20213726), Major Depressive Disorder (15475734) |
| ENSG00000139287 | *TPH2* | 91 | Schizophrenia (20623453), Schizophrenia (15840421), Schizophrenia (21399903), Schizophrenia (22655589), Major Depressive Disorder (25955598), Major Depressive Disorder (26745768), Schizophrenia (21396719), Unipolar Depression (23467366), Depressive disorder (25214390), Bipolar Disorder (26365518), Schizophrenia (20144688), Major Depressive Disorder (16192985), Depressive disorder (23063133), Major Depressive Disorder (26057341), Major Depressive Disorder (24376086), Major Depressive Disorder (19272410), Depressive disorder (21257271), Bipolar Disorder (17768266), Schizophrenia (16240163), Unipolar Depression (18180764), Depressive disorder (17950541), Attention deficit hyperactivity disorder (18213624), Bipolar Disorder (17905754), Bipolar Disorder (16806105), Schizophrenia (16436194), Attention deficit hyperactivity disorder (18163388), Depressive disorder (22868061), Major Depressive Disorder (24196946), Major Depressive Disorder (16203956), Bipolar Disorder (19352219), Unipolar Depression (15124006), Major Depressive Disorder (19588223), Attention deficit hyperactivity disorder (23461725), Major Depressive Disorder (21937687), Major Depressive Disorder (17217922), Depressive disorder (24863038), Attention deficit hyperactivity disorder (20213726), Depressive disorder (22826343), Unipolar Depression (17251907), Attention deficit hyperactivity disorder (20470849), Major Depressive Disorder (22693556), Major Depressive Disorder (19162119), Attention deficit hyperactivity disorder (18427560), Unipolar Depression (19120094), Depressive disorder (19125159), Major Depressive Disorder (21620479), Major Depressive Disorder (19734157), Attention deficit hyperactivity disorder (25875332), Attention deficit hyperactivity disorder (16116490), Schizophrenia (20938755), Unipolar Depression (19590397), Attention deficit hyperactivity disorder (19894072), Major Depressive Disorder (26386440), Unipolar Depression (15629698), Bipolar Disorder (15727488), Depressive disorder (19548263), Schizophrenia (25073638), Depressive disorder (17239033), Depressive disorder (23547810), Depressive disorder (22721547), Depressive disorder (15941494), Depressive disorder (20515362), Depressive disorder (25089765), Attention deficit hyperactivity disorder (18821565), Bipolar Disorder (18797398), Unipolar Depression (20738857), Bipolar Disorder (25152196), Attention deficit hyperactivity disorder (20921119), Depressive disorder (21765945), Bipolar Disorder (17015812), Depressive disorder (21873838), Bipolar Disorder (19328558), Major Depressive Disorder (17692928), Bipolar Disorder (19800079), Bipolar Disorder (17167340), Depressive disorder (19679166), Bipolar Disorder (21438144), Depressive disorder (17892388), Major Depressive Disorder (23336047), Autism Spectrum Disorders (23628433), Depressive disorder (23510446), Bipolar Disorder (21085052), Depressive disorder (20043001), Autism Spectrum Disorders (20446882), Attention deficit hyperactivity disorder (15940290), Major Depressive Disorder (15052272), Attention deficit hyperactivity disorder (19352218), Depressive disorder (17408646), Attention deficit hyperactivity disorder (16894395), Depressive disorder (22826344), Attention deficit hyperactivity disorder (19270759) |
| ENSG00000170893 | *TRH* | 7 | Depressive disorder (2543997), Depressive disorder (6412261), Major Depressive Disorder (7798461), Unipolar Depression (6418677), Unipolar Depression (6461684), Depressive disorder (19078951), Depressive disorder (25028079) |
| ENSG00000138741 | *TRPC3* | 2 | Bipolar Disorder (23602965), Bipolar Disorder (22420591) |
| ENSG00000103197 | *TSC2* | 7 | Autism Spectrum Disorders (27052171), Autism Spectrum Disorders (21115397), Autism Spectrum Disorders (25155956), Schizophrenia (9928911), Autism Spectrum Disorders (21403110), Autism Spectrum Disorders (20927644), Autism Spectrum Disorders (21328568) |
| ENSG00000157570 | *TSPAN18* | 3 | Schizophrenia (26016498), Schizophrenia (22037552), Schizophrenia (23505562) |
| ENSG00000135452 | *TSPAN31* | 2 | Depressive disorder (19272208), Attention deficit hyperactivity disorder (25656289) |
| ENSG00000127324 | *TSPAN8* | 2 | Schizophrenia (20052686), Attention deficit hyperactivity disorder (21276201) |
| ENSG00000100300 | *TSPO* | 6 | Major Depressive Disorder (26556688), Bipolar Disorder (23942012), Schizophrenia (25560467), Schizophrenia (11215759), Depressive disorder (22348616), Schizophrenia (26472628) |
| ENSG00000118271 | *TTR* | 6 | Depressive disorder (16971399), Schizophrenia (16716350), Depressive disorder (15009661), Schizophrenia (19703508), Depressive disorder (19665514), Depressive disorder (4038761) |
| ENSG00000198431 | *TXNRD1* | 4 | Bipolar Disorder (15450783), Attention deficit hyperactivity disorder (25600112), Bipolar Disorder (16699605), Autism Spectrum Disorders (26052927) |
| ENSG00000077498 | *TYR* | 6 | Schizophrenia (8383424), Schizophrenia (11352574), Schizophrenia (1677600), Schizophrenia (2611578), Bipolar Disorder (7903509), Schizophrenia (8475213) |
| ENSG00000011600 | *TYROBP* | 3 | Schizophrenia (26332043), Schizophrenia (24564241), Schizophrenia (21421043) |
| ENSG00000114062 | *UBE3A* | 7 | Autism Spectrum Disorders (22645313), Autism Spectrum Disorders (23468062), Autism Spectrum Disorders (25687563), Autism Spectrum Disorders (21624971), Autism Spectrum Disorders (16905559), Autism Spectrum Disorders (21029865), Autism Spectrum Disorders (17415598) |
| ENSG00000175567 | *UCP2* | 2 | Schizophrenia (17066476), Schizophrenia (22001364) |
| ENSG00000152332 | *UHMK1* | 5 | Schizophrenia (19747464), Schizophrenia (18414510), Schizophrenia (21399567), Schizophrenia (16978587), Schizophrenia (19451863) |
| ENSG00000182168 | *UNC5C* | 2 | Schizophrenia (19850283), Schizophrenia (24956103) |
| ENSG00000010256 | *UQCRC1* | 2 | Schizophrenia (19110265), Schizophrenia (19165527) |
| ENSG00000109189 | *USP46* | 2 | Schizophrenia (20111060), Major Depressive Disorder (21663972) |
| ENSG00000146469 | *VIP* | 3 | Bipolar Disorder (20072116), Schizophrenia (24674775), Schizophrenia (24636039) |
| ENSG00000106018 | *VIPR2* | 9 | Bipolar Disorder (20072116), Attention deficit hyperactivity disorder (26304033), Schizophrenia (24002029), Schizophrenia (21285140), Schizophrenia (23073313), Schizophrenia (24220567), Schizophrenia (21721910), Schizophrenia (24794882), Schizophrenia (21346763) |
| ENSG00000147852 | *VLDLR* | 5 | Schizophrenia (12363388), Schizophrenia (17936586), Unipolar Depression (20493228), Schizophrenia (17261317), Depressive disorder (15010349) |
| ENSG00000198554 | *WDHD1* | 2 | Schizophrenia (14755439), Schizophrenia (16511840) |
| ENSG00000095397 | *WHRN* | 4 | Bipolar Disorder (21507135), Bipolar Disorder (26297903), Bipolar Disorder (19308021), Bipolar Disorder (20414141) |
| ENSG00000125084 | *WNT1* | 2 | Schizophrenia (10427605), Autism Spectrum Disorders (24002087) |
| ENSG00000105989 | *WNT2* | 2 | Schizophrenia (20492734), Autism Spectrum Disorders (21575668) |
| ENSG00000073050 | *XRCC1* | 3 | Schizophrenia (26824244), Schizophrenia (26554302), Schizophrenia (17961713) |
| ENSG00000128245 | *YWHAH* | 6 | Schizophrenia (15814194), Schizophrenia (11121172), Bipolar Disorder (19160447), Schizophrenia (10206237), Schizophrenia (19034380), Schizophrenia (16459651) |
| ENSG00000181722 | *ZBTB20* | 3 | Unipolar Depression (24694013), Autism Spectrum Disorders (23032108), Schizophrenia (19850283) |
| ENSG00000099904 | *ZDHHC8* | 13 | Schizophrenia (18075473), Schizophrenia (15489219), Schizophrenia (23403413), Schizophrenia (20661937), Schizophrenia (20468065), Schizophrenia (16150541), Schizophrenia (15184899), Schizophrenia (16225675), Schizophrenia (16860541), Schizophrenia (15992527), Schizophrenia (15631889), Schizophrenia (15582150), Schizophrenia (18583979) |
| ENSG00000125945 | *ZNF436* | 2 | Autism Spectrum Disorders (23285124), Autism Spectrum Disorders (22009741) |
| ENSG00000102870 | *ZNF629* | 2 | Autism Spectrum Disorders (22009741), Autism Spectrum Disorders (23285124) |

**Table S2. Parameters used in coalescent simulations. Maximum likelihood values estimated from a previous study (Gravel et al., 2011) were applied to ms simulator.**

| **Parameters** |  | **Values with**  **maximum likelihood** |
| --- | --- | --- |
| **Mutation rate** (per bp per generation) | | 2.35×10^-8^ |
| **Recombination rate** (per bp) |  | 8.418×10^-9^ |
| **Generation time** (years) |  | 25 |
| **Population size** | African ancestor | 7310 |
|  | African population | 14474 |
|  | Bottleneck population | 1861 |
|  | Initial European population | 1032 |
|  | Initial Asian population | 554 |
| **Growth rate** (per generation) | European population | 0.38 |
|  | Asian population | 0.48 |
| **Migration rate** (per generation) | African and bottlenack population | 15×10^-5^ |
|  | African and European population | 2.5×10^-5^ |
|  | African and Asian population | 0.78×10^-5^ |
|  | European and Asian population | 3.11×10^-5^ |
| **Time** (kya) | Growth in African ancestral  population | 148 |
|  | Divergence of Eurasian ancestral  population | 51 |
|  | Divergence of European and Asian populations | 23 |
| **ms syntax** | -t 6.8714 -r 2.461177 10000 -I 3 216 198 414 -n 1 1.980027 -n 2 4.62571 -n 3 6.206527 -eg 0 2 110.9014 -eg 0 3 140.0162 -ma x 0.731 0.228072 0.731 x 0.909364 0.228072 0.909364 x -eg 0.03146375 3 0 -em 0.03146375 3 1 0 -em 0.03146375 1 3 0 -ej 0.03146375 3 2 -en 0.03146375 2 0.2545828 -eg 0.03146375 2 0 -em 0.03146375 1 2 0 -em 0.03146375 2 1 0 -ema 0.03146375 3 x 4.386 x 4.386 x x x x x -ej 0.06986301 2 1 -en 0.2027397 1 1 | |

**Table S3. The estimated impact of amino acid substitutions occurring in the human lineage for positively selected genes related to psychiatric disorders (PD-PSGs).** Estimated deleterious substitutions, which could have had significant impacts on protein function (a score of less than −2.5 for Provean and a score of less than 0.05 for SIFT), are shown in bold type, and the residues in italics indicate that the given ancestral residue is conserved among 14 mammalian species except humans. Bayes Empirical Bayes (BEB) analysis shows the posterior probability of positive selection in the human lineage for given amino acid sites.

| **Genes** | **PAML *P*-value** | **Site Position** | **Ancestral residue** | **Derived residue** | **BEB** | **Provean score** | **SIFT score** |
| --- | --- | --- | --- | --- | --- | --- | --- |
| *CLSTN2* | 0.0031 | *794* | *Arg* | *Gln* | 0.926 | -0.26 | 0.5 |
| *FAT1* | 0.0064 | 628 | Gln | Arg | 0.614 | 0.64 | 1 |
|  |  | *667* | *Arg* | *His* | 0.701 | -0.54 | 0.18 |
|  |  | 756 | Ile | Val | 0.598 | -0.85 | **0.03** |
|  |  | 878 | Arg | Asn | 0.692 | -0.23 | 0.26 |
|  |  | *892* | *Tyr* | *Ser* | 0.706 | 0.17 | 0.55 |
|  |  | *1526* | *His* | *Pro* | 0.865 | -0.98 | 0.3 |
|  |  | *1527* | *Gln* | *Ala* | 0.979 | **-3.45** | **0** |
|  |  | *2196* | *Asn* | *Ser* | 0.702 | **-2.63** | **0.02** |
|  |  | *2276* | *Glu* | *Asp* | 0.701 | -2.02 | 0.16 |
|  |  | 2371 | Pro | Thr | 0.597 | **-3.67** | 0.05 |
|  |  | *2455* | *Arg* | *His* | 0.701 | -1.86 | 0.09 |
|  |  | 2618 | Ile | Val | 0.610 | -0.33 | 0.7 |
|  |  | *2690* | *Pro* | *Leu* | 0.703 | **-7.65** | **0.01** |
|  |  | *2973* | *Asn* | *Thr* | 0.704 | -1.1 | 0.39 |
|  |  | *4219* | *Glu* | *Lys* | 0.702 | -0.84 | 0.22 |
|  |  | 4459 | Asp | Asn | 0.697 | -0.73 | 0.12 |
| *SLC18A1* | 0.039 | 130 | Glu | Gly | 0.582 | **-3.68** | **0.05** |
|  |  | *136* | *Asn* | *Thr* | 0.842 | **-5.37** | 0.19 |

**Table S4. The results of the McDonald–Kreitman test for the three positively selected genes related to psychiatric disorders (PD-PSGs). *P*-values are calculated by Fisher’s exact test.**

| **Genes** |  | **Nonsynonymous** | **Synonymous** | ***P*-value** |
| --- | --- | --- | --- | --- |
| *CLSTN2* | Polymorphism within Human | 62 | 40 |  |
|  | Divergence between Human and Chimpanzee | 9 | 10 | 0.316 |
|  | Divergence between Human and macaque | 12 | 52 | < 0.01 |
|  |  |  |  |  |
| *FAT1* | Polymorphism within Human | 330 | 193 |  |
|  | Divergence between Human and Chimpanzee | 45 | 74 | < 0.001 |
|  | Divergence between Human and macaque | 155 | 441 | < 0.001 |
|  |  |  |  |  |
| *SLC18A1* | Polymorphism within Human | 63 | 24 |  |
|  | Divergence between Human and Chimpanzee | 7 | 10 | 0.0214 |
|  | Divergence between Human and macaque | 15 | 35 | < 0.001 |
